# Using combined CT-clinical radiomics models to identify epidermal growth factor receptor mutation subtypes in lung adenocarcinoma

**DOI:** 10.3389/fonc.2022.846589

**Published:** 2022-08-18

**Authors:** Ji-wen Huo, Tian-you Luo, Le Diao, Fa-jin Lv, Wei-dao Chen, Rui-ze Yu, Qi Li

**Affiliations:** ^1^ Department of Radiology, The First Affiliated Hospital of Chongqing Medical University, Chongqing, China; ^2^ Ocean International Center, The Infervision Medical Technology Co., Ltd., Beijing, China

**Keywords:** lung cancer, epidermal growth factor receptor, radiomics, computed tomography, machine learning

## Abstract

**Background:**

To investigate the value of computed tomography (CT)-based radiomics signatures in combination with clinical and CT morphological features to identify epidermal growth factor receptor (EGFR)-mutation subtypes in lung adenocarcinoma (LADC).

**Methods:**

From February 2012 to October 2019, 608 patients were confirmed with LADC and underwent chest CT scans. Among them, 307 (50.5%) patients had a positive *EGFR*-mutation and 301 (49.5%) had a negative *EGFR-*mutation. Of the *EGFR*-mutant patients, 114 (37.1%) had a 19del -mutation, 155 (50.5%) had a L858R-mutation, and 38 (12.4%) had other rare mutations. Three combined models were generated by incorporating radiomics signatures, clinical, and CT morphological features to predict *EGFR*-mutation status. Patients were randomly split into training and testing cohorts, 80% and 20%, respectively. Model 1 was used to predict positive and negative EGFR-mutation, model 2 was used to predict 19del and non-19del mutations, and model 3 was used to predict L858R and non-L858R mutations. The receiver operating characteristic curve and the area under the curve (AUC) were used to evaluate their performance.

**Results:**

For the three models, model 1 had AUC values of 0.969 and 0.886 in the training and validation cohorts, respectively. Model 2 had AUC values of 0.999 and 0.847 in the training and validation cohorts, respectively. Model 3 had AUC values of 0.984 and 0.806 in the training and validation cohorts, respectively.

**Conclusion:**

Combined models that incorporate radiomics signature, clinical, and CT morphological features may serve as an auxiliary tool to predict *EGFR*-mutation subtypes and contribute to individualized treatment for patients with LADC.

## Introduction

Lung cancer, the leading cause of cancer-associated mortality worldwide, is a heterogeneous disease whose incidence rate increases each year (1, 2). Approximately 85% of lung cancers are non-small-cell lung cancer, which has the most frequent histological subtype of lung adenocarcinoma (LADC) (2, 3).

Epidermal growth-factor receptor (EGFR), an effective therapeutic target for LADC, has been widely studied. Previous research has revealed that patients with an *EGFR*-mutation have a higher response rate to tyrosine kinase inhibitors (TKIs) and a longer progression-free survival (PFS) than those without an *EGFR*-mutation (4–6). The two most frequent mutant subtypes include *EGFR* exon 19 deletion (19del) and exon 21 mutation (L858R), which account for about 90% of all *EGFR* mutations (7). A few recent studies showed that 19del and L858R mutations had differing computed tomography (CT) and clinical characteristics (8). Additionally, several reports indicated that patients with a 19del-mutation had a longer PFS after receiving TKI treatment (9–12), while those with a L858R-mutation may be more responsive to chemotherapy or an immune checkpoint blockade treatment (13, 14). Therefore, the identification of *EGFR*-mutation subtypes is critical to select the appropriate targeted molecular therapy for patients with LADC. Biopsy and sequence testing are often used to analyze the *EGFR* genotype. However, detecting mutations can be hindered by the challenge of obtaining histologic samples, especially in unresectable or advanced tumors. Furthermore, biopsies may increase the risk of cancer metastasis and some patients with poor underlying conditions may not tolerate biopsy. In these situations, a noninvasive and easy-to-use method is needed to predict the *EGFR*-mutation status.

Radiomics, which allow for deeper excavation, prediction, and analysis using large volumes of high-throughput image data, is an auxiliary tool for clinical diagnosis and treatment (15). Previous studies have demonstrated that radiomics can distinguish tumors with *EGFR* mutations from those with wild-type *EGFR* (16, 17) and provides a noninvasive and quantified approach to gain insight into tumor heterogeneity. Unfortunately, our attempts to predict tumors with *EGFR* subtypes using radiomics features have not yet yielded results appropriate for use in the clinic. Some studies have shown that the prediction efficiency of tumors with *EGFR* mutations improved when clinical, CT, and radiomics features are combined in a model (18, 19). However, these studies lacked the necessary stratification to distinguish *EGFR*-mutation subtypes, and the related radiomics models have not been well evaluated.

The present study aimed to develop and validate several combined models that incorporate radiomics signatures, clinical, and CT morphological features to predict *EGFR*-mutation tumor status, focusing on the predominant 19del and L858R subtypes in patients with LADC.

## Materials and methods

### Patient data

This study was approved by the ethical committee of our institution, and the requirement for patient-informed consent was waived due to the retrospective nature of study. In total, 1095 patients admitted to our hospital from February 2012 to October 2019 were initially included. The inclusion criteria for target population were that the patients 1) were pathologically confirmed with LADC; 2) obtained *EGFR*-mutation testing results; 3) had completed clinical data, including age, gender, smoking history, and clinical cancer stage; and 4) had available chest contrast-enhanced CT data. Another 487 patients were excluded using the following criteria: 1) they received antitumor therapy prior to chest CT scans and EGFR gene detection; 2) they had multiple primary tumors; 3) their tumor had a boundary that could not be determined; 4) they had more than one *EGFR*-mutation subtype. There were 608 LADC patients finally included. Among them, 307 patients (50.5%) had an *EGFR*-mutation and 301 (49.5%) had a wild-type *EGFR*. Of the *EGFR*-mutant patients, 114 (37.1%) harbored a single 19del, 155 (50.5%) harbored a single L858R, and 38 (12.4%) harbored other rare mutations.

### Mutation detection

Molecular analyses were performed on tumor histologic or cytology samples. The *EGFR*-mutation statuses were detected using a real-time polymerase chain reaction-based amplification refractory mutation system using the Human *EGFR* Gene Mutations Detection Kit (Amoy Dx, Xiamen, China). Polymerase chain reaction included the 18 to 21 exons sequence and evaluated the 19del, L858R, T790M, 20 ins, G719X, S768I, and L861Q locus mutations.

### Image acquisition

All patients underwent chest contrast-enhanced CT scans using one of two CT systems (GE Healthcare, Milwaukee, WI, USA; Siemens Healthineers, Erlangen, Germany). All CT scans were performed at the end of inspiration, during a single breath-hold gap. The parameters were a 100–130 kVp tube voltage, 100–250 mA tube current, 5 mm/5 mm scanning slice thickness/interval, and a reconstruction thickness/interval of 0.625, 1 mm/0.625, 1 mm. After an unenhanced CT scanning, a non-ionic iodized contrast agent (300 mg iodide/mL) was injected through the antecubital vein with a double high-pressure injector at a dose of 1.5 mL/kg body weight (total volume 80–110 mL) at a flow rate of 3.0 mL/s. The contrast agent was followed by a 50 mL injection of saline solution. The arterial and delayed phase acquisition times were triggered at 30 and 120 s, respectively. Finally, the images were transferred to the picture archiving and communication system workstation system and exported to the DICOM format for image feature extraction.

### Evaluation of clinical and CT features

Images were analyzed by two radiologists, blinded to the clinical data, with more than 10 years of experience in chest CT interpretation. A consensus on differences in opinions was reached through consultation. Clinical indicators including age, gender, smoking history, and clinical staging were collected. The following CT features were observed: tumor location (central, involving the segmental or more-proximal bronchi; peripheral, involving the subsegmental or more-distal bronchi), tumor size (the longest tumor diameter in the lung window setting), margin (spiculation, lobulation), density (subsolid, tumor with ground-glass opacity [GGO]; solid, tumor without GGO), internal characteristics (air bronchogram, air-filled bronchus within the tumor; air space, air attenuation within the tumor including cavity and pseudo-cavity; necrosis, focal area of low attenuation without enhancement; calcification), external characteristics (vascular convergence sign, convergence of vessels toward the tumor; pleural retraction, linear or tentiform structures connected between the tumor and pleura), and associated findings (pleural effusion; lymphadenopathy, the short diameter of lymph node >1 cm); multiple pulmonary metastases (number of metastases >10).

### Image segmentation and feature extraction

All CT images were imported to the Infer Scholar Center platform (https://www.infervision.com/, Infer Scholar). The region of interest (ROI) was manually outlined by two radiologists with more than 10 years of experience in chest CT interpretation using the Infer Scholar Center platform, which was defined as the maximum contour of tumor on axial CT image (20). Five samples were randomly selected from patients with negative EGFR-mutation, 19del-mutation, L858R-mutation, and other rare mutations (20 samples together), respectively, for the ROI segmentation. To assess interobserver repeatability, the ROI segmentation was performed in a blinded way by the two radiologists. To evaluate intra observer repeatability, observer 1 repeated the ROI segmentation 4 weeks after the first assessment. Thereafter, the intra-class correlation coefficients (ICCs) were calculated to evaluate the stability and reproducibility of feature extraction and these features with both inter- and intra-observer ICC values greater than 0.75 were included in this study. Radiomics feature extraction was performed with P-y-Radiomics (https://pyradiomics.readthedocs.io/en/latest/), a flexible open-source platform capable of extracting a large panel of engineered features from medical images (20). For each accurately segmented tumor, the P-y-Radiomics algorithms were used to automatically extract tumor region features. A total of 919 Radiomics features and 18 clinical and CT factoring features were initially extracted.

### Model establishment and performance evaluation

For model 1, the least absolute shrinkage (LASSO) and selection operator algorithm were used to select the optimal predictive features and a five-fold cross-validation was used to select the best machine learning algorithm. Finally, we used the Gradient Boost Tree by combing radiomics, clinical, and CT morphological features to build model 1. For models 2 and 3, instead of feature selection, 919 Radiomics features and 18 clinical and CT features were initially included for obtaining a better performance. And then, we used light GBM algorithm to conduct feature screening and classification of model modeling. We first trained light GBM model and conducted feature importance ranking using Permutation Importance method, and selected important features through supervised learning for modeling prediction. The permutation feature importance is defined to be the decrease in a model score when a single feature value is randomly shuffled (21). Finally, we used 202 features whose importance score is greater than 0 to build model 2, including 29 first-order features, 4 shape 2D features, 159 advanced textural features (43 GLCM, 32 GLDM, 38 GLRLM, 35 GLSZM, 11 NGTDM) as well as 10 clinical and CT morphological features, and we used 358 features whose importance score is greater than 0 to build model 3, including 64 first-order features, 1 shape 2D features, 282 advanced textural features (106 GLCM, 54 GLDM, 56 GLRLM, 42 GLSZM, 24 NGTDM) as well as 11 clinical and CT morphological features.

**Figure 1:**
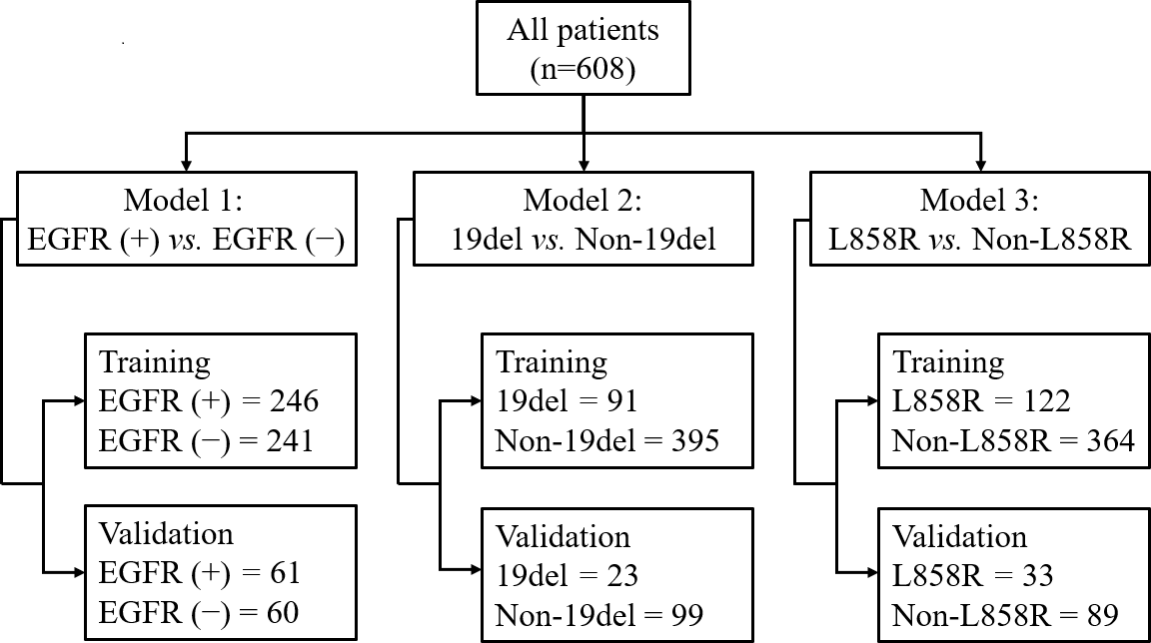
The detailed split-sample settings for each model.

**Figure 2:**
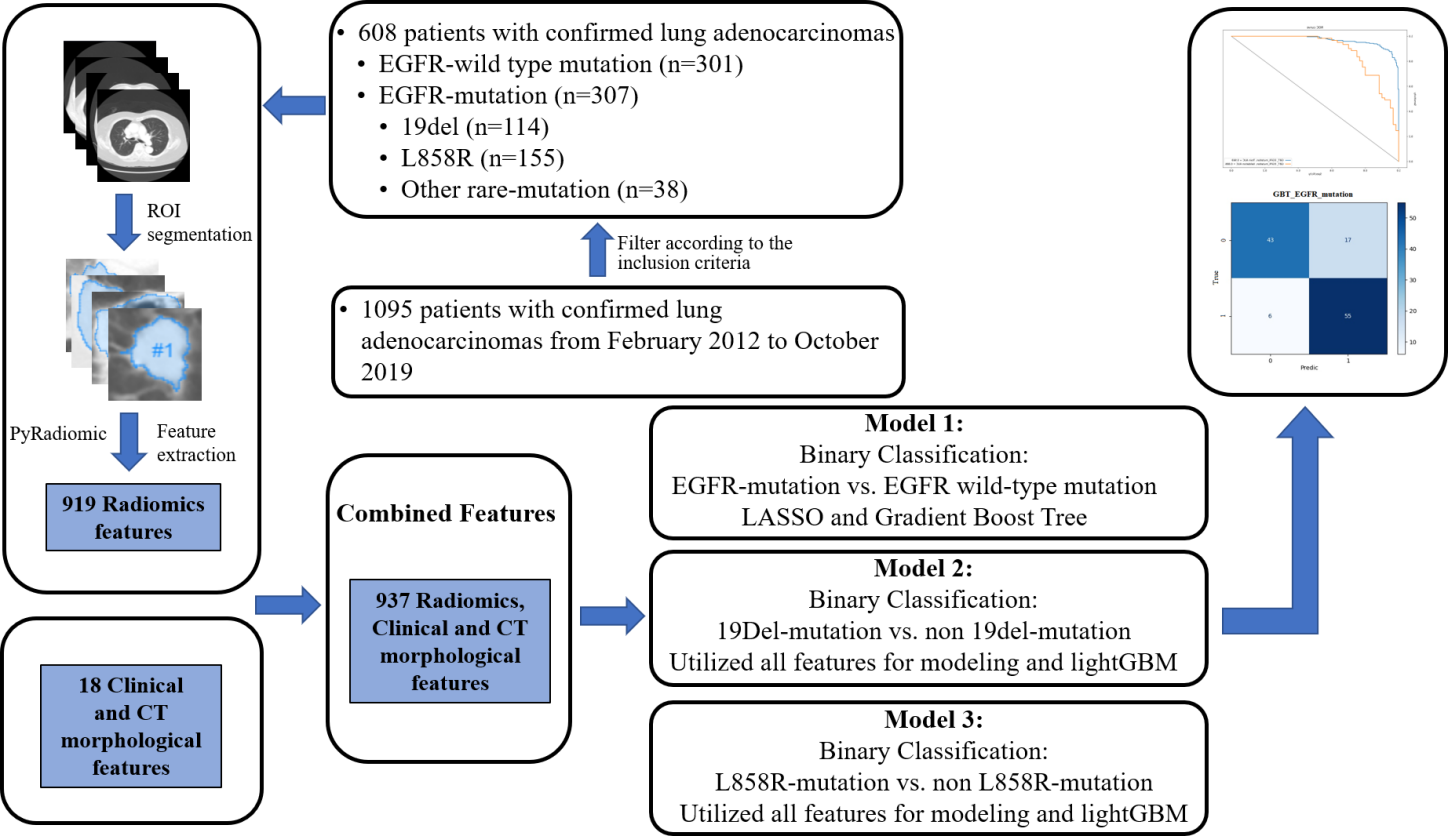
Study workflow.

In all models, patients were randomly split into training and testing cohorts, 80% and 20%, respectively. The detailed split-sample settings for each model are shown in **Figure 1**. Model 1 was used to identify positive and negative EGFR mutations, model 2 was used to distinguish 19del from non-19del mutations, and model 3 was used to determine L858R from non-L858R mutations in LADC patients. The workflow is shown in **Figure 2**. The receiver operating characteristic curve (ROC) and the area under the curve (AUC) of training and validation sets as well as the accuracy, sensitivity, and specificity in validation sets were used to evaluate the performance of three models.

### Statistical analysis

Statistical analyses were performed by using SPSS statistics (version 25; IBM, Armonk, NY, USA). The clinical and CT features between patients with positive and negative EGFR mutations, between patients with 19del and non-19del mutations, and between patients with L858R from non-L858R mutations were compared, respectively. Furthermore, for testing whether the background factors between cohorts were balanced, the clinical and CT features of patients in training and validation sets in each model were compared respectively. For continuous variables of clinical and CT morphological features, two independent samples Student’s t test was performed; for categorical variables, Chi-square test was used for comparisons between groups. A two-tailed *p*-value of < 0.05 was considered statistically significant.

## Results

### Clinical and CT morphological features

Among the 608 patients with LADC, 272 patients were women and 336 were men with an average age of 61.7 ± 10.4 (range: 30–85) years. For clinical staging, 190 patients (31.2%) were in stages I-II and 418 (68.8%) in stages III-IV. Compared to patients without EGFR-mutation, female, nonsmokers, tumor size<3cm, subsolid density, air bronchogram, air space, spiculation, pleural retraction, vascular convergence sign, and multiple pulmonary metastases were more common in those with EGFR-mutation (all *p <*0.05). Compared to patients with non 19del-mutation, younger age, female, nonsmokers, tumor size <3cm, subsolid density, air bronchogram, pleural retraction, and vascular convergence sign were more frequent in those with 19del-mutation (all *p <*0.05). Compared to patients with non L858R-mutation, female, nonsmokers, pleural retraction, vascular convergence sign, and without necrosis were more common in those with L858R-mutation (all *p <*0.05) (**Table 1**). The clinical data and CT morphological features of patients in training and validation cohorts for model 1 to 3 were shown in **Tables 2**–**4**, respectively. No significant differences were observed in clinical and CT morphological features between both cohorts in each model (all *p* > 0.05).

**Table 1:** Clinical and CT morphological features of patients with LADC between different EGFR-mutation groups.

			EGFR-mutation statuses			
Clinical and CT features	EGFR (+) vs EGFR (-) (307 vs 301)	*p*-value	19del vs Non-19del (114 vs 494)	*p*-value	L858R vs Non-L858R (155 vs 453)	*p*-value
Age (years)	61.1 ± 10.8 vs 62.2 ± 10.0	0.224^a^	60.9 ± 11.9 vs 61.8 ± 10.1	0.013^a^	60.9 ± 10.1 vs 61.9 ± 10.6	0.317^a^
Sex (female)	180 (58.6%) vs 92 (30.6%)	<0.001^b^	72 (63.2%) vs 200 (40.5%)	<0.001^b^	86 (55.5%) vs 186 (41.1%)	0.002^b^
Non-smokers	213 (69.4%) vs 122 (40.5%)	<0.001^b^	81 (71.1%) vs 254 (51.4%)	<0.001^b^	107 (69.0%) vs 228 (50.3%)	<0.001^b^
Clinical stage (I ~ II)	107 (34.9%) vs 83 (27.6%)	0.053^b^	35 (30.7%) vs 155 (31.4)	0.889^b^	55 (35.5%) vs 135 (29.8%)	0.188^b^
Location (peripheral)	243 (79.2%) vs 221 (73.4%)	0.097^b^	93 (81.6) vs 371 (75.1%)	0.143^b^	115 (74.2%) vs 349 (77.0%)	0.472^b^
Tumor size≥3cm	161 (52.4%) vs 193 (64.1%)	0.004^b^	54 (47.4%) vs 300 (60.7%)	0.009^b^	92 (59.4%) vs 262 (57.8%)	0.741^b^
Subsolid density (presence)	59 (19.2%) vs 24 (8.0%)	<0.001^b^	28 (24.6%) vs 55 (11.1%)	<0.001^b^	26 (16.8%) vs 57 (12.6%)	0.19^b^
Spiculation (presence)	89 (29.0%) vs 65 (21.6%)	0.036^b^	37 (32.5%) vs 117 (23.7%)	0.052^b^	37 (23.9%) vs 117 (25.8%)	0.629^b^
lobulation (presence)	291 (94.8%) vs 275 (91.4%)	0.096^b^	108 (94.7%) vs 458 (92.7%)	0.442^b^	145 (93.5%) vs 421 (92.9%)	0.795^b^
Air bronchogram (presence)	64 (20.8%) vs 28 (9.3%)	<0.001^b^	30 (26.3%) vs 62 (12.6%)	<0.001^b^	27 (17.4%) vs 65 (14.3%)	0.357^b^
Air space (presence)	60 (19.5%) vs 55 (18.3%)	0.689^b^	23 (20.2%) vs 92 (18.6%)	0.703^b^	29 (18.7%) vs 86 (19.0%)	0.94^b^
Necrosis (presence)	28 (9.1%) vs 65 (21.6%)	<0.001^b^	7 (9.6%) vs 82 (16.6%)	0.063^b^	14 (9.0%) vs 79 (17.4%)	0.012^b^
Calcification (presence)	15 (4.9%) vs 14 (4.7%)	0.892^b^	7 (6.1%) vs 22 (4.5%)	0.446^b^	7 (4.5%) vs 22 (4.9%)	0.864^b^
Vascular convergence sign (presence)	107 (34.9%) vs37 (12.3)	<0.001^b^	39 (34.2%) vs 105 (21.3%)	0.003^b^	49 (31.6%) vs 95 (21.0%)	0.007^b^
Pleural retraction sign (presence)	197 (64.2%) vs 116 (38.5%)	<0.001^b^	73 (64.0%) vs 240 (48.6%)	0.003^b^	102 (65.8%) vs 211 (46.6%)	<0.001^b^
Pleural effusion (presence)	67 (21.8%) vs 89 (29.6%)	0.029^b^	34 (29.8) vs 122 (24.7%)	0.258^b^	30 (19.4%) vs 126 (27.8%)	0.037^b^
Lymphatic metastasis (presence)	166 (54.1%) vs 200 (66.4%)	0.002^b^	65 (57.0%) vs301 (60.9%)	0.442^b^	84 (54.2%) vs 282 (62.3%)	0.077^b^
Multiple pulmonary metastases (n≥10)	53 (17.3%) vs 28 (9.3%)	0.004^b^	21 (18.4%) vs 60 (12.1%)	0.076^b^	27 (17.4%) vs 54 (11.9)	0.082^b^

^a^Two independent samples Student’s t test.

^b^Chi-squared test.

**Table 2:** Clinical and CT morphological features of patients with LADC in training and validation cohorts of model 1.

Clinical and CT features	Training cohort (n=487)		Validation cohort (n=121)		*p*-value
	EGFR (+) (n=246)	EGFR (-) (n=241)	EGFR (+) (n=61)	EGFR (-) (n=60)	
Age (years)	61.5 ± 11.6	62.5 ± 10.0	61.1 ± 10.6	61.0 ± 10.0	0.568^a^
Female	31 (50.8%)	77 (32.0%)	149 (60.6%)	15 (25.0%)	0.175^b^
Non-smokers	44 (72.1%)	100 (41.5%)	169 (68.7%)	22 (36.7%)	0.972^b^
Clinical stages (I ~ II)	24 (39.3%)	64 (26.6%)	83 (33.7%)	19 (31.7%)	0.491^b^
Location (peripheral)	50 (82.0%)	174 (72.2%)	193 (78.5%)	47 (78.3%)	0.321^b^
Tumor size ≥3cm	29 (47.5%)	152 (63.1%)	132 (53.7%)	41 (68.3%)	1.000^b^
Spiculation	70 (28.5%)	57 (23.7%)	19 (31.1%)	8 (13.3%)	0.462^b^
Lobulation	234 (95.1%)	223 (92.5%)	57 (93.4%)	52 (86.7%)	0.208^b^
Subsolid density	48 (19.9%)	21 (8.7%)	11 (18.0%)	3 (5.0%)	0.550^b^
Air bronchogram	52 (21.1%)	24 (10.0%)	12 (19.7%)	4 (6.7%)	0.608^b^
Air space	46 (18.7%)	48 (19.9%)	14 (23.0%)	7 (11.7%)	0.719^b^
Necrosis	21 (8.5%)	52 (21.6%)	7 (11.5%)	13 (21.7%)	0.780^b^
Calcification	15 (6.1%)	12 (5.0%)	0 (0.0%)	2 (3.3%)	0.119^b^
Vascular convergence sign	83 (33.7%)	33 (13.7%)	24 (39.3%)	4 (6.7%)	0.970^b^
Pleural retraction	155 (63.0%)	96 (39.8%)	42 (68.9%)	20 (33.3%)	1.000^b^
Pleural effusion	56 (22.8%)	72 (29.9%)	11 (18.0%)	17 (28.3%)	0.554^b^
Lymphatic metastasis	134 (54.5%)	160 (66.4%)	32 (52.5%)	40 (66.7%)	0.944^b^
Multiple pulmonary metastases	42 (17.1%)	24 (10.0%)	11 (18.0%)	4 (6.7%)	0.853^b^

a Two independent samples Student’s t test.

b Chi-squared test.

**Table 3:** Clinical and CT morphological features of patients with LADC in training and validation cohorts of model 2.

Clinical and CT features	Training cohort (n=486)		Validation cohort (n=122)		*p*-value
	19del (n=91)	Non-19del (n=395)	19del (n=23)	Non-19del (n=99)	
Age (years)	61.0 ± 11.5	61.5 ± 9.8	60.7 ± 13.2	63.1 ± 10.8	0.987^a^
Female	58 (63.7%)	160 (40.5%)	14 (60.9%)	40 (40.4%)	0.643^b^
Non-smokers	67 (73.6%)	198 (50.1%)	14 (60.9%)	56 (56.6%)	0.320^b^
Clinical stages (I~II)	24 (26.4%)	118 (29.9%)	11 (47.8%)	37 (37.4%)	0.925^b^
Location (peripheral)	73 (80.2%)	297 (75.2%)	20 (87.0%)	74 (74.7%)	0.612^b^
Tumor size ≥3cm	45 (49.5%)	235 (59.5%)	9 (39.1%)	65 (65.7%)	0.077^b^
Spiculation	29 (31.9%)	86 (21.8%)	8 (34.8%)	31 (31.3%)	0.077^b^
Lobulation	86 (94.5%)	368 (93.2%)	22 (95.7%)	90 (90.9%)	0.668^b^
Subsolid density	21 (23.1%)	40 (10.1%)	8 (34.8%)	14 (14.1%)	0.153^b^
Air bronchogram	22 (24.2%)	49 (12.4%)	9 (39.1%)	12 (12.1%)	0.564^b^
Air space	16 (17.6%)	76 (19.2%)	7 (30.4%)	16 (16.2%)	1.000^b^
Necrosis	8 (8.8%)	68 (17.2%)	3 (13.0%)	14 (14.1%)	0.744^b^
Calcification	5 (5.5%)	18 (4.6%)	2 (8.7%)	4 (4.0%)	1.000^b^
Vascular convergence sign	29 (31.9%)	82 (20.8%)	9 (39.1%)	24 (24.2%)	0.390^b^
Pleural retraction	58 (63.7%)	187 (47.3%)	16 (69.6%)	52 (52.5%)	0.342^b^
Pleural effusion	27 (29.7%)	98 (24.8%)	6 (26.1%)	25 (25.3%)	1.000^b^
Lymphatic metastasis	53 (58.2%)	246 (62.3%)	10 (43.5%)	57 (57.6%)	0.219^b^
Multiple pulmonary metastases	17 (18.7%)	46 (11.6%)	4 (17.4%)	14 (14.1%)	0.710^b^

a Two independent samples Student’s t test.

b Chi-squared test.

**Table 4:** Clinical and CT morphological features of patients with LADC in training and validation cohorts of model 3.

Clinical and CT features	Training cohort (n=486)		Validation cohort (n=122)		*p*-value
	L858R (n=122)	Non-L858R (n=364)	L858R (n=33)	Non-L858R (n=89)	
Age (years)	60.8 ± 9.7	61.7 ± 10.6	62.8 ± 9.6	62.9 ± 10.1	0.568^a^
Female	66 (54.1%)	153 (42.0%)	20 (60.6%)	33 (37.1%)	0.987^b^
Non-smokers	83 (68.0%)	181 (49.7%)	24 (72.7%)	47 (52.8%)	0.504^b^
Clinical stages (I ~ II)	41 (33.6%)	107 (29.4%)	14 (42.4%)	28 (31.5%)	0.604^b^
Location (peripheral)	88 (72.1%)	283 (77.7%)	27 (81.8%)	66 (74.2%)	1.000^b^
Tumor size≥3cm	70 (57.4%)	208 (57.1%)	22 (66.7%)	54 (60.7%)	0.477^b^
Spiculation	31 (25.4%)	93 (25.5%)	6 (18.2%)	25 (28.1%)	0.926^b^
Lobulation	115 (94.3%)	336 (92.3%)	30 (90.9%)	85 (95.5%)	0.711^b^
Subsolid density	21 (17.2%)	45 (12.4%)	6 (18.2%)	11 (12.4%)	1.000^b^
Air bronchogram	25 (20.5%)	49 (13.5%)	3 (9.1%)	15 (16.9%)	1.000^b^
Air space	24 (19.7%)	70 (19.2%)	6 (18.2%)	15 (6.9%)	0.684^b^
Necrosis	12 (9.8%)	68 (18.7%)	1 (3.0%)	12 (13.5%)	0.147^b^
Calcification	7 (5.7%)	16 (4.4%)	0 (0.0%)	6 (6.7%)	1.000^b^
Vascular convergence sign	36 (29.5%)	80 (22.0%)	14 (42.4%)	14 (15.7%)	0.925^b^
Pleural retraction	78 (63.9%)	174 (47.8%)	25 (75.8%)	36 (40.4%)	0.791^b^
Pleural effusion	27 (22.1%)	100 (27.5%)	3 (9.1%)	26 (29.2%)	0.676^b^
Lymphatic metastasis	67 (54.9%)	228 (62.6%)	17 (51.5%)	54 (60.7%)	0.688^b^
Multiple pulmonary metastases	19 (15.6%)	43 (11.8%)	7 (21.2%)	12 (13.5%)	0.503^b^

a Two independent samples Student’s t test.

b Chi-squared test.

### Establishment and validation of prediction models

**Figure 3:**
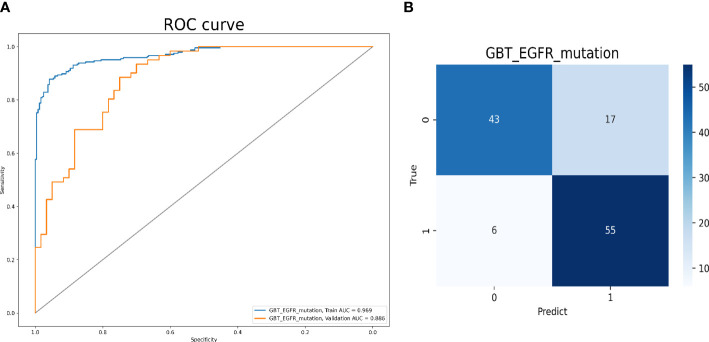
Model 1 validation. **(A)** The ROC curves for model 1 to identify positive and negative *EGFR* mutation cases. **(B)** The confusion matrix for model 1.

Model 1 was built with 137 radiomics features, including 25 first-order features, two shape 2D features, 110 advanced textural features (30 GLCM, 35 GLSZM, 20 GLRZM, 13 NGTDM, and 12 GLDM) as well as 18 clinical and CT morphological features. The AUCs for predicting *EGFR*-mutation positive cases were 0.969 and 0.886 in the training and validation cohorts, respectively. The accuracy, sensitivity, and specificity of the validation cohort were 0.810, 0.902, and 0.717, respectively (**Figure 3**).

**Figure 4:**
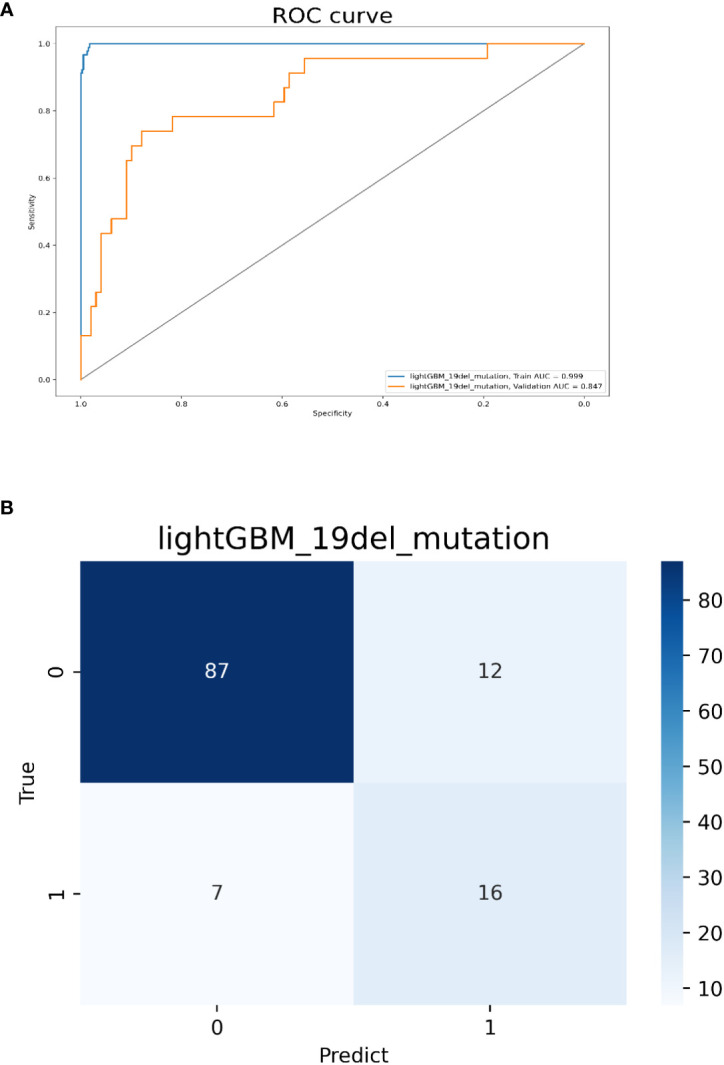
Model 2 validation. **(A)** The ROC curves for model 2 to identify 19del mutations. **(B)** The confusion matrix for model 2.

Model 2 was built with all 202 features and the top 10 ones sorted by their importance scores included 5 radiomics features (5 advanced texture features), 3 clinical features (female, no-smokers, younger age), and 2 CT morphological features (tumor size<3cm, subsolid density). The AUC values for predicting 19del-mutation were 0.999 and 0.847 in the training and validation cohorts, respectively. The accuracy, sensitivity, and specificity in the validation cohort were 0.852, 0.739, and 0.879, respectively (**Figure 4**). The radiomics, clinical, and CT morphology features used for establishing model 2 and their importance scores are detailed in **Supplementary Table 1**.

**Figure 5:**
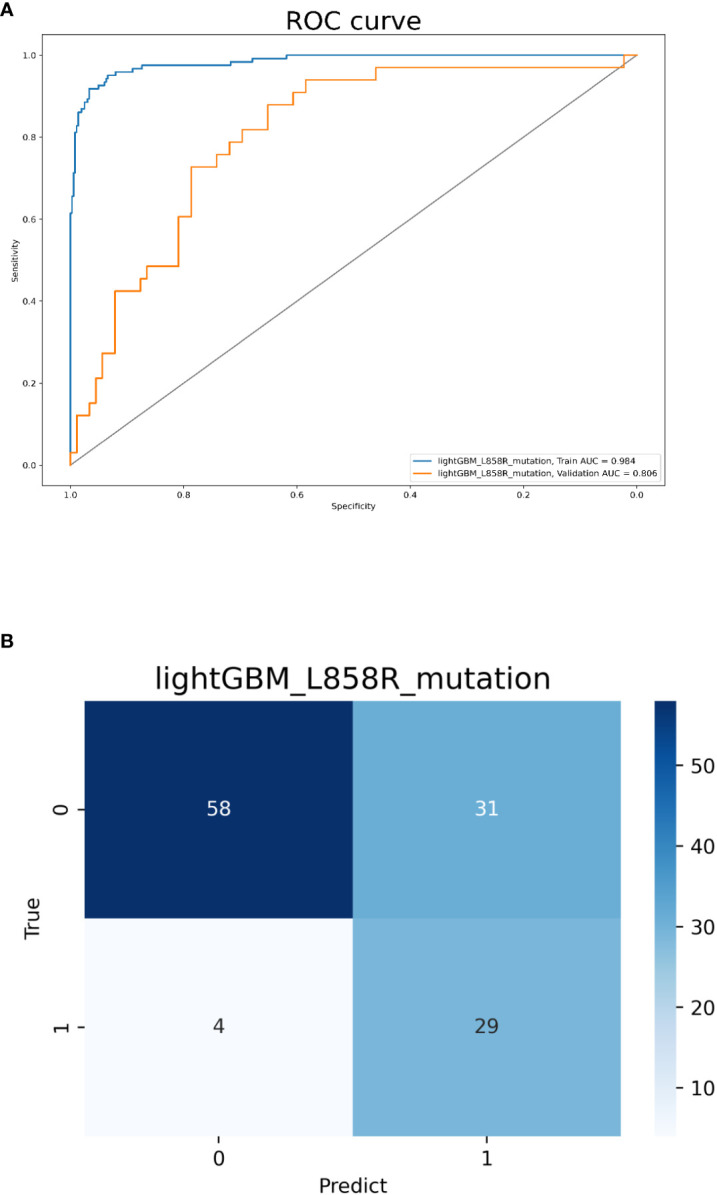
Model 3 validation. **(A)** The ROC curves for model 3 to identify L858R mutations. **(B)** The confusion matrix for model 3.

Model 3 was built with all 358 features and the top 10 ones sorted by their importance scores included 6 radiomics features (2 first-order features, 4 advanced texture features), 2 clinical (no-smokers, female) and 2 CT morphological features (pleural retraction, vascular convergence sign). The AUCs for predicting L858R-mutation were 0.984 and 0.806 in the training and validation cohorts, respectively. The accuracy, sensitivity, and specificity in the validation cohort were 0.713, 0.879, and 0.652, respectively (**Figure 5**). The radiomics, clinical, and CT morphology features used for establishing model 3 and their importance scores are detailed in **Supplementary Table 2**.

## Discussion

With the development of targeted therapy technology, the prognosis of LADC patients has greatly improved. The noninvasive and quantified prediction of *EGFR*-mutation status would provide great value to clinicians in selecting the best LADC therapy, which could further extend PFS. Therefore, we established and validated three combined models that incorporated radiomics signatures, clinical indicators, and CT morphological features to better predict the mutation status of *EGFR*, focusing on the prediction of 19del and L858R mutations.

First, we built model 1 to identify positive and negative *EGFR*-mutation in LADC. In this model, 137 radiomics features as well as 18 clinical and CT morphological features were included with AUCs of 0.965 and 0.886 in the training and validation cohorts, respectively. Jia et al. (22) reported that random forest model features combined with sex and smoking history had the potential to predict *EGFR*-mutation status of LADC with an AUC of 0.828. Zhang et al. (18) demonstrated that a Squeeze-and-Excitation Convolutional Neural Network (SE CNN) can recognize *EGFR*-mutation status of LADC with AUCs of 0.910 and 0.841 for the internal and external test cohorts, respectively. Paralleled with previous research, our research has several advantages. First, our model was based on the machine learning model of the classic general algorithm, which can be applied to a range of scenarios and withstand verification. Since the universality of the classical algorithm is well established, it has potential to be clinically implemented. Second, our models performed better than those in other studies. We only used the biggest level to establish the model instead of multi-layer and multi-sequence labeling, which saves time and reduces the clinician workload.

Previous studies indicated that LADC patients with different *EGFR*-mutation subtypes may exhibit different prognoses to targeted therapy (9, 11). Investigators have elucidated the mechanism(s) underlying the different sensitivities to EGFR-TKI treatment between patients with 19del and L858R mutations. Zhu et al. (23) suggested that G1 arrest levels were higher in cells with 19del-mutation than those with L858R-mutation after treatment with gefitinib. Sordella et al. (24) found that different *EGFR*-mutation subtypes may alter autophosphorylation and downstream signaling pathways. An accurate assessment of *EGFR*-mutation subtypes of tumors may help select the optimal treatment strategy, thereby improving the quality of life and prolonging survival of patients with LADC. Therefore, we further established models 2 and 3 to differentiate 19del and L858R mutation statuses, respectively.

Both models exhibited good performances and found some important features to identify EGFR-mutation subtypes. For clinical features, our results showed that female and no-smokers were correlated to 19del-mutation and L858R-mutation, which is consistent with previous studies (7, 9). Moreover, we found 19del-mutation was more common in younger patients. For CT morphological features, 19del-mutation were more frequent in tumors with size<3cm and subsolid density, while L858R-mutation were more frequent in those with pleural retraction and vascular convergence sign, which is similar to the results of other scholars (8, 25). For radiomics features, we found that 5 advanced texture features were associated with 19del-mutation, whereas 2 first-order features and 4 advanced texture features were related to L858R-mutation. Generally, advanced texture features are used to describe the surface properties of the scene corresponding to the image or image area, while first-order features are used to describe the distribution of voxel intensities within the ROI using common and basic metrics (26, 27), indicating that tumors with 19del and L858R mutations may correlate to aforementioned characteristics.

Actually, some studies have reported the clinical, CT morphological, and radiomics features can be used to predict predominant *EGFR*-mutation subtypes. Shi et al. (7) used clinical and CT morphological characteristics to identify 19 del and L858R mutations of LADC, and the AUC for the model was 0.793. Li et al. (28) used combined model incorporating clinical and radiomics features can predict the common subtypes of *EGFR*-mutation in LADC, and the AUC for the model was 0.775. Additionally, Song et al. (29) applied a deep learning model to classify *EGFR*-mutation subtypes, and they confirmed that imaging phenotypes of the two mutation-subtype tumors (19del, L858R) were different with AUCs of models to identify the two subtypes were 0.78 and 0.79, respectively. However, those studies did not achieve a satisfactory effectiveness and may be difficult to apply in the clinic. Compared to previous reports, our models had a superior performance when incorporating clinical characteristics, CT morphological features, and radiomics signatures. The two combined models could be an auxiliary tool to predict *EGFR*-mutation subtypes in LADC patients.

Recently, some studies have indicated that 18F-FDG-PET/CT-based and MRI-based radiomics models were promising alternatives to predict the EGFR-mutation subtypes in LADC patients. Liu et al. (30) showed that 18F-FDG PET/CT-based radiomics features may be valuable for identifying 19del and L858R mutations in LADC; the AUC values were 0.77 and 0.92, respectively. However, the increased radiation doses and high examination costs of PET/CT have restricted its clinical application (31). Rao et al. (32) demonstrated that a MRI-based radiomics nomogram can be adopted to differentiate exons 19 and 21 in *EGFR* mutation by analyzing spinal bone metastases in patients with LADC; the AUC values for these models were 0.87 and 0.86, respectively. However, compared with chest CT scan, MRI fails due to a weak lung signal, increased inspection time, obvious respiratory artifacts, and poor image quality (33).

This study has several limitations. First, this work was performed in a single center and lacked external verification. We are preparing to conduct a multicenter study to verify the reliability and general applicability of this model. Second, incomplete patient records mean that some clinical parameters and serum biomarkers were not included in this study. Finally, this study only predicted *EGFR*-mutation in LADC and excluded other lung cancer gene mutations. Therefore, further studies are needed.

In conclusion, combined models that incorporate radiomics signatures, clinical, and CT morphological features have the potential to identify *EGFR*-mutation subtypes, which may contribute to individualizing LADC patient therapy.

## Data availability statement

The original contributions presented in the study are included in the article/**Supplementary Material**. Further inquiries can be directed to the corresponding author.

## Ethics statement

The studies involving human participants were reviewed and approved by the ethical committee of The First Affiliated Hospital of Chongqing Medical University. The requirement for patient informed consent was waived due to the retrospective nature of study.

## Author contributions

J-WH and T-YL have contributed equally to this work and share first authorship. All authors contributed to the article and approved the submitted version.

## Funding

This study was supported by Chongqing Health and Family Planning Commission Foundation (2022MSXM147) of China and Chongqing Health Commission (Chongqing Talent Program-Innovation Leading Talent Research Project) (CQYC20210303348) and Chongqing Science and Technology Bureau (cstc2022ycjh-bgzxm0230).

## Conflict of interest

Authors LD, W-dC and R-zY are employed by Infervision.

The remaining authors declare that the research was conducted in the absence of any commercial or financial relationships that could be construed as a potential conflict of interest.

## Publisher’s note

All claims expressed in this article are solely those of the authors and do not necessarily represent those of their affiliated organizations, or those of the publisher, the editors and the reviewers. Any product that may be evaluated in this article, or claim that may be made by its manufacturer, is not guaranteed or endorsed by the publisher.

## Abbreviations

AUC, area under the curve; CT, computed tomography; EGFR, epidermal growth factor receptor; LADC, lung adenocarcinoma; PFS, progression-free survival; ROC, receiver operating characteristic; ROI, region of interest; TKI, tyrosine kinase inhibitor.
