# Supplementary material for: Using combined CT-clinical radiomics models to identify epidermal growth factor receptor mutation subtypes in lung adenocarcinoma
Source: Front Oncol. 2022 Aug 18;12:846589. doi: 10.3389/fonc.2022.846589 (PMC9434115; doi:10.3389/fonc.2022.846589)
Supplement: Supplementary file 1 [file Table_1.docx]

**Supplementary Table 1** The radiomics, clinical, and CT morphology features and their importance scores for model 2

| **Features** | **importance** | **stddev** | **p_value** | **n** | **p99_high** | **p99_low** |
| --- | --- | --- | --- | --- | --- | --- |
| sex | 0.068364807 | 0.04407161 | 0.05754846 | 3 | 0.320900006 | -0.18417039 |
| smoking history | 0.030595813 | 0.0026834 | 0.00127711 | 3 | 0.045971991 | 0.015219636 |
| exponential_glrlm_ShortRunLowGrayLevelEmphasis | 0.027667984 | 0.00847049 | 0.01492516 | 3 | 0.076204821 | -0.02086885 |
| maximum diameter | 0.024593764 | 0.00706784 | 0.01322135 | 3 | 0.065093284 | -0.01590576 |
| gradient_glcm_Imc1 | 0.007319573 | 0.00126779 | 0.00492623 | 3 | 0.014584134 | 5.50E-05 |
| subsolid | 0.00702679 | 0.00201255 | 0.0131355 | 3 | 0.018558923 | -0.00450534 |
| age | 0.006148441 | 0.00343006 | 0.04498104 | 3 | 0.025803073 | -0.01350619 |
| gradient_glcm_MCC | 0.006002049 | 0.00225367 | 0.02196137 | 3 | 0.018915816 | -0.00691172 |
| original_glrlm_LongRunHighGrayLevelEmphasis | 0.003952569 | 0.00263505 | 0.06084497 | 3 | 0.019051676 | -0.01114654 |
| gradient_glcm_Correlation | 0.003659786 | 0.00154233 | 0.02720658 | 3 | 0.012497506 | -0.00517793 |
| wavelet-LL_gldm_LargeDependenceHighGrayLevelEmphasis | 0.003367003 | 0.0013417 | 0.02453352 | 3 | 0.011055092 | -0.00432109 |
| square_glszm_HighGrayLevelZoneEmphasis | 0.002927829 | 0.00050712 | 0.00492623 | 3 | 0.005833653 | 2.20E-05 |
| gradient_firstorder_90Percentile | 0.002927829 | 0.00101423 | 0.01887478 | 3 | 0.008739478 | -0.00288382 |
| exponential_glrlm_ShortRunHighGrayLevelEmphasis | 0.002781438 | 0.00902536 | 0.32343715 | 3 | 0.054497765 | -0.04893489 |
| original_gldm_LargeDependenceHighGrayLevelEmphasis | 0.002635046 | 0.00131752 | 0.03708995 | 3 | 0.010184599 | -0.00491451 |
| spiculation | 0.002635046 | 0.00418946 | 0.19487142 | 3 | 0.026641095 | -0.021371 |
| original_shape2D_Elongation | 0.002488655 | 0.00166269 | 0.06106189 | 3 | 0.012016037 | -0.00703873 |
| peural retraction | 0.002488655 | 0.00651894 | 0.28822672 | 3 | 0.039842911 | -0.0348656 |
| wavelet-HH_glszm_LowGrayLevelZoneEmphasis | 0.002488655 | 0.0013417 | 0.04237538 | 3 | 0.010176743 | -0.00519943 |
| original_shape2D_Sphericity | 0.002342263 | 0.00461307 | 0.23596066 | 3 | 0.028775684 | -0.02409116 |
| logarithm_gldm_DependenceVariance | 0.002195872 | 6.41E-17 | 1.42E-28 | 3 | 0.002195872 | 0.002195872 |
| gradient_glrlm_ShortRunHighGrayLevelEmphasis | 0.002195872 | 6.41E-17 | 1.42E-28 | 3 | 0.002195872 | 0.002195872 |
| gradient_glrlm_GrayLevelVariance | 0.00204948 | 0.00154233 | 0.07399357 | 3 | 0.0108872 | -0.00678824 |
| original_glcm_Autocorrelation | 0.00204948 | 0.00067085 | 0.01695411 | 3 | 0.005893525 | -0.00179456 |
| gradient_glszm_SmallAreaHighGrayLevelEmphasis | 0.001903089 | 0.00110523 | 0.04821877 | 3 | 0.008236186 | -0.00443001 |
| wavelet-LL_glrlm_LongRunHighGrayLevelEmphasis | 0.001756697 | 0.00158347 | 0.09730637 | 3 | 0.010830131 | -0.00731674 |
| original_glcm_JointAverage | 0.001756697 | 0.00274264 | 0.1913933 | 3 | 0.017472346 | -0.01395895 |
| wavelet-LH_glcm_Idmn | 0.001756697 | 0.00043917 | 0.01010205 | 3 | 0.004273215 | -0.00075982 |
| square_ngtdm_Busyness | 0.001756697 | 0.00087835 | 0.03708995 | 3 | 0.006789733 | -0.00327634 |
| gradient_glcm_SumSquares | 0.001610306 | 0.00067085 | 0.02663537 | 3 | 0.00545435 | -0.00223374 |
| square_glszm_SmallAreaHighGrayLevelEmphasis | 0.001463915 | 0.00225367 | 0.1887136 | 3 | 0.014377681 | -0.01144985 |
| squareroot_glszm_HighGrayLevelZoneEmphasis | 0.001317523 | 0.00116195 | 0.09424866 | 3 | 0.007975603 | -0.00534056 |
| wavelet-LL_firstorder_Energy | 0.001317523 | 0.00043917 | 0.01754936 | 3 | 0.003834041 | -0.001199 |
| square_gldm_LargeDependenceHighGrayLevelEmphasis | 0.001317523 | 0.00076067 | 0.04773298 | 3 | 0.00567626 | -0.00304121 |
| square_glszm_SizeZoneNonUniformity | 0.001317523 | 0.00043917 | 0.01754936 | 3 | 0.003834041 | -0.001199 |
| wavelet-HH_glrlm_LongRunHighGrayLevelEmphasis | 0.001317523 | 0.00087835 | 0.06084497 | 3 | 0.006350559 | -0.00371551 |
| wavelet-LL_glrlm_GrayLevelVariance | 0.001171132 | 0.00067085 | 0.04708919 | 3 | 0.005015176 | -0.00267291 |
| wavelet-LH_firstorder_Kurtosis | 0.001171132 | 0.00025356 | 0.00763404 | 3 | 0.002624044 | -0.00028178 |
| wavelet-HH_firstorder_Energy | 0.001171132 | 0.00050712 | 0.02859548 | 3 | 0.004076956 | -0.00173469 |
| wavelet-HH_gldm_SmallDependenceLowGrayLevelEmphasis | 0.001171132 | 0.00166269 | 0.17340137 | 3 | 0.010698514 | -0.00835625 |
| wavelet-LH_firstorder_Maximum | 0.001171132 | 0.00067085 | 0.04708919 | 3 | 0.005015176 | -0.00267291 |
| wavelet-HH_glszm_SizeZoneNonUniformity | 0.001171132 | 0.00067085 | 0.04708919 | 3 | 0.005015176 | -0.00267291 |
| wavelet-HH_glszm_ZonePercentage | 0.001171132 | 0.00050712 | 0.02859548 | 3 | 0.004076956 | -0.00173469 |
| square_glszm_GrayLevelNonUniformity | 0.001171132 | 0.0017749 | 0.18573032 | 3 | 0.011341517 | -0.00899925 |
| logarithm_glszm_GrayLevelVariance | 0.001171132 | 0.0013417 | 0.13485163 | 3 | 0.00885922 | -0.00651696 |
| original_glcm_InverseVariance | 0.00102474 | 0.00110523 | 0.12476061 | 3 | 0.007357838 | -0.00530836 |
| logarithm_glrlm_LongRunLowGrayLevelEmphasis | 0.00102474 | 0.00025356 | 0.00990197 | 3 | 0.002477652 | -0.00042817 |
| square_gldm_SmallDependenceLowGrayLevelEmphasis | 0.00102474 | 0.00050712 | 0.03641368 | 3 | 0.003930565 | -0.00188108 |
| wavelet-HL_firstorder_InterquartileRange | 0.00102474 | 0.0013417 | 0.15843497 | 3 | 0.008712829 | -0.00666335 |
| logarithm_glszm_HighGrayLevelZoneEmphasis | 0.00102474 | 0.00050712 | 0.03641368 | 3 | 0.003930565 | -0.00188108 |
| square_gldm_DependenceVariance | 0.00102474 | 0.00141175 | 0.1677947 | 3 | 0.009114213 | -0.00706473 |
| wavelet-LL_glcm_JointAverage | 0.000878349 | 0.00116195 | 0.16031689 | 3 | 0.007536429 | -0.00577973 |
| square_glrlm_HighGrayLevelRunEmphasis | 0.000878349 | 0.00043917 | 0.03708995 | 3 | 0.003394866 | -0.00163817 |
| wavelet-HH_ngtdm_Contrast | 0.000878349 | 0.00043917 | 0.03708995 | 3 | 0.003394866 | -0.00163817 |
| square_gldm_SmallDependenceHighGrayLevelEmphasis | 0.000878349 | 0.00076067 | 0.09175171 | 3 | 0.005237085 | -0.00348039 |
| wavelet-LL_glcm_Imc1 | 0.000878349 | 0.00043917 | 0.03708995 | 3 | 0.003394866 | -0.00163817 |
| wavelet-HL_glcm_ClusterShade | 0.000878349 | 0.00043917 | 0.03708995 | 3 | 0.003394866 | -0.00163817 |
| gradient_glszm_HighGrayLevelZoneEmphasis | 0.000878349 | 0.00087835 | 0.11270167 | 3 | 0.005911384 | -0.00415469 |
| wavelet-LH_glszm_SmallAreaLowGrayLevelEmphasis | 0.000878349 | 0.00043917 | 0.03708995 | 3 | 0.003394866 | -0.00163817 |
| wavelet-HH_firstorder_Median | 0.000878349 | 0.00043917 | 0.03708995 | 3 | 0.003394866 | -0.00163817 |
| square_glszm_SmallAreaLowGrayLevelEmphasis | 0.000878349 | 0.00076067 | 0.09175171 | 3 | 0.005237085 | -0.00348039 |
| clinical stage | 0.000878349 | 0.00191432 | 0.25505103 | 3 | 0.011847595 | -0.0100909 |
| squareroot_firstorder_Range | 0.000878349 | 0.00087835 | 0.11270167 | 3 | 0.005911384 | -0.00415469 |
| gradient_glcm_Idm | 0.000878349 | 0.00087835 | 0.11270167 | 3 | 0.005911384 | -0.00415469 |
| original_gldm_DependenceVariance | 0.000878349 | 0.00043917 | 0.03708995 | 3 | 0.003394866 | -0.00163817 |
| wavelet-HL_glszm_SmallAreaHighGrayLevelEmphasis | 0.000878349 | 0.00116195 | 0.16031689 | 3 | 0.007536429 | -0.00577973 |
| air bronchogram | 0.000878349 | 0.00152135 | 0.21132487 | 3 | 0.009595822 | -0.00783912 |
| wavelet-LL_glcm_ClusterShade | 0.000878349 | 0 | 0.5 | 3 | 0.000878349 | 0.000878349 |
| wavelet-LH_gldm_DependenceNonUniformityNormalized | 0.000878349 | 0.00158347 | 0.21902426 | 3 | 0.009951782 | -0.00819509 |
| original_glrlm_LongRunEmphasis | 0.000878349 | 0.00152135 | 0.21132487 | 3 | 0.009595822 | -0.00783912 |
| wavelet-HL_glrlm_RunLengthNonUniformityNormalized | 0.000731957 | 0.00025356 | 0.01887478 | 3 | 0.002184869 | -0.00072096 |
| logarithm_glcm_Imc2 | 0.000731957 | 0.00025356 | 0.01887478 | 3 | 0.002184869 | -0.00072096 |
| wavelet-HH_firstorder_Minimum | 0.000731957 | 0.00050712 | 0.06480586 | 3 | 0.003637782 | -0.00217387 |
| gradient_gldm_DependenceNonUniformityNormalized | 0.000731957 | 0.00025356 | 0.01887478 | 3 | 0.002184869 | -0.00072096 |
| wavelet-LH_gldm_SmallDependenceHighGrayLevelEmphasis | 0.000731957 | 0.00091421 | 0.14992998 | 3 | 0.005970507 | -0.00450659 |
| logarithm_gldm_DependenceNonUniformityNormalized | 0.000731957 | 0.00025356 | 0.01887478 | 3 | 0.002184869 | -0.00072096 |
| logarithm_ngtdm_Busyness | 0.000731957 | 0.00067085 | 0.09967962 | 3 | 0.004576002 | -0.00311209 |
| square_glszm_SizeZoneNonUniformityNormalized | 0.000731957 | 0.00101423 | 0.16886691 | 3 | 0.006543606 | -0.00507969 |
| original_glszm_LargeAreaHighGrayLevelEmphasis | 0.000731957 | 0.0017749 | 0.27458259 | 3 | 0.010902343 | -0.00943843 |
| square_glcm_Imc1 | 0.000731957 | 0.00198035 | 0.29380348 | 3 | 0.012079564 | -0.01061565 |
| wavelet-LH_firstorder_RootMeanSquared | 0.000731957 | 0.00025356 | 0.01887478 | 3 | 0.002184869 | -0.00072096 |
| wavelet-LL_firstorder_Uniformity | 0.000731957 | 0.00067085 | 0.09967962 | 3 | 0.004576002 | -0.00311209 |
| squareroot_glcm_MCC | 0.000731957 | 0.00182843 | 0.27988727 | 3 | 0.011209056 | -0.00974514 |
| wavelet-LL_ngtdm_Complexity | 0.000731957 | 0.00025356 | 0.01887478 | 3 | 0.002184869 | -0.00072096 |
| squareroot_glcm_DifferenceAverage | 0.000585566 | 0.00025356 | 0.02859548 | 3 | 0.002038478 | -0.00086735 |
| wavelet-HH_glszm_SmallAreaLowGrayLevelEmphasis | 0.000585566 | 0.00067085 | 0.13485163 | 3 | 0.00442961 | -0.00325848 |
| wavelet-HH_glcm_ClusterProminence | 0.000585566 | 0.00025356 | 0.02859548 | 3 | 0.002038478 | -0.00086735 |
| square_glrlm_RunPercentage | 0.000585566 | 0.00025356 | 0.02859548 | 3 | 0.002038478 | -0.00086735 |
| wavelet-LL_glrlm_LongRunEmphasis | 0.000585566 | 0.00091421 | 0.1913933 | 3 | 0.005824115 | -0.00465298 |
| logarithm_glszm_LowGrayLevelZoneEmphasis | 0.000585566 | 0.00050712 | 0.09175171 | 3 | 0.00349139 | -0.00232026 |
| squareroot_firstorder_RootMeanSquared | 0.000585566 | 0.00141175 | 0.27354459 | 3 | 0.008675039 | -0.00750391 |
| wavelet-HH_firstorder_90Percentile | 0.000585566 | 0.00067085 | 0.13485163 | 3 | 0.00442961 | -0.00325848 |
| original_firstorder_Entropy | 0.000585566 | 0.00025356 | 0.02859548 | 3 | 0.002038478 | -0.00086735 |
| wavelet-HL_glcm_SumSquares | 0.000585566 | 0.00025356 | 0.02859548 | 3 | 0.002038478 | -0.00086735 |
| gradient_gldm_HighGrayLevelEmphasis | 0.000585566 | 0.00050712 | 0.09175171 | 3 | 0.00349139 | -0.00232026 |
| wavelet-HH_gldm_LargeDependenceHighGrayLevelEmphasis | 0.000585566 | 0.00050712 | 0.09175171 | 3 | 0.00349139 | -0.00232026 |
| wavelet-HH_glrlm_GrayLevelNonUniformity | 0.000585566 | 0.00050712 | 0.09175171 | 3 | 0.00349139 | -0.00232026 |
| wavelet-HH_glrlm_ShortRunEmphasis | 0.000585566 | 0.00141175 | 0.27354459 | 3 | 0.008675039 | -0.00750391 |
| original_glszm_LowGrayLevelZoneEmphasis | 0.000585566 | 0.00025356 | 0.02859548 | 3 | 0.002038478 | -0.00086735 |
| wavelet-LL_glrlm_GrayLevelNonUniformityNormalized | 0.000585566 | 0.00025356 | 0.02859548 | 3 | 0.002038478 | -0.00086735 |
| square_firstorder_Median | 0.000439174 | 0 | 0.5 | 3 | 0.000439174 | 0.000439174 |
| exponential_glrlm_RunVariance | 0.000439174 | 0.00043917 | 0.11270167 | 3 | 0.002955692 | -0.00207734 |
| square_glrlm_GrayLevelVariance | 0.000439174 | 0 | 0.5 | 3 | 0.000439174 | 0.000439174 |
| square_glrlm_ShortRunEmphasis | 0.000439174 | 0.00076067 | 0.21132487 | 3 | 0.004797911 | -0.00391956 |
| wavelet-LH_glrlm_RunLengthNonUniformity | 0.000439174 | 0.00043917 | 0.11270167 | 3 | 0.002955692 | -0.00207734 |
| square_glrlm_LongRunHighGrayLevelEmphasis | 0.000439174 | 0.00076067 | 0.21132487 | 3 | 0.004797911 | -0.00391956 |
| wavelet-LH_glcm_ClusterProminence | 0.000439174 | 0.00043917 | 0.11270167 | 3 | 0.002955692 | -0.00207734 |
| gradient_firstorder_Maximum | 0.000439174 | 0.00043917 | 0.11270167 | 3 | 0.002955692 | -0.00207734 |
| square_firstorder_Energy | 0.000439174 | 0.00043917 | 0.11270167 | 3 | 0.002955692 | -0.00207734 |
| wavelet-LH_firstorder_Variance | 0.000439174 | 0.00043917 | 0.11270167 | 3 | 0.002955692 | -0.00207734 |
| original_glszm_GrayLevelNonUniformityNormalized | 0.000439174 | 0.00076067 | 0.21132487 | 3 | 0.004797911 | -0.00391956 |
| wavelet-HL_gldm_DependenceNonUniformity | 0.000439174 | 0.00076067 | 0.21132487 | 3 | 0.004797911 | -0.00391956 |
| original_glrlm_RunLengthNonUniformity | 0.000439174 | 0.00076067 | 0.21132487 | 3 | 0.004797911 | -0.00391956 |
| gradient_gldm_DependenceVariance | 0.000439174 | 0.00152135 | 0.33333333 | 3 | 0.009156648 | -0.0082783 |
| gradient_gldm_GrayLevelVariance | 0.000439174 | 0.00043917 | 0.11270167 | 3 | 0.002955692 | -0.00207734 |
| original_gldm_SmallDependenceHighGrayLevelEmphasis | 0.000439174 | 6.41E-17 | 3.55E-27 | 3 | 0.000439174 | 0.000439174 |
| logarithm_glcm_Imc1 | 0.000439174 | 0.00043917 | 0.11270167 | 3 | 0.002955692 | -0.00207734 |
| squareroot_glcm_Correlation | 0.000439174 | 0.00043917 | 0.11270167 | 3 | 0.002955692 | -0.00207734 |
| exponential_firstorder_Minimum | 0.000439174 | 0.00076067 | 0.21132487 | 3 | 0.004797911 | -0.00391956 |
| square_ngtdm_Coarseness | 0.000439174 | 0.00076067 | 0.21132487 | 3 | 0.004797911 | -0.00391956 |
| wavelet-HL_glrlm_ShortRunLowGrayLevelEmphasis | 0.000439174 | 0.00043917 | 0.11270167 | 3 | 0.002955692 | -0.00207734 |
| gradient_glcm_ClusterTendency | 0.000439174 | 0 | 0.5 | 3 | 0.000439174 | 0.000439174 |
| squareroot_gldm_DependenceNonUniformityNormalized | 0.000292783 | 0.00025356 | 0.09175171 | 3 | 0.001745695 | -0.00116013 |
| wavelet-HL_glcm_DifferenceAverage | 0.000292783 | 0.00025356 | 0.09175171 | 3 | 0.001745695 | -0.00116013 |
| square_glszm_ZonePercentage | 0.000292783 | 0.00025356 | 0.09175171 | 3 | 0.001745695 | -0.00116013 |
| wavelet-HH_firstorder_RootMeanSquared | 0.000292783 | 0.00025356 | 0.09175171 | 3 | 0.001745695 | -0.00116013 |
| wavelet-HH_glrlm_RunLengthNonUniformityNormalized | 0.000292783 | 0.00025356 | 0.09175171 | 3 | 0.001745695 | -0.00116013 |
| wavelet-HL_glrlm_HighGrayLevelRunEmphasis | 0.000292783 | 0.00067085 | 0.26429774 | 3 | 0.004136827 | -0.00355126 |
| squareroot_gldm_LowGrayLevelEmphasis | 0.000292783 | 0.00025356 | 0.09175171 | 3 | 0.001745695 | -0.00116013 |
| gradient_glrlm_RunLengthNonUniformityNormalized | 0.000292783 | 0.00025356 | 0.09175171 | 3 | 0.001745695 | -0.00116013 |
| gradient_gldm_LargeDependenceHighGrayLevelEmphasis | 0.000292783 | 0.00025356 | 0.09175171 | 3 | 0.001745695 | -0.00116013 |
| gradient_firstorder_Median | 0.000292783 | 0.00025356 | 0.09175171 | 3 | 0.001745695 | -0.00116013 |
| squareroot_glrlm_RunLengthNonUniformityNormalized | 0.000292783 | 0.00025356 | 0.09175171 | 3 | 0.001745695 | -0.00116013 |
| squareroot_glszm_LowGrayLevelZoneEmphasis | 0.000292783 | 0.00025356 | 0.09175171 | 3 | 0.001745695 | -0.00116013 |
| squareroot_glrlm_GrayLevelNonUniformityNormalized | 0.000292783 | 0.00025356 | 0.09175171 | 3 | 0.001745695 | -0.00116013 |
| square_gldm_GrayLevelNonUniformity | 0.000292783 | 0.00050712 | 0.21132487 | 3 | 0.003198607 | -0.00261304 |
| wavelet-LH_glcm_Imc1 | 0.000292783 | 0.00067085 | 0.26429774 | 3 | 0.004136827 | -0.00355126 |
| gradient_glcm_JointAverage | 0.000292783 | 0.00025356 | 0.09175171 | 3 | 0.001745695 | -0.00116013 |
| square_glszm_GrayLevelVariance | 0.000292783 | 0.00067085 | 0.26429774 | 3 | 0.004136827 | -0.00355126 |
| squareroot_gldm_SmallDependenceHighGrayLevelEmphasis | 0.000292783 | 0.00091421 | 0.31742581 | 3 | 0.005531332 | -0.00494577 |
| original_glrlm_HighGrayLevelRunEmphasis | 0.000292783 | 0.0013417 | 0.37090056 | 3 | 0.007980872 | -0.00739531 |
| squareroot_firstorder_10Percentile | 0.000292783 | 0.00050712 | 0.21132487 | 3 | 0.003198607 | -0.00261304 |
| wavelet-HL_glcm_DifferenceEntropy | 0.000292783 | 0.00067085 | 0.26429774 | 3 | 0.004136827 | -0.00355126 |
| exponential_glrlm_LongRunHighGrayLevelEmphasis | 0.000292783 | 0.00025356 | 0.09175171 | 3 | 0.001745695 | -0.00116013 |
| logarithm_glszm_GrayLevelNonUniformity | 0.000292783 | 0.00050712 | 0.21132487 | 3 | 0.003198607 | -0.00261304 |
| square_ngtdm_Strength | 0.000292783 | 0.00067085 | 0.26429774 | 3 | 0.004136827 | -0.00355126 |
| squareroot_glcm_SumEntropy | 0.000292783 | 0.00091421 | 0.31742581 | 3 | 0.005531332 | -0.00494577 |
| wavelet-LL_gldm_LargeDependenceEmphasis | 0.000292783 | 0.00067085 | 0.26429774 | 3 | 0.004136827 | -0.00355126 |
| wavelet-HH_glcm_Idmn | 0.000292783 | 0.00067085 | 0.26429774 | 3 | 0.004136827 | -0.00355126 |
| wavelet-LL_glcm_DifferenceVariance | 0.000292783 | 0.00067085 | 0.26429774 | 3 | 0.004136827 | -0.00355126 |
| wavelet-HH_glcm_Imc2 | 0.000292783 | 0.00050712 | 0.21132487 | 3 | 0.003198607 | -0.00261304 |
| logarithm_ngtdm_Strength | 0.000292783 | 0.00025356 | 0.09175171 | 3 | 0.001745695 | -0.00116013 |
| gradient_gldm_SmallDependenceLowGrayLevelEmphasis | 0.000292783 | 0.00025356 | 0.09175171 | 3 | 0.001745695 | -0.00116013 |
| squareroot_firstorder_MeanAbsoluteDeviation | 0.000292783 | 0.00025356 | 0.09175171 | 3 | 0.001745695 | -0.00116013 |
| original_ngtdm_Strength | 0.000292783 | 0.00025356 | 0.09175171 | 3 | 0.001745695 | -0.00116013 |
| wavelet-HL_glszm_LargeAreaHighGrayLevelEmphasis | 0.000292783 | 0.00025356 | 0.09175171 | 3 | 0.001745695 | -0.00116013 |
| wavelet-LH_glcm_DifferenceVariance | 0.000292783 | 0.00025356 | 0.09175171 | 3 | 0.001745695 | -0.00116013 |
| location | 0.000292783 | 0.00141175 | 0.37690851 | 3 | 0.008382256 | -0.00779669 |
| wavelet-LL_ngtdm_Strength | 0.000292783 | 0.00025356 | 0.09175171 | 3 | 0.001745695 | -0.00116013 |
| wavelet-LL_glszm_LargeAreaHighGrayLevelEmphasis | 0.000292783 | 0.0017749 | 0.40098525 | 3 | 0.010463168 | -0.0098776 |
| gradient_firstorder_Skewness | 0.000292783 | 0.00101423 | 0.33333333 | 3 | 0.006104432 | -0.00551887 |
| wavelet-LL_glrlm_RunEntropy | 0.000292783 | 0.00025356 | 0.09175171 | 3 | 0.001745695 | -0.00116013 |
| logarithm_glcm_Correlation | 0.000292783 | 0.00241878 | 0.42667644 | 3 | 0.014152682 | -0.01356712 |
| squareroot_ngtdm_Strength | 0.000146391 | 0.00025356 | 0.21132487 | 3 | 0.001599304 | -0.00130652 |
| wavelet-LL_glrlm_ShortRunHighGrayLevelEmphasis | 0.000146391 | 0.00025356 | 0.21132487 | 3 | 0.001599304 | -0.00130652 |
| gradient_glrlm_ShortRunEmphasis | 0.000146391 | 0.00025356 | 0.21132487 | 3 | 0.001599304 | -0.00130652 |
| squareroot_glcm_ClusterProminence | 0.000146391 | 0.00025356 | 0.21132487 | 3 | 0.001599304 | -0.00130652 |
| exponential_firstorder_RootMeanSquared | 0.000146391 | 0.00067085 | 0.37090056 | 3 | 0.003990436 | -0.00369765 |
| wavelet-HH_glszm_SmallAreaHighGrayLevelEmphasis | 0.000146391 | 0.00025356 | 0.21132487 | 3 | 0.001599304 | -0.00130652 |
| wavelet-LL_gldm_SmallDependenceHighGrayLevelEmphasis | 0.000146391 | 0.00025356 | 0.21132487 | 3 | 0.001599304 | -0.00130652 |
| original_glrlm_ShortRunLowGrayLevelEmphasis | 0.000146391 | 0.00067085 | 0.37090056 | 3 | 0.003990436 | -0.00369765 |
| logarithm_glrlm_LowGrayLevelRunEmphasis | 0.000146391 | 0.00067085 | 0.37090056 | 3 | 0.003990436 | -0.00369765 |
| original_glszm_GrayLevelNonUniformity | 0.000146391 | 0.00050712 | 0.33333333 | 3 | 0.003052216 | -0.00275943 |
| wavelet-HH_gldm_DependenceEntropy | 0.000146391 | 0.00025356 | 0.21132487 | 3 | 0.001599304 | -0.00130652 |
| wavelet-LL_gldm_GrayLevelVariance | 0.000146391 | 0.00091421 | 0.40377496 | 3 | 0.005384941 | -0.00509216 |
| original_shape2D_Perimeter | 0.000146391 | 0.00050712 | 0.33333333 | 3 | 0.003052216 | -0.00275943 |
| logarithm_glszm_SizeZoneNonUniformity | 0.000146391 | 0.00025356 | 0.21132487 | 3 | 0.001599304 | -0.00130652 |
| logarithm_gldm_SmallDependenceEmphasis | 0.000146391 | 0.00025356 | 0.21132487 | 3 | 0.001599304 | -0.00130652 |
| wavelet-HH_glcm_JointAverage | 0.000146391 | 0.00025356 | 0.21132487 | 3 | 0.001599304 | -0.00130652 |
| logarithm_firstorder_MeanAbsoluteDeviation | 0.000146391 | 0.00025356 | 0.21132487 | 3 | 0.001599304 | -0.00130652 |
| logarithm_glszm_SmallAreaEmphasis | 0.000146391 | 0.00025356 | 0.21132487 | 3 | 0.001599304 | -0.00130652 |
| original_firstorder_MeanAbsoluteDeviation | 0.000146391 | 0.00025356 | 0.21132487 | 3 | 0.001599304 | -0.00130652 |
| wavelet-LL_glszm_GrayLevelVariance | 0.000146391 | 0.00110523 | 0.41993592 | 3 | 0.006479489 | -0.00618671 |
| original_shape2D_MajorAxisLength | 0.000146391 | 0.00067085 | 0.37090056 | 3 | 0.003990436 | -0.00369765 |
| wavelet-LL_glszm_SizeZoneNonUniformity | 0.000146391 | 0.00067085 | 0.37090056 | 3 | 0.003990436 | -0.00369765 |
| squareroot_glszm_LargeAreaHighGrayLevelEmphasis | 0.000146391 | 0.00101423 | 0.41296117 | 3 | 0.00595804 | -0.00566526 |
| wavelet-LL_glcm_Idm | 0.000146391 | 0.00025356 | 0.21132487 | 3 | 0.001599304 | -0.00130652 |
| squareroot_glrlm_ShortRunEmphasis | 0.000146391 | 0.00067085 | 0.37090056 | 3 | 0.003990436 | -0.00369765 |
| wavelet-HL_glcm_JointAverage | 0.000146391 | 0.00067085 | 0.37090056 | 3 | 0.003990436 | -0.00369765 |
| wavelet-LH_ngtdm_Contrast | 0.000146391 | 0.00025356 | 0.21132487 | 3 | 0.001599304 | -0.00130652 |
| logarithm_glcm_JointEntropy | 0.000146391 | 0.00050712 | 0.33333333 | 3 | 0.003052216 | -0.00275943 |
| square_glcm_MCC | 0.000146391 | 0.00067085 | 0.37090056 | 3 | 0.003990436 | -0.00369765 |
| wavelet-LH_glcm_Idm | 0.000146391 | 0.00025356 | 0.21132487 | 3 | 0.001599304 | -0.00130652 |
| gradient_firstorder_10Percentile | 0.000146391 | 0.00067085 | 0.37090056 | 3 | 0.003990436 | -0.00369765 |
| original_glcm_Id | 0.000146391 | 0.00025356 | 0.21132487 | 3 | 0.001599304 | -0.00130652 |
| wavelet-HH_glcm_Id | 0.000146391 | 0.00067085 | 0.37090056 | 3 | 0.003990436 | -0.00369765 |
| gradient_glcm_Autocorrelation | 0.000146391 | 0.00067085 | 0.37090056 | 3 | 0.003990436 | -0.00369765 |
| wavelet-HL_gldm_SmallDependenceHighGrayLevelEmphasis | 0.000146391 | 0.00101423 | 0.41296117 | 3 | 0.00595804 | -0.00566526 |
| wavelet-HL_gldm_HighGrayLevelEmphasis | 0.000146391 | 0.00025356 | 0.21132487 | 3 | 0.001599304 | -0.00130652 |
| logarithm_glszm_LargeAreaHighGrayLevelEmphasis | 0.000146391 | 0.00067085 | 0.37090056 | 3 | 0.003990436 | -0.00369765 |
| wavelet-LH_firstorder_Range | 0.000146391 | 0.00067085 | 0.37090056 | 3 | 0.003990436 | -0.00369765 |
| square_glrlm_RunLengthNonUniformity | 0.000146391 | 0.00050712 | 0.33333333 | 3 | 0.003052216 | -0.00275943 |
| squareroot_firstorder_Median | 0 | 0 | 0.5 | 3 | 0 | 0 |
| squareroot_firstorder_Mean | 0 | 0 | 0.5 | 3 | 0 | 0 |
| squareroot_glcm_JointAverage | 0 | 0 | 0.5 | 3 | 0 | 0 |
| square_glcm_InverseVariance | 0 | 0 | 0.5 | 3 | 0 | 0 |
| square_glcm_Idmn | 0 | 0 | 0.5 | 3 | 0 | 0 |
| square_glcm_Idm | 0 | 0 | 0.5 | 3 | 0 | 0 |
| squareroot_glcm_MaximumProbability | 0 | 0.00116195 | 0.5 | 3 | 0.00665808 | -0.00665808 |
| square_glcm_Id | 0 | 0 | 0.5 | 3 | 0 | 0 |
| square_glcm_DifferenceVariance | 0 | 0 | 0.5 | 3 | 0 | 0 |
| square_glcm_ClusterTendency | 0 | 0 | 0.5 | 3 | 0 | 0 |
| square_firstorder_Variance | 0 | 0 | 0.5 | 3 | 0 | 0 |
| square_firstorder_Uniformity | 0 | 0 | 0.5 | 3 | 0 | 0 |
| square_firstorder_RootMeanSquared | 0 | 0 | 0.5 | 3 | 0 | 0 |
| square_firstorder_RobustMeanAbsoluteDeviation | 0 | 0 | 0.5 | 3 | 0 | 0 |
| square_firstorder_Range | 0 | 0 | 0.5 | 3 | 0 | 0 |
| square_firstorder_Minimum | 0 | 0 | 0.5 | 3 | 0 | 0 |
| square_firstorder_Mean | 0 | 0 | 0.5 | 3 | 0 | 0 |
| square_firstorder_Maximum | 0 | 0 | 0.5 | 3 | 0 | 0 |
| squareroot_gldm_DependenceNonUniformity | 0 | 0 | 0.5 | 3 | 0 | 0 |
| square_firstorder_90Percentile | 0 | 0 | 0.5 | 3 | 0 | 0 |
| squareroot_gldm_GrayLevelNonUniformity | 0 | 0 | 0.5 | 3 | 0 | 0 |
| original_shape2D_PixelSurface | 0 | 0 | 0.5 | 3 | 0 | 0 |
| squareroot_gldm_HighGrayLevelEmphasis | 0 | 0 | 0.5 | 3 | 0 | 0 |
| original_shape2D_PerimeterSurfaceRatio | 0 | 0 | 0.5 | 3 | 0 | 0 |
| original_shape2D_MeshSurface | 0 | 0 | 0.5 | 3 | 0 | 0 |
| squareroot_glcm_Imc2 | 0 | 0 | 0.5 | 3 | 0 | 0 |
| square_glcm_JointAverage | 0 | 0 | 0.5 | 3 | 0 | 0 |
| square_glcm_JointEnergy | 0 | 0 | 0.5 | 3 | 0 | 0 |
| square_glrlm_GrayLevelNonUniformity | 0 | 0 | 0.5 | 3 | 0 | 0 |
| square_glszm_ZoneVariance | 0 | 0 | 0.5 | 3 | 0 | 0 |
| square_glszm_LargeAreaLowGrayLevelEmphasis | 0 | 0 | 0.5 | 3 | 0 | 0 |
| square_glszm_LargeAreaHighGrayLevelEmphasis | 0 | 0 | 0.5 | 3 | 0 | 0 |
| squareroot_glcm_ClusterTendency | 0 | 0 | 0.5 | 3 | 0 | 0 |
| square_glszm_LargeAreaEmphasis | 0 | 0 | 0.5 | 3 | 0 | 0 |
| square_glrlm_ShortRunLowGrayLevelEmphasis | 0 | 0 | 0.5 | 3 | 0 | 0 |
| square_glrlm_ShortRunHighGrayLevelEmphasis | 0 | 0.00043917 | 0.5 | 3 | 0.002516518 | -0.00251652 |
| square_glrlm_LowGrayLevelRunEmphasis | 0 | 0 | 0.5 | 3 | 0 | 0 |
| square_glrlm_LongRunLowGrayLevelEmphasis | 0 | 0 | 0.5 | 3 | 0 | 0 |
| square_glrlm_LongRunEmphasis | 0 | 0 | 0.5 | 3 | 0 | 0 |
| square_gldm_LowGrayLevelEmphasis | 0 | 0 | 0.5 | 3 | 0 | 0 |
| square_glcm_MaximumProbability | 0 | 0 | 0.5 | 3 | 0 | 0 |
| square_gldm_LargeDependenceLowGrayLevelEmphasis | 0 | 0 | 0.5 | 3 | 0 | 0 |
| square_gldm_HighGrayLevelEmphasis | 0 | 0 | 0.5 | 3 | 0 | 0 |
| square_gldm_GrayLevelVariance | 0 | 0 | 0.5 | 3 | 0 | 0 |
| squareroot_glcm_DifferenceEntropy | 0 | 0.00043917 | 0.5 | 3 | 0.002516518 | -0.00251652 |
| squareroot_glcm_Idm | 0 | 0 | 0.5 | 3 | 0 | 0 |
| squareroot_glcm_Imc1 | 0 | 0.00043917 | 0.5 | 3 | 0.002516518 | -0.00251652 |
| square_gldm_DependenceNonUniformity | 0 | 0 | 0.5 | 3 | 0 | 0 |
| square_gldm_DependenceEntropy | 0 | 0 | 0.5 | 3 | 0 | 0 |
| square_glcm_SumSquares | 0 | 0 | 0.5 | 3 | 0 | 0 |
| square_glcm_SumEntropy | 0 | 0 | 0.5 | 3 | 0 | 0 |
| squareroot_gldm_LargeDependenceLowGrayLevelEmphasis | 0 | 0 | 0.5 | 3 | 0 | 0 |
| squareroot_gldm_SmallDependenceEmphasis | 0 | 0 | 0.5 | 3 | 0 | 0 |
| wavelet-LH_glszm_ZonePercentage | 0 | 0 | 0.5 | 3 | 0 | 0 |
| wavelet-LH_glszm_LargeAreaHighGrayLevelEmphasis | 0 | 0 | 0.5 | 3 | 0 | 0 |
| wavelet-LH_glszm_GrayLevelVariance | 0 | 0 | 0.5 | 3 | 0 | 0 |
| wavelet-LH_glszm_GrayLevelNonUniformityNormalized | 0 | 0 | 0.5 | 3 | 0 | 0 |
| wavelet-LH_glrlm_RunVariance | 0 | 0 | 0.5 | 3 | 0 | 0 |
| wavelet-LH_glrlm_RunLengthNonUniformityNormalized | 0 | 0 | 0.5 | 3 | 0 | 0 |
| wavelet-LH_glrlm_HighGrayLevelRunEmphasis | 0 | 0 | 0.5 | 3 | 0 | 0 |
| wavelet-LH_glrlm_GrayLevelNonUniformity | 0 | 0 | 0.5 | 3 | 0 | 0 |
| wavelet-LH_gldm_SmallDependenceEmphasis | 0 | 0 | 0.5 | 3 | 0 | 0 |
| wavelet-LH_gldm_HighGrayLevelEmphasis | 0 | 0 | 0.5 | 3 | 0 | 0 |
| wavelet-LH_gldm_GrayLevelVariance | 0 | 0 | 0.5 | 3 | 0 | 0 |
| wavelet-LH_glcm_SumSquares | 0 | 0 | 0.5 | 3 | 0 | 0 |
| wavelet-LH_glcm_JointEnergy | 0 | 0 | 0.5 | 3 | 0 | 0 |
| wavelet-LH_glcm_Idn | 0 | 0 | 0.5 | 3 | 0 | 0 |
| wavelet-LH_glcm_DifferenceEntropy | 0 | 0 | 0.5 | 3 | 0 | 0 |
| wavelet-LH_glcm_DifferenceAverage | 0 | 0 | 0.5 | 3 | 0 | 0 |
| wavelet-LH_glcm_Contrast | 0 | 0 | 0.5 | 3 | 0 | 0 |
| wavelet-LH_glcm_ClusterTendency | 0 | 0 | 0.5 | 3 | 0 | 0 |
| wavelet-LH_firstorder_RobustMeanAbsoluteDeviation | 0 | 0 | 0.5 | 3 | 0 | 0 |
| wavelet-LH_firstorder_Median | 0 | 0 | 0.5 | 3 | 0 | 0 |
| wavelet-LH_firstorder_InterquartileRange | 0 | 0 | 0.5 | 3 | 0 | 0 |
| wavelet-LH_firstorder_Entropy | 0 | 0 | 0.5 | 3 | 0 | 0 |
| wavelet-LH_firstorder_90Percentile | 0 | 0.00043917 | 0.5 | 3 | 0.002516518 | -0.00251652 |
| wavelet-HL_ngtdm_Complexity | 0 | 0 | 0.5 | 3 | 0 | 0 |
| wavelet-HL_ngtdm_Coarseness | 0 | 0 | 0.5 | 3 | 0 | 0 |
| wavelet-HL_ngtdm_Busyness | 0 | 0 | 0.5 | 3 | 0 | 0 |
| wavelet-HL_glszm_ZoneVariance | 0 | 0 | 0.5 | 3 | 0 | 0 |
| wavelet-HL_glszm_ZonePercentage | 0 | 0 | 0.5 | 3 | 0 | 0 |
| wavelet-LH_glszm_ZoneEntropy | 0 | 0 | 0.5 | 3 | 0 | 0 |
| wavelet-LH_glszm_ZoneVariance | 0 | 0 | 0.5 | 3 | 0 | 0 |
| wavelet-HL_glszm_LowGrayLevelZoneEmphasis | 0 | 0 | 0.5 | 3 | 0 | 0 |
| wavelet-LL_firstorder_MeanAbsoluteDeviation | 0 | 0 | 0.5 | 3 | 0 | 0 |
| wavelet-LL_ngtdm_Coarseness | 0 | 0 | 0.5 | 3 | 0 | 0 |
| wavelet-LL_glszm_ZonePercentage | 0 | 0 | 0.5 | 3 | 0 | 0 |
| wavelet-LL_glszm_SmallAreaHighGrayLevelEmphasis | 0 | 0 | 0.5 | 3 | 0 | 0 |
| wavelet-LL_glszm_SmallAreaEmphasis | 0 | 0 | 0.5 | 3 | 0 | 0 |
| wavelet-LL_glszm_LargeAreaEmphasis | 0 | 0 | 0.5 | 3 | 0 | 0 |
| wavelet-LL_glszm_HighGrayLevelZoneEmphasis | 0 | 0 | 0.5 | 3 | 0 | 0 |
| wavelet-LL_glrlm_ShortRunEmphasis | 0 | 0 | 0.5 | 3 | 0 | 0 |
| wavelet-LL_glrlm_RunVariance | 0 | 0 | 0.5 | 3 | 0 | 0 |
| wavelet-LL_glrlm_HighGrayLevelRunEmphasis | 0 | 0 | 0.5 | 3 | 0 | 0 |
| wavelet-LL_glrlm_GrayLevelNonUniformity | 0 | 0 | 0.5 | 3 | 0 | 0 |
| wavelet-LL_gldm_SmallDependenceEmphasis | 0 | 0 | 0.5 | 3 | 0 | 0 |
| wavelet-LL_gldm_LowGrayLevelEmphasis | 0 | 0 | 0.5 | 3 | 0 | 0 |
| wavelet-LL_gldm_LargeDependenceLowGrayLevelEmphasis | 0 | 0 | 0.5 | 3 | 0 | 0 |
| wavelet-LL_gldm_DependenceNonUniformityNormalized | 0 | 0 | 0.5 | 3 | 0 | 0 |
| wavelet-LL_glcm_SumSquares | 0 | 0 | 0.5 | 3 | 0 | 0 |
| wavelet-LL_glcm_JointEnergy | 0 | 0 | 0.5 | 3 | 0 | 0 |
| wavelet-LL_glcm_Idn | 0 | 0 | 0.5 | 3 | 0 | 0 |
| wavelet-LL_glcm_DifferenceEntropy | 0 | 0 | 0.5 | 3 | 0 | 0 |
| wavelet-LL_glcm_DifferenceAverage | 0 | 0 | 0.5 | 3 | 0 | 0 |
| wavelet-LL_glcm_Contrast | 0 | 0 | 0.5 | 3 | 0 | 0 |
| wavelet-LL_glcm_ClusterTendency | 0 | 0 | 0.5 | 3 | 0 | 0 |
| wavelet-LL_glcm_Autocorrelation | 0 | 0 | 0.5 | 3 | 0 | 0 |
| wavelet-LL_firstorder_Variance | 0 | 0 | 0.5 | 3 | 0 | 0 |
| wavelet-LL_firstorder_Skewness | 0 | 0 | 0.5 | 3 | 0 | 0 |
| wavelet-LL_firstorder_Minimum | 0 | 0 | 0.5 | 3 | 0 | 0 |
| wavelet-LL_firstorder_Median | 0 | 0 | 0.5 | 3 | 0 | 0 |
| wavelet-HL_glszm_SmallAreaEmphasis | 0 | 0 | 0.5 | 3 | 0 | 0 |
| wavelet-HL_glszm_LargeAreaEmphasis | 0 | 0 | 0.5 | 3 | 0 | 0 |
| squareroot_gldm_SmallDependenceLowGrayLevelEmphasis | 0 | 0 | 0.5 | 3 | 0 | 0 |
| wavelet-HH_glrlm_RunVariance | 0 | 0 | 0.5 | 3 | 0 | 0 |
| wavelet-HH_glrlm_LongRunLowGrayLevelEmphasis | 0 | 0 | 0.5 | 3 | 0 | 0 |
| wavelet-HH_glrlm_GrayLevelVariance | 0 | 0 | 0.5 | 3 | 0 | 0 |
| wavelet-HH_gldm_SmallDependenceEmphasis | 0 | 0.00043917 | 0.5 | 3 | 0.002516518 | -0.00251652 |
| wavelet-HH_gldm_GrayLevelVariance | 0 | 0 | 0.5 | 3 | 0 | 0 |
| wavelet-HH_gldm_GrayLevelNonUniformity | 0 | 0 | 0.5 | 3 | 0 | 0 |
| wavelet-HH_gldm_DependenceNonUniformity | 0 | 0 | 0.5 | 3 | 0 | 0 |
| wavelet-HH_glcm_SumSquares | 0 | 0 | 0.5 | 3 | 0 | 0 |
| wavelet-HH_glcm_MCC | 0 | 0.00043917 | 0.5 | 3 | 0.002516518 | -0.00251652 |
| wavelet-HH_glcm_JointEntropy | 0 | 0 | 0.5 | 3 | 0 | 0 |
| wavelet-HH_glcm_JointEnergy | 0 | 0 | 0.5 | 3 | 0 | 0 |
| wavelet-HH_glcm_DifferenceEntropy | 0 | 0 | 0.5 | 3 | 0 | 0 |
| wavelet-HH_glcm_DifferenceAverage | 0 | 0 | 0.5 | 3 | 0 | 0 |
| wavelet-HH_glcm_Correlation | 0 | 0 | 0.5 | 3 | 0 | 0 |
| wavelet-HH_glcm_ClusterTendency | 0 | 0 | 0.5 | 3 | 0 | 0 |
| wavelet-HH_glcm_Autocorrelation | 0 | 0 | 0.5 | 3 | 0 | 0 |
| wavelet-HH_firstorder_Variance | 0 | 0 | 0.5 | 3 | 0 | 0 |
| wavelet-HH_firstorder_Uniformity | 0 | 0 | 0.5 | 3 | 0 | 0 |
| squareroot_ngtdm_Contrast | 0 | 0 | 0.5 | 3 | 0 | 0 |
| squareroot_ngtdm_Coarseness | 0 | 0 | 0.5 | 3 | 0 | 0 |
| squareroot_glszm_ZonePercentage | 0 | 0 | 0.5 | 3 | 0 | 0 |
| squareroot_glszm_SizeZoneNonUniformity | 0 | 0 | 0.5 | 3 | 0 | 0 |
| squareroot_glszm_GrayLevelNonUniformity | 0 | 0 | 0.5 | 3 | 0 | 0 |
| squareroot_glrlm_RunLengthNonUniformity | 0 | 0 | 0.5 | 3 | 0 | 0 |
| squareroot_glrlm_LowGrayLevelRunEmphasis | 0 | 0 | 0.5 | 3 | 0 | 0 |
| squareroot_glrlm_LongRunLowGrayLevelEmphasis | 0 | 0 | 0.5 | 3 | 0 | 0 |
| squareroot_glrlm_LongRunHighGrayLevelEmphasis | 0 | 0 | 0.5 | 3 | 0 | 0 |
| squareroot_glrlm_LongRunEmphasis | 0 | 0 | 0.5 | 3 | 0 | 0 |
| wavelet-HH_glrlm_RunLengthNonUniformity | 0 | 0 | 0.5 | 3 | 0 | 0 |
| original_ngtdm_Complexity | 0 | 0 | 0.5 | 3 | 0 | 0 |
| wavelet-HL_glszm_HighGrayLevelZoneEmphasis | 0 | 0.00043917 | 0.5 | 3 | 0.002516518 | -0.00251652 |
| wavelet-HH_glszm_LargeAreaEmphasis | 0 | 0 | 0.5 | 3 | 0 | 0 |
| wavelet-HL_glszm_GrayLevelNonUniformity | 0 | 0 | 0.5 | 3 | 0 | 0 |
| wavelet-HL_glrlm_ShortRunHighGrayLevelEmphasis | 0 | 0 | 0.5 | 3 | 0 | 0 |
| wavelet-HL_glrlm_ShortRunEmphasis | 0 | 0 | 0.5 | 3 | 0 | 0 |
| wavelet-HL_glrlm_RunPercentage | 0 | 0 | 0.5 | 3 | 0 | 0 |
| wavelet-HL_glrlm_RunLengthNonUniformity | 0 | 0 | 0.5 | 3 | 0 | 0 |
| wavelet-HL_glrlm_LongRunLowGrayLevelEmphasis | 0 | 0 | 0.5 | 3 | 0 | 0 |
| wavelet-HL_glrlm_GrayLevelVariance | 0 | 0 | 0.5 | 3 | 0 | 0 |
| wavelet-HL_glrlm_GrayLevelNonUniformityNormalized | 0 | 0 | 0.5 | 3 | 0 | 0 |
| wavelet-HL_gldm_LowGrayLevelEmphasis | 0 | 0 | 0.5 | 3 | 0 | 0 |
| wavelet-HL_gldm_GrayLevelVariance | 0 | 0 | 0.5 | 3 | 0 | 0 |
| wavelet-HL_gldm_GrayLevelNonUniformity | 0 | 0 | 0.5 | 3 | 0 | 0 |
| wavelet-HL_glcm_SumEntropy | 0 | 0 | 0.5 | 3 | 0 | 0 |
| wavelet-HL_glcm_JointEnergy | 0 | 0 | 0.5 | 3 | 0 | 0 |
| wavelet-HL_glcm_Idm | 0 | 0 | 0.5 | 3 | 0 | 0 |
| wavelet-HL_glcm_Id | 0 | 0 | 0.5 | 3 | 0 | 0 |
| wavelet-HL_glcm_DifferenceVariance | 0 | 0 | 0.5 | 3 | 0 | 0 |
| wavelet-HL_glcm_ClusterTendency | 0 | 0 | 0.5 | 3 | 0 | 0 |
| wavelet-HL_glcm_Autocorrelation | 0 | 0 | 0.5 | 3 | 0 | 0 |
| wavelet-HL_firstorder_Uniformity | 0 | 0 | 0.5 | 3 | 0 | 0 |
| wavelet-HL_firstorder_RootMeanSquared | 0 | 0.00043917 | 0.5 | 3 | 0.002516518 | -0.00251652 |
| wavelet-HL_firstorder_RobustMeanAbsoluteDeviation | 0 | 0 | 0.5 | 3 | 0 | 0 |
| wavelet-HL_firstorder_Mean | 0 | 0 | 0.5 | 3 | 0 | 0 |
| wavelet-HL_firstorder_Entropy | 0 | 0 | 0.5 | 3 | 0 | 0 |
| wavelet-HH_ngtdm_Coarseness | 0 | 0 | 0.5 | 3 | 0 | 0 |
| wavelet-HH_glszm_ZoneVariance | 0 | 0 | 0.5 | 3 | 0 | 0 |
| wavelet-HH_glszm_SmallAreaEmphasis | 0 | 0 | 0.5 | 3 | 0 | 0 |
| wavelet-HH_glszm_LargeAreaLowGrayLevelEmphasis | 0 | 0 | 0.5 | 3 | 0 | 0 |
| wavelet-HH_glrlm_ShortRunLowGrayLevelEmphasis | 0 | 0 | 0.5 | 3 | 0 | 0 |
| gradient_glrlm_RunVariance | 0 | 0 | 0.5 | 3 | 0 | 0 |
| exponential_glrlm_GrayLevelNonUniformity | 0 | 0 | 0.5 | 3 | 0 | 0 |
| logarithm_glszm_SizeZoneNonUniformityNormalized | 0 | 0 | 0.5 | 3 | 0 | 0 |
| exponential_gldm_SmallDependenceEmphasis | 0 | 0 | 0.5 | 3 | 0 | 0 |
| gradient_glcm_InverseVariance | 0 | 0 | 0.5 | 3 | 0 | 0 |
| gradient_glcm_Imc2 | 0 | 0 | 0.5 | 3 | 0 | 0 |
| logarithm_glcm_Autocorrelation | 0 | 0 | 0.5 | 3 | 0 | 0 |
| gradient_glcm_Idn | 0 | 0 | 0.5 | 3 | 0 | 0 |
| exponential_gldm_SmallDependenceHighGrayLevelEmphasis | 0 | 0 | 0.5 | 3 | 0 | 0 |
| gradient_glcm_Id | 0 | 0 | 0.5 | 3 | 0 | 0 |
| logarithm_glszm_GrayLevelNonUniformityNormalized | 0 | 0 | 0.5 | 3 | 0 | 0 |
| logarithm_glszm_SmallAreaHighGrayLevelEmphasis | 0 | 0 | 0.5 | 3 | 0 | 0 |
| logarithm_glrlm_ShortRunLowGrayLevelEmphasis | 0 | 0.00043917 | 0.5 | 3 | 0.002516518 | -0.00251652 |
| logarithm_glrlm_ShortRunHighGrayLevelEmphasis | 0 | 0 | 0.5 | 3 | 0 | 0 |
| exponential_glrlm_GrayLevelNonUniformityNormalized | 0 | 0 | 0.5 | 3 | 0 | 0 |
| logarithm_glcm_ClusterTendency | 0 | 0 | 0.5 | 3 | 0 | 0 |
| gradient_glcm_DifferenceEntropy | 0 | 0 | 0.5 | 3 | 0 | 0 |
| exponential_glrlm_GrayLevelVariance | 0 | 0 | 0.5 | 3 | 0 | 0 |
| logarithm_glrlm_RunVariance | 0 | 0 | 0.5 | 3 | 0 | 0 |
| exponential_glrlm_HighGrayLevelRunEmphasis | 0 | 0 | 0.5 | 3 | 0 | 0 |
| exponential_gldm_LowGrayLevelEmphasis | 0 | 0 | 0.5 | 3 | 0 | 0 |
| exponential_gldm_LargeDependenceLowGrayLevelEmphasis | 0 | 0 | 0.5 | 3 | 0 | 0 |
| exponential_glcm_JointAverage | 0 | 0 | 0.5 | 3 | 0 | 0 |
| gradient_glcm_MaximumProbability | 0 | 0 | 0.5 | 3 | 0 | 0 |
| exponential_glcm_JointEnergy | 0 | 0 | 0.5 | 3 | 0 | 0 |
| exponential_glcm_JointEntropy | 0 | 0 | 0.5 | 3 | 0 | 0 |
| exponential_ngtdm_Coarseness | 0 | 0 | 0.5 | 3 | 0 | 0 |
| exponential_glcm_MaximumProbability | 0 | 0 | 0.5 | 3 | 0 | 0 |
| original_firstorder_Energy | 0 | 0 | 0.5 | 3 | 0 | 0 |
| exponential_glcm_SumEntropy | 0 | 0 | 0.5 | 3 | 0 | 0 |
| gradient_gldm_DependenceEntropy | 0 | 0 | 0.5 | 3 | 0 | 0 |
| exponential_glcm_SumSquares | 0 | 0 | 0.5 | 3 | 0 | 0 |
| logarithm_firstorder_RobustMeanAbsoluteDeviation | 0 | 0 | 0.5 | 3 | 0 | 0 |
| gradient_glcm_JointEnergy | 0 | 0 | 0.5 | 3 | 0 | 0 |
| exponential_gldm_DependenceEntropy | 0 | 0 | 0.5 | 3 | 0 | 0 |
| exponential_gldm_DependenceNonUniformity | 0 | 0 | 0.5 | 3 | 0 | 0 |
| exponential_gldm_GrayLevelNonUniformity | 0 | 0 | 0.5 | 3 | 0 | 0 |
| gradient_glcm_JointEntropy | 0 | 0 | 0.5 | 3 | 0 | 0 |
| exponential_gldm_GrayLevelVariance | 0 | 0 | 0.5 | 3 | 0 | 0 |
| exponential_gldm_HighGrayLevelEmphasis | 0 | 0 | 0.5 | 3 | 0 | 0 |
| exponential_gldm_LargeDependenceEmphasis | 0 | 0 | 0.5 | 3 | 0 | 0 |
| exponential_gldm_LargeDependenceHighGrayLevelEmphasis | 0 | 0 | 0.5 | 3 | 0 | 0 |
| exponential_glrlm_LongRunEmphasis | 0 | 0 | 0.5 | 3 | 0 | 0 |
| logarithm_glrlm_RunLengthNonUniformityNormalized | 0 | 0 | 0.5 | 3 | 0 | 0 |
| gradient_glcm_DifferenceAverage | 0 | 0 | 0.5 | 3 | 0 | 0 |
| gradient_firstorder_Entropy | 0 | 0.00043917 | 0.5 | 3 | 0.002516518 | -0.00251652 |
| logarithm_gldm_GrayLevelVariance | 0 | 0 | 0.5 | 3 | 0 | 0 |
| logarithm_gldm_GrayLevelNonUniformity | 0 | 0 | 0.5 | 3 | 0 | 0 |
| exponential_glszm_LargeAreaHighGrayLevelEmphasis | 0 | 0.00043917 | 0.5 | 3 | 0.002516518 | -0.00251652 |
| exponential_glszm_LargeAreaLowGrayLevelEmphasis | 0 | 0 | 0.5 | 3 | 0 | 0 |
| exponential_glszm_LowGrayLevelZoneEmphasis | 0 | 0 | 0.5 | 3 | 0 | 0 |
| logarithm_glcm_Idn | 0 | 0.00076067 | 0.5 | 3 | 0.004358737 | -0.00435874 |
| exponential_glszm_SizeZoneNonUniformity | 0 | 0 | 0.5 | 3 | 0 | 0 |
| exponential_glszm_SizeZoneNonUniformityNormalized | 0 | 0 | 0.5 | 3 | 0 | 0 |
| exponential_glszm_SmallAreaHighGrayLevelEmphasis | 0 | 0 | 0.5 | 3 | 0 | 0 |
| logarithm_glcm_DifferenceAverage | 0 | 0 | 0.5 | 3 | 0 | 0 |
| exponential_glszm_SmallAreaLowGrayLevelEmphasis | 0 | 0 | 0.5 | 3 | 0 | 0 |
| exponential_glszm_ZoneEntropy | 0 | 0 | 0.5 | 3 | 0 | 0 |
| exponential_ngtdm_Strength | 0 | 0 | 0.5 | 3 | 0 | 0 |
| exponential_glszm_ZonePercentage | 0 | 0 | 0.5 | 3 | 0 | 0 |
| exponential_glszm_ZoneVariance | 0 | 0 | 0.5 | 3 | 0 | 0 |
| exponential_ngtdm_Contrast | 0 | 0 | 0.5 | 3 | 0 | 0 |
| logarithm_glcm_JointAverage | 0 | 0 | 0.5 | 3 | 0 | 0 |
| exponential_ngtdm_Busyness | 0 | 0 | 0.5 | 3 | 0 | 0 |
| logarithm_gldm_HighGrayLevelEmphasis | 0 | 0.00043917 | 0.5 | 3 | 0.002516518 | -0.00251652 |
| logarithm_glcm_Idm | 0 | 0 | 0.5 | 3 | 0 | 0 |
| exponential_glszm_LargeAreaEmphasis | 0 | 0 | 0.5 | 3 | 0 | 0 |
| exponential_glszm_HighGrayLevelZoneEmphasis | 0 | 0 | 0.5 | 3 | 0 | 0 |
| logarithm_glrlm_RunLengthNonUniformity | 0 | 0 | 0.5 | 3 | 0 | 0 |
| exponential_glrlm_LongRunLowGrayLevelEmphasis | 0 | 0 | 0.5 | 3 | 0 | 0 |
| logarithm_glcm_DifferenceEntropy | 0 | 0 | 0.5 | 3 | 0 | 0 |
| exponential_glrlm_LowGrayLevelRunEmphasis | 0 | 0 | 0.5 | 3 | 0 | 0 |
| logarithm_glrlm_LongRunEmphasis | 0 | 0 | 0.5 | 3 | 0 | 0 |
| logarithm_glrlm_HighGrayLevelRunEmphasis | 0 | 0 | 0.5 | 3 | 0 | 0 |
| gradient_glcm_ClusterProminence | 0 | 0 | 0.5 | 3 | 0 | 0 |
| gradient_firstorder_Range | 0 | 0.00043917 | 0.5 | 3 | 0.002516518 | -0.00251652 |
| gradient_firstorder_Minimum | 0 | 0 | 0.5 | 3 | 0 | 0 |
| gradient_firstorder_Mean | 0 | 0.00043917 | 0.5 | 3 | 0.002516518 | -0.00251652 |
| logarithm_glcm_Id | 0 | 0 | 0.5 | 3 | 0 | 0 |
| logarithm_glrlm_GrayLevelNonUniformity | 0 | 0.00043917 | 0.5 | 3 | 0.002516518 | -0.00251652 |
| gradient_firstorder_Kurtosis | 0 | 0 | 0.5 | 3 | 0 | 0 |
| exponential_glrlm_RunPercentage | 0 | 0 | 0.5 | 3 | 0 | 0 |
| exponential_glszm_GrayLevelNonUniformity | 0 | 0 | 0.5 | 3 | 0 | 0 |
| exponential_glszm_GrayLevelNonUniformityNormalized | 0 | 0 | 0.5 | 3 | 0 | 0 |
| exponential_glszm_GrayLevelVariance | 0 | 0 | 0.5 | 3 | 0 | 0 |
| gradient_gldm_DependenceNonUniformity | 0 | 0 | 0.5 | 3 | 0 | 0 |
| exponential_glcm_MCC | 0 | 0 | 0.5 | 3 | 0 | 0 |
| original_firstorder_RobustMeanAbsoluteDeviation | 0 | 0 | 0.5 | 3 | 0 | 0 |
| original_glrlm_GrayLevelVariance | 0 | 0 | 0.5 | 3 | 0 | 0 |
| gradient_ngtdm_Busyness | 0 | 0 | 0.5 | 3 | 0 | 0 |
| original_glrlm_LowGrayLevelRunEmphasis | 0 | 0 | 0.5 | 3 | 0 | 0 |
| gradient_ngtdm_Contrast | 0 | 0 | 0.5 | 3 | 0 | 0 |
| exponential_firstorder_Range | 0 | 0.00043917 | 0.5 | 3 | 0.002516518 | -0.00251652 |
| exponential_firstorder_Uniformity | 0 | 0 | 0.5 | 3 | 0 | 0 |
| gradient_ngtdm_Strength | 0 | 0 | 0.5 | 3 | 0 | 0 |
| exponential_glcm_Autocorrelation | 0 | 0 | 0.5 | 3 | 0 | 0 |
| exponential_glcm_ClusterProminence | 0 | 0 | 0.5 | 3 | 0 | 0 |
| gradient_glrlm_LongRunLowGrayLevelEmphasis | 0 | 0 | 0.5 | 3 | 0 | 0 |
| original_glrlm_RunLengthNonUniformityNormalized | 0 | 0 | 0.5 | 3 | 0 | 0 |
| original_glrlm_GrayLevelNonUniformity | 0 | 0 | 0.5 | 3 | 0 | 0 |
| original_gldm_SmallDependenceLowGrayLevelEmphasis | 0 | 0 | 0.5 | 3 | 0 | 0 |
| exponential_glcm_ClusterShade | 0 | 0 | 0.5 | 3 | 0 | 0 |
| exponential_glcm_ClusterTendency | 0 | 0 | 0.5 | 3 | 0 | 0 |
| gradient_gldm_GrayLevelNonUniformity | 0 | 0 | 0.5 | 3 | 0 | 0 |
| original_gldm_LowGrayLevelEmphasis | 0 | 0 | 0.5 | 3 | 0 | 0 |
| exponential_glcm_Correlation | 0 | 0 | 0.5 | 3 | 0 | 0 |
| original_gldm_LargeDependenceEmphasis | 0 | 0 | 0.5 | 3 | 0 | 0 |
| original_ngtdm_Busyness | 0 | 0 | 0.5 | 3 | 0 | 0 |
| original_glrlm_RunPercentage | 0 | 0 | 0.5 | 3 | 0 | 0 |
| exponential_glcm_DifferenceEntropy | 0 | 0 | 0.5 | 3 | 0 | 0 |
| original_glszm_SizeZoneNonUniformity | 0 | 0 | 0.5 | 3 | 0 | 0 |
| air space | 0 | 0 | 0.5 | 3 | 0 | 0 |
| original_glszm_ZoneVariance | 0 | 0 | 0.5 | 3 | 0 | 0 |
| lobulation | 0 | 0 | 0.5 | 3 | 0 | 0 |
| gradient_glszm_LargeAreaHighGrayLevelEmphasis | 0 | 0 | 0.5 | 3 | 0 | 0 |
| original_glszm_SmallAreaLowGrayLevelEmphasis | 0 | 0 | 0.5 | 3 | 0 | 0 |
| gradient_glszm_LargeAreaEmphasis | 0 | 0 | 0.5 | 3 | 0 | 0 |
| gradient_glszm_ZoneEntropy | 0 | 0 | 0.5 | 3 | 0 | 0 |
| calcification | 0 | 0 | 0.5 | 3 | 0 | 0 |
| gradient_glszm_GrayLevelVariance | 0 | 0 | 0.5 | 3 | 0 | 0 |
| exponential_firstorder_Maximum | 0 | 0 | 0.5 | 3 | 0 | 0 |
| original_glszm_LargeAreaLowGrayLevelEmphasis | 0 | 0 | 0.5 | 3 | 0 | 0 |
| original_glszm_LargeAreaEmphasis | 0 | 0 | 0.5 | 3 | 0 | 0 |
| gradient_glszm_ZonePercentage | 0 | 0 | 0.5 | 3 | 0 | 0 |
| exponential_firstorder_Energy | 0 | 0 | 0.5 | 3 | 0 | 0 |
| exponential_firstorder_Entropy | 0 | 0 | 0.5 | 3 | 0 | 0 |
| original_glszm_GrayLevelVariance | 0 | 0 | 0.5 | 3 | 0 | 0 |
| gradient_glszm_ZoneVariance | 0 | 0 | 0.5 | 3 | 0 | 0 |
| exponential_firstorder_InterquartileRange | 0 | 0 | 0.5 | 3 | 0 | 0 |
| exponential_glcm_DifferenceAverage | 0 | 0 | 0.5 | 3 | 0 | 0 |
| exponential_glcm_Contrast | 0 | 0 | 0.5 | 3 | 0 | 0 |
| original_gldm_GrayLevelVariance | 0 | 0 | 0.5 | 3 | 0 | 0 |
| exponential_glcm_Imc1 | 0 | 0 | 0.5 | 3 | 0 | 0 |
| exponential_glcm_Idm | 0 | 0 | 0.5 | 3 | 0 | 0 |
| exponential_glcm_Idmn | 0 | 0 | 0.5 | 3 | 0 | 0 |
| exponential_glcm_Idn | 0 | 0 | 0.5 | 3 | 0 | 0 |
| original_gldm_GrayLevelNonUniformity | 0 | 0 | 0.5 | 3 | 0 | 0 |
| gradient_gldm_LowGrayLevelEmphasis | 0 | 0.00076067 | 0.5 | 3 | 0.004358737 | -0.00435874 |
| original_glcm_DifferenceAverage | 0 | 0.00043917 | 0.5 | 3 | 0.002516518 | -0.00251652 |
| gradient_gldm_LargeDependenceEmphasis | 0 | 0 | 0.5 | 3 | 0 | 0 |
| logarithm_firstorder_Mean | 0 | 0 | 0.5 | 3 | 0 | 0 |
| original_glcm_ClusterTendency | 0 | 0 | 0.5 | 3 | 0 | 0 |
| gradient_glrlm_LongRunEmphasis | 0 | 0 | 0.5 | 3 | 0 | 0 |
| exponential_glcm_Imc2 | 0 | 0 | 0.5 | 3 | 0 | 0 |
| original_glcm_ClusterShade | 0 | 0 | 0.5 | 3 | 0 | 0 |
| exponential_glcm_InverseVariance | 0 | 0 | 0.5 | 3 | 0 | 0 |
| original_glcm_ClusterProminence | 0 | 0 | 0.5 | 3 | 0 | 0 |
| original_firstorder_Variance | 0 | 0 | 0.5 | 3 | 0 | 0 |
| original_firstorder_Uniformity | 0 | 0 | 0.5 | 3 | 0 | 0 |
| original_firstorder_Skewness | 0 | 0 | 0.5 | 3 | 0 | 0 |
| logarithm_firstorder_Median | 0 | 0 | 0.5 | 3 | 0 | 0 |
| original_glcm_Idn | 0 | 0 | 0.5 | 3 | 0 | 0 |
| gradient_gldm_SmallDependenceEmphasis | 0 | 0 | 0.5 | 3 | 0 | 0 |
| exponential_ngtdm_Complexity | 0 | 0 | 0.5 | 3 | 0 | 0 |
| original_gldm_DependenceNonUniformityNormalized | 0 | 0 | 0.5 | 3 | 0 | 0 |
| original_glcm_SumSquares | 0 | 0 | 0.5 | 3 | 0 | 0 |
| logarithm_firstorder_90Percentile | 0 | 0.00043917 | 0.5 | 3 | 0.002516518 | -0.00251652 |
| original_glcm_MaximumProbability | 0 | 0.00076067 | 0.5 | 3 | 0.004358737 | -0.00435874 |
| exponential_glcm_DifferenceVariance | 0 | 0 | 0.5 | 3 | 0 | 0 |
| original_gldm_DependenceNonUniformity | 0 | 0 | 0.5 | 3 | 0 | 0 |
| exponential_glcm_Id | 0 | 0 | 0.5 | 3 | 0 | 0 |
| logarithm_firstorder_10Percentile | 0 | 0 | 0.5 | 3 | 0 | 0 |
| original_glcm_JointEnergy | 0 | 0 | 0.5 | 3 | 0 | 0 |
| original_glcm_Imc2 | 0 | 0 | 0.5 | 3 | 0 | 0 |
| wavelet-LL_firstorder_90Percentile | -3.70E-17 | 0.00043917 | 0.5 | 3 | 0.002516518 | -0.00251652 |
| square_firstorder_Entropy | -3.70E-17 | 0.00043917 | 0.5 | 3 | 0.002516518 | -0.00251652 |
| exponential_firstorder_Kurtosis | -3.70E-17 | 0.00158347 | 0.5 | 3 | 0.009073434 | -0.00907343 |
| gradient_firstorder_InterquartileRange | -3.70E-17 | 0.00076067 | 0.5 | 3 | 0.004358737 | -0.00435874 |
| exponential_glrlm_RunEntropy | -3.70E-17 | 0.00087835 | 0.5 | 3 | 0.005033035 | -0.00503304 |
| original_firstorder_Median | -3.70E-17 | 0.00116195 | 0.5 | 3 | 0.00665808 | -0.00665808 |
| wavelet-HH_glcm_Contrast | -3.70E-17 | 0.00158347 | 0.5 | 3 | 0.009073434 | -0.00907343 |
| original_glszm_SmallAreaEmphasis | -3.70E-17 | 0.00232389 | 0.5 | 3 | 0.01331616 | -0.01331616 |
| wavelet-LH_glcm_Correlation | -7.40E-17 | 0.00304269 | 0.5 | 3 | 0.017434946 | -0.01743495 |
| logarithm_glrlm_LongRunHighGrayLevelEmphasis | -0.00014639 | 0.00067085 | 0.62909945 | 3 | 0.003697653 | -0.00399044 |
| wavelet-HL_firstorder_MeanAbsoluteDeviation | -0.00014639 | 0.00025356 | 0.78867514 | 3 | 0.001306521 | -0.0015993 |
| wavelet-HL_glrlm_LongRunHighGrayLevelEmphasis | -0.00014639 | 0.00091421 | 0.59622505 | 3 | 0.005092158 | -0.00538494 |
| wavelet-HH_glszm_LargeAreaHighGrayLevelEmphasis | -0.00014639 | 0.00067085 | 0.62909945 | 3 | 0.003697653 | -0.00399044 |
| wavelet-HL_firstorder_Energy | -0.00014639 | 0.00025356 | 0.78867514 | 3 | 0.001306521 | -0.0015993 |
| wavelet-HH_glcm_Imc1 | -0.00014639 | 0.00154233 | 0.55773503 | 3 | 0.008691328 | -0.00898411 |
| logarithm_firstorder_Uniformity | -0.00014639 | 0.00025356 | 0.78867514 | 3 | 0.001306521 | -0.0015993 |
| wavelet-HH_gldm_DependenceVariance | -0.00014639 | 0.00025356 | 0.78867514 | 3 | 0.001306521 | -0.0015993 |
| wavelet-HH_glcm_DifferenceVariance | -0.00014639 | 0.00025356 | 0.78867514 | 3 | 0.001306521 | -0.0015993 |
| wavelet-HH_gldm_DependenceNonUniformityNormalized | -0.00014639 | 0.00025356 | 0.78867514 | 3 | 0.001306521 | -0.0015993 |
| gradient_glrlm_LowGrayLevelRunEmphasis | -0.00014639 | 0.00050712 | 0.66666667 | 3 | 0.002759433 | -0.00305222 |
| logarithm_glcm_Idmn | -0.00014639 | 0.00025356 | 0.78867514 | 3 | 0.001306521 | -0.0015993 |
| wavelet-LH_glrlm_GrayLevelNonUniformityNormalized | -0.00014639 | 0.00025356 | 0.78867514 | 3 | 0.001306521 | -0.0015993 |
| squareroot_glszm_SmallAreaEmphasis | -0.00014639 | 0.00050712 | 0.66666667 | 3 | 0.002759433 | -0.00305222 |
| squareroot_gldm_DependenceVariance | -0.00014639 | 0.00025356 | 0.78867514 | 3 | 0.001306521 | -0.0015993 |
| original_shape2D_MaximumDiameter | -0.00014639 | 0.00067085 | 0.62909945 | 3 | 0.003697653 | -0.00399044 |
| wavelet-LL_glszm_ZoneVariance | -0.00014639 | 0.00025356 | 0.78867514 | 3 | 0.001306521 | -0.0015993 |
| wavelet-LL_glcm_JointEntropy | -0.00014639 | 0.00025356 | 0.78867514 | 3 | 0.001306521 | -0.0015993 |
| square_gldm_SmallDependenceEmphasis | -0.00014639 | 0.00025356 | 0.78867514 | 3 | 0.001306521 | -0.0015993 |
| original_glcm_Imc1 | -0.00014639 | 0.00067085 | 0.62909945 | 3 | 0.003697653 | -0.00399044 |
| original_glcm_DifferenceVariance | -0.00014639 | 0.00025356 | 0.78867514 | 3 | 0.001306521 | -0.0015993 |
| original_glcm_Correlation | -0.00014639 | 0.00025356 | 0.78867514 | 3 | 0.001306521 | -0.0015993 |
| original_firstorder_InterquartileRange | -0.00014639 | 0.00025356 | 0.78867514 | 3 | 0.001306521 | -0.0015993 |
| squareroot_firstorder_Uniformity | -0.00014639 | 0.00067085 | 0.62909945 | 3 | 0.003697653 | -0.00399044 |
| wavelet-LH_glrlm_ShortRunEmphasis | -0.00014639 | 0.00025356 | 0.78867514 | 3 | 0.001306521 | -0.0015993 |
| exponential_gldm_SmallDependenceLowGrayLevelEmphasis | -0.00014639 | 0.00050712 | 0.66666667 | 3 | 0.002759433 | -0.00305222 |
| squareroot_glcm_SumSquares | -0.00014639 | 0.00025356 | 0.78867514 | 3 | 0.001306521 | -0.0015993 |
| original_firstorder_90Percentile | -0.00014639 | 0.00141175 | 0.56299408 | 3 | 0.007943081 | -0.00823586 |
| squareroot_glrlm_RunPercentage | -0.00014639 | 0.00025356 | 0.78867514 | 3 | 0.001306521 | -0.0015993 |
| squareroot_gldm_LargeDependenceEmphasis | -0.00014639 | 0.00025356 | 0.78867514 | 3 | 0.001306521 | -0.0015993 |
| squareroot_glrlm_HighGrayLevelRunEmphasis | -0.00014639 | 0.00025356 | 0.78867514 | 3 | 0.001306521 | -0.0015993 |
| logarithm_glcm_JointEnergy | -0.00014639 | 0.00025356 | 0.78867514 | 3 | 0.001306521 | -0.0015993 |
| exponential_glszm_SmallAreaEmphasis | -0.00014639 | 0.00025356 | 0.78867514 | 3 | 0.001306521 | -0.0015993 |
| squareroot_gldm_LargeDependenceHighGrayLevelEmphasis | -0.00014639 | 0.00025356 | 0.78867514 | 3 | 0.001306521 | -0.0015993 |
| wavelet-LL_glcm_MCC | -0.00014639 | 0.0013417 | 0.56622662 | 3 | 0.007541697 | -0.00783448 |
| squareroot_glszm_ZoneEntropy | -0.00014639 | 0.00091421 | 0.59622505 | 3 | 0.005092158 | -0.00538494 |
| exponential_gldm_DependenceVariance | -0.00014639 | 0.00091421 | 0.59622505 | 3 | 0.005092158 | -0.00538494 |
| wavelet-HH_gldm_SmallDependenceHighGrayLevelEmphasis | -0.00014639 | 0.00050712 | 0.66666667 | 3 | 0.002759433 | -0.00305222 |
| wavelet-HH_ngtdm_Strength | -0.00014639 | 0.00110523 | 0.58006408 | 3 | 0.006186706 | -0.00647949 |
| squareroot_firstorder_Minimum | -0.00014639 | 0.00110523 | 0.58006408 | 3 | 0.006186706 | -0.00647949 |
| wavelet-LL_glrlm_RunLengthNonUniformity | -0.00014639 | 0.00067085 | 0.62909945 | 3 | 0.003697653 | -0.00399044 |
| wavelet-LH_gldm_DependenceNonUniformity | -0.00014639 | 0.00050712 | 0.66666667 | 3 | 0.002759433 | -0.00305222 |
| original_glszm_SmallAreaHighGrayLevelEmphasis | -0.00014639 | 0.00067085 | 0.62909945 | 3 | 0.003697653 | -0.00399044 |
| wavelet-HH_firstorder_Entropy | -0.00014639 | 0.00067085 | 0.62909945 | 3 | 0.003697653 | -0.00399044 |
| gradient_glrlm_RunEntropy | -0.00014639 | 0.00067085 | 0.62909945 | 3 | 0.003697653 | -0.00399044 |
| gradient_glszm_GrayLevelNonUniformityNormalized | -0.00029278 | 0.00025356 | 0.90824829 | 3 | 0.001160129 | -0.0017457 |
| logarithm_glcm_SumSquares | -0.00029278 | 0.00025356 | 0.90824829 | 3 | 0.001160129 | -0.0017457 |
| squareroot_glszm_LargeAreaEmphasis | -0.00029278 | 0.00067085 | 0.73570226 | 3 | 0.003551261 | -0.00413683 |
| original_glrlm_GrayLevelNonUniformityNormalized | -0.00029278 | 0.00025356 | 0.90824829 | 3 | 0.001160129 | -0.0017457 |
| square_glcm_Correlation | -0.00029278 | 0.00025356 | 0.90824829 | 3 | 0.001160129 | -0.0017457 |
| wavelet-LL_glrlm_LongRunLowGrayLevelEmphasis | -0.00029278 | 0.00050712 | 0.78867514 | 3 | 0.002613041 | -0.00319861 |
| exponential_firstorder_RobustMeanAbsoluteDeviation | -0.00029278 | 0.00025356 | 0.90824829 | 3 | 0.001160129 | -0.0017457 |
| square_glcm_ClusterProminence | -0.00029278 | 0.00101423 | 0.66666667 | 3 | 0.005518866 | -0.00610443 |
| exponential_firstorder_90Percentile | -0.00029278 | 0.00025356 | 0.90824829 | 3 | 0.001160129 | -0.0017457 |
| square_firstorder_MeanAbsoluteDeviation | -0.00029278 | 0.00025356 | 0.90824829 | 3 | 0.001160129 | -0.0017457 |
| exponential_firstorder_MeanAbsoluteDeviation | -0.00029278 | 0.00025356 | 0.90824829 | 3 | 0.001160129 | -0.0017457 |
| wavelet-HL_firstorder_90Percentile | -0.00029278 | 0.00025356 | 0.90824829 | 3 | 0.001160129 | -0.0017457 |
| gradient_gldm_SmallDependenceHighGrayLevelEmphasis | -0.00029278 | 0.00025356 | 0.90824829 | 3 | 0.001160129 | -0.0017457 |
| squareroot_glrlm_RunVariance | -0.00029278 | 0.00025356 | 0.90824829 | 3 | 0.001160129 | -0.0017457 |
| gradient_glszm_SizeZoneNonUniformityNormalized | -0.00029278 | 0.00025356 | 0.90824829 | 3 | 0.001160129 | -0.0017457 |
| original_firstorder_Minimum | -0.00029278 | 0.00050712 | 0.78867514 | 3 | 0.002613041 | -0.00319861 |
| squareroot_glcm_Autocorrelation | -0.00029278 | 0.00025356 | 0.90824829 | 3 | 0.001160129 | -0.0017457 |
| squareroot_glcm_Id | -0.00029278 | 0.00025356 | 0.90824829 | 3 | 0.001160129 | -0.0017457 |
| wavelet-HH_glrlm_ShortRunHighGrayLevelEmphasis | -0.00029278 | 0.00067085 | 0.73570226 | 3 | 0.003551261 | -0.00413683 |
| wavelet-LL_firstorder_RootMeanSquared | -0.00029278 | 0.00025356 | 0.90824829 | 3 | 0.001160129 | -0.0017457 |
| wavelet-LH_gldm_SmallDependenceLowGrayLevelEmphasis | -0.00029278 | 0.00025356 | 0.90824829 | 3 | 0.001160129 | -0.0017457 |
| exponential_glrlm_RunLengthNonUniformityNormalized | -0.00029278 | 0.00067085 | 0.73570226 | 3 | 0.003551261 | -0.00413683 |
| wavelet-LH_glszm_GrayLevelNonUniformity | -0.00029278 | 0.00067085 | 0.73570226 | 3 | 0.003551261 | -0.00413683 |
| original_firstorder_RootMeanSquared | -0.00029278 | 0.00025356 | 0.90824829 | 3 | 0.001160129 | -0.0017457 |
| squareroot_glcm_JointEnergy | -0.00029278 | 0.00025356 | 0.90824829 | 3 | 0.001160129 | -0.0017457 |
| wavelet-LH_gldm_LargeDependenceEmphasis | -0.00029278 | 0.00025356 | 0.90824829 | 3 | 0.001160129 | -0.0017457 |
| wavelet-LH_glcm_JointEntropy | -0.00029278 | 0.00025356 | 0.90824829 | 3 | 0.001160129 | -0.0017457 |
| original_glcm_DifferenceEntropy | -0.00029278 | 0.00025356 | 0.90824829 | 3 | 0.001160129 | -0.0017457 |
| wavelet-LL_glszm_LowGrayLevelZoneEmphasis | -0.00029278 | 0.00091421 | 0.68257419 | 3 | 0.004945767 | -0.00553133 |
| original_glszm_ZoneEntropy | -0.00029278 | 0.00241878 | 0.57332356 | 3 | 0.013567116 | -0.01415268 |
| wavelet-HL_glrlm_LongRunEmphasis | -0.00043917 | 0.00043917 | 0.88729834 | 3 | 0.002077343 | -0.00295569 |
| gradient_glszm_GrayLevelNonUniformity | -0.00043917 | 0.00043917 | 0.88729834 | 3 | 0.002077343 | -0.00295569 |
| wavelet-HL_glcm_Contrast | -0.00043917 | 0.00076067 | 0.78867514 | 3 | 0.003919562 | -0.00479791 |
| wavelet-LH_glszm_SizeZoneNonUniformity | -0.00043917 | 0.00043917 | 0.88729834 | 3 | 0.002077343 | -0.00295569 |
| wavelet-LH_glszm_HighGrayLevelZoneEmphasis | -0.00043917 | 0.00043917 | 0.88729834 | 3 | 0.002077343 | -0.00295569 |
| gradient_firstorder_Uniformity | -0.00043917 | 0 | 0.5 | 3 | -0.00043917 | -0.00043917 |
| wavelet-LH_ngtdm_Coarseness | -0.00043917 | 0.00043917 | 0.88729834 | 3 | 0.002077343 | -0.00295569 |
| wavelet-LL_firstorder_Range | -0.00043917 | 0.00043917 | 0.88729834 | 3 | 0.002077343 | -0.00295569 |
| gradient_glcm_SumEntropy | -0.00043917 | 0.00158347 | 0.66081688 | 3 | 0.008634259 | -0.00951261 |
| wavelet-LL_glcm_SumEntropy | -0.00043917 | 0 | 0.5 | 3 | -0.00043917 | -0.00043917 |
| wavelet-LL_glcm_Correlation | -0.00043917 | 0.00043917 | 0.88729834 | 3 | 0.002077343 | -0.00295569 |
| wavelet-LL_glcm_Imc2 | -0.00043917 | 0 | 0.5 | 3 | -0.00043917 | -0.00043917 |
| wavelet-LL_glcm_Id | -0.00043917 | 0 | 0.5 | 3 | -0.00043917 | -0.00043917 |
| gradient_glrlm_LongRunHighGrayLevelEmphasis | -0.00043917 | 0.00043917 | 0.88729834 | 3 | 0.002077343 | -0.00295569 |
| gradient_firstorder_RootMeanSquared | -0.00043917 | 0 | 0.5 | 3 | -0.00043917 | -0.00043917 |
| square_glszm_LowGrayLevelZoneEmphasis | -0.00043917 | 0.00087835 | 0.76111648 | 3 | 0.004593861 | -0.00547221 |
| original_gldm_SmallDependenceEmphasis | -0.00043917 | 0.00043917 | 0.88729834 | 3 | 0.002077343 | -0.00295569 |
| gradient_ngtdm_Coarseness | -0.00043917 | 0.00043917 | 0.88729834 | 3 | 0.002077343 | -0.00295569 |
| squareroot_glrlm_GrayLevelVariance | -0.00043917 | 0.00076067 | 0.78867514 | 3 | 0.003919562 | -0.00479791 |
| original_gldm_DependenceEntropy | -0.00043917 | 0.00076067 | 0.78867514 | 3 | 0.003919562 | -0.00479791 |
| squareroot_glcm_Contrast | -0.00043917 | 0.00076067 | 0.78867514 | 3 | 0.003919562 | -0.00479791 |
| square_glcm_Idn | -0.00043917 | 0 | 0.5 | 3 | -0.00043917 | -0.00043917 |
| logarithm_gldm_DependenceNonUniformity | -0.00043917 | 0.00043917 | 0.88729834 | 3 | 0.002077343 | -0.00295569 |
| wavelet-HH_glcm_Idm | -0.00043917 | 0 | 0.5 | 3 | -0.00043917 | -0.00043917 |
| squareroot_glrlm_ShortRunHighGrayLevelEmphasis | -0.00043917 | 0.00087835 | 0.76111648 | 3 | 0.004593861 | -0.00547221 |
| logarithm_firstorder_Skewness | -0.00043917 | 0.00043917 | 0.88729834 | 3 | 0.002077343 | -0.00295569 |
| logarithm_gldm_LargeDependenceHighGrayLevelEmphasis | -0.00043917 | 0 | 0.5 | 3 | -0.00043917 | -0.00043917 |
| squareroot_glszm_LargeAreaLowGrayLevelEmphasis | -0.00043917 | 0.00087835 | 0.76111648 | 3 | 0.004593861 | -0.00547221 |
| wavelet-HH_glcm_InverseVariance | -0.00043917 | 0.00116195 | 0.71004201 | 3 | 0.006218906 | -0.00709725 |
| original_glcm_Idmn | -0.00043917 | 0 | 0.5 | 3 | -0.00043917 | -0.00043917 |
| squareroot_ngtdm_Busyness | -0.00043917 | 0.00043917 | 0.88729834 | 3 | 0.002077343 | -0.00295569 |
| squareroot_firstorder_Maximum | -0.00043917 | 0.00087835 | 0.76111648 | 3 | 0.004593861 | -0.00547221 |
| square_glrlm_RunVariance | -0.00043917 | 0.00043917 | 0.88729834 | 3 | 0.002077343 | -0.00295569 |
| squareroot_firstorder_Entropy | -0.00043917 | 0.00087835 | 0.76111648 | 3 | 0.004593861 | -0.00547221 |
| logarithm_firstorder_InterquartileRange | -0.00043917 | 0.00043917 | 0.88729834 | 3 | 0.002077343 | -0.00295569 |
| original_ngtdm_Contrast | -0.00043917 | 0.00087835 | 0.76111648 | 3 | 0.004593861 | -0.00547221 |
| squareroot_firstorder_Skewness | -0.00043917 | 0.00087835 | 0.76111648 | 3 | 0.004593861 | -0.00547221 |
| square_gldm_LargeDependenceEmphasis | -0.00043917 | 0.00116195 | 0.71004201 | 3 | 0.006218906 | -0.00709725 |
| gradient_glcm_ClusterShade | -0.00043917 | 0.00116195 | 0.71004201 | 3 | 0.006218906 | -0.00709725 |
| logarithm_glrlm_RunEntropy | -0.00043917 | 0.00116195 | 0.71004201 | 3 | 0.006218906 | -0.00709725 |
| squareroot_glszm_SmallAreaHighGrayLevelEmphasis | -0.00043917 | 0.00131752 | 0.68898224 | 3 | 0.007110379 | -0.00798873 |
| wavelet-HL_glszm_ZoneEntropy | -0.00043917 | 0.00116195 | 0.71004201 | 3 | 0.006218906 | -0.00709725 |
| wavelet-HH_firstorder_Kurtosis | -0.00043917 | 0.00191432 | 0.63525045 | 3 | 0.010530072 | -0.01140842 |
| original_glszm_SizeZoneNonUniformityNormalized | -0.00058557 | 0.00141175 | 0.72645541 | 3 | 0.007503907 | -0.00867504 |
| wavelet-HL_glrlm_LowGrayLevelRunEmphasis | -0.00058557 | 0.00050712 | 0.90824829 | 3 | 0.002320259 | -0.00349139 |
| wavelet-HL_glcm_MaximumProbability | -0.00058557 | 0.00025356 | 0.97140452 | 3 | 0.000867346 | -0.00203848 |
| logarithm_firstorder_Variance | -0.00058557 | 0.00025356 | 0.97140452 | 3 | 0.000867346 | -0.00203848 |
| squareroot_firstorder_RobustMeanAbsoluteDeviation | -0.00058557 | 0.00067085 | 0.86514837 | 3 | 0.003258479 | -0.00442961 |
| wavelet-HL_gldm_LargeDependenceHighGrayLevelEmphasis | -0.00058557 | 0.00091421 | 0.8086067 | 3 | 0.004652984 | -0.00582412 |
| wavelet-LH_glszm_SizeZoneNonUniformityNormalized | -0.00058557 | 0.00067085 | 0.86514837 | 3 | 0.003258479 | -0.00442961 |
| squareroot_firstorder_InterquartileRange | -0.00058557 | 0.00025356 | 0.97140452 | 3 | 0.000867346 | -0.00203848 |
| gradient_firstorder_MeanAbsoluteDeviation | -0.00058557 | 0.00050712 | 0.90824829 | 3 | 0.002320259 | -0.00349139 |
| wavelet-LL_firstorder_Kurtosis | -0.00058557 | 0.00067085 | 0.86514837 | 3 | 0.003258479 | -0.00442961 |
| wavelet-HL_glszm_GrayLevelVariance | -0.00058557 | 0.00198035 | 0.67025131 | 3 | 0.010762041 | -0.01193317 |
| gradient_glrlm_GrayLevelNonUniformity | -0.00058557 | 0.00067085 | 0.86514837 | 3 | 0.003258479 | -0.00442961 |
| original_ngtdm_Coarseness | -0.00058557 | 0.00050712 | 0.90824829 | 3 | 0.002320259 | -0.00349139 |
| logarithm_ngtdm_Contrast | -0.00058557 | 0.00110523 | 0.77216553 | 3 | 0.005747532 | -0.00691866 |
| wavelet-LL_glcm_MaximumProbability | -0.00058557 | 0.00067085 | 0.86514837 | 3 | 0.003258479 | -0.00442961 |
| wavelet-LL_ngtdm_Contrast | -0.00058557 | 0.00067085 | 0.86514837 | 3 | 0.003258479 | -0.00442961 |
| gradient_glszm_LargeAreaLowGrayLevelEmphasis | -0.00058557 | 0.00050712 | 0.90824829 | 3 | 0.002320259 | -0.00349139 |
| necrosis | -0.00058557 | 0.00067085 | 0.86514837 | 3 | 0.003258479 | -0.00442961 |
| wavelet-HH_glszm_SizeZoneNonUniformityNormalized | -0.00058557 | 0.00110523 | 0.77216553 | 3 | 0.005747532 | -0.00691866 |
| wavelet-LL_glszm_GrayLevelNonUniformity | -0.00058557 | 0.00050712 | 0.90824829 | 3 | 0.002320259 | -0.00349139 |
| square_glcm_ClusterShade | -0.00058557 | 0.00050712 | 0.90824829 | 3 | 0.002320259 | -0.00349139 |
| square_glszm_ZoneEntropy | -0.00058557 | 0.00067085 | 0.86514837 | 3 | 0.003258479 | -0.00442961 |
| gradient_glrlm_RunPercentage | -0.00058557 | 0.00025356 | 0.97140452 | 3 | 0.000867346 | -0.00203848 |
| wavelet-LL_gldm_DependenceVariance | -0.00058557 | 0.00025356 | 0.97140452 | 3 | 0.000867346 | -0.00203848 |
| square_glcm_DifferenceEntropy | -0.00058557 | 0.00166269 | 0.69802951 | 3 | 0.008941817 | -0.01011295 |
| original_firstorder_Range | -0.00058557 | 0.00025356 | 0.97140452 | 3 | 0.000867346 | -0.00203848 |
| wavelet-HH_gldm_HighGrayLevelEmphasis | -0.00058557 | 0.00025356 | 0.97140452 | 3 | 0.000867346 | -0.00203848 |
| wavelet-LL_glcm_ClusterProminence | -0.00058557 | 0.00067085 | 0.86514837 | 3 | 0.003258479 | -0.00442961 |
| original_shape2D_MinorAxisLength | -0.00058557 | 0.00126779 | 0.74618298 | 3 | 0.006678995 | -0.00785013 |
| wavelet-HH_glrlm_LowGrayLevelRunEmphasis | -0.00058557 | 0.00091421 | 0.8086067 | 3 | 0.004652984 | -0.00582412 |
| logarithm_glcm_SumEntropy | -0.00073196 | 0.0013417 | 0.77777778 | 3 | 0.006956131 | -0.00842005 |
| logarithm_firstorder_RootMeanSquared | -0.00073196 | 0.00207546 | 0.6982629 | 3 | 0.011160642 | -0.01262456 |
| square_gldm_DependenceNonUniformityNormalized | -0.00073196 | 0.0013417 | 0.77777778 | 3 | 0.006956131 | -0.00842005 |
| original_glcm_Idm | -0.00073196 | 0.00025356 | 0.98112522 | 3 | 0.000720955 | -0.00218487 |
| square_glcm_DifferenceAverage | -0.00073196 | 0.00025356 | 0.98112522 | 3 | 0.000720955 | -0.00218487 |
| square_glszm_SmallAreaEmphasis | -0.00073196 | 0.00050712 | 0.93519414 | 3 | 0.002173867 | -0.00363778 |
| original_glszm_ZonePercentage | -0.00073196 | 0.00025356 | 0.98112522 | 3 | 0.000720955 | -0.00218487 |
| wavelet-LL_ngtdm_Busyness | -0.00073196 | 0.00091421 | 0.85007002 | 3 | 0.004506592 | -0.00597051 |
| original_glcm_SumEntropy | -0.00073196 | 0.00025356 | 0.98112522 | 3 | 0.000720955 | -0.00218487 |
| square_glcm_JointEntropy | -0.00073196 | 0.00025356 | 0.98112522 | 3 | 0.000720955 | -0.00218487 |
| gradient_firstorder_Energy | -0.00073196 | 0.00025356 | 0.98112522 | 3 | 0.000720955 | -0.00218487 |
| logarithm_glszm_ZoneVariance | -0.00073196 | 0.00050712 | 0.93519414 | 3 | 0.002173867 | -0.00363778 |
| squareroot_firstorder_90Percentile | -0.00073196 | 0.00025356 | 0.98112522 | 3 | 0.000720955 | -0.00218487 |
| vascular convergence sign | -0.00073196 | 0.00101423 | 0.83113309 | 3 | 0.005079692 | -0.00654361 |
| logarithm_glcm_MaximumProbability | -0.00073196 | 0.00025356 | 0.98112522 | 3 | 0.000720955 | -0.00218487 |
| wavelet-LH_glcm_SumEntropy | -0.00073196 | 0.00101423 | 0.83113309 | 3 | 0.005079692 | -0.00654361 |
| wavelet-LL_glcm_InverseVariance | -0.00073196 | 0.00067085 | 0.90032039 | 3 | 0.003112087 | -0.004576 |
| wavelet-LH_glrlm_RunPercentage | -0.00073196 | 0.00025356 | 0.98112522 | 3 | 0.000720955 | -0.00218487 |
| squareroot_glrlm_ShortRunLowGrayLevelEmphasis | -0.00073196 | 0.00025356 | 0.98112522 | 3 | 0.000720955 | -0.00218487 |
| original_glcm_Contrast | -0.00073196 | 0.00141175 | 0.76802813 | 3 | 0.007357515 | -0.00882143 |
| wavelet-LL_gldm_HighGrayLevelEmphasis | -0.00073196 | 0.00101423 | 0.83113309 | 3 | 0.005079692 | -0.00654361 |
| logarithm_gldm_DependenceEntropy | -0.00073196 | 0.0013417 | 0.77777778 | 3 | 0.006956131 | -0.00842005 |
| square_ngtdm_Contrast | -0.00073196 | 0.00110523 | 0.81497039 | 3 | 0.00560114 | -0.00706506 |
| logarithm_glrlm_GrayLevelNonUniformityNormalized | -0.00073196 | 0.00110523 | 0.81497039 | 3 | 0.00560114 | -0.00706506 |
| wavelet-HH_glszm_ZoneEntropy | -0.00073196 | 0.00025356 | 0.98112522 | 3 | 0.000720955 | -0.00218487 |
| gradient_glrlm_GrayLevelNonUniformityNormalized | -0.00073196 | 0.00050712 | 0.93519414 | 3 | 0.002173867 | -0.00363778 |
| wavelet-HL_glcm_Correlation | -0.00073196 | 0.00025356 | 0.98112522 | 3 | 0.000720955 | -0.00218487 |
| wavelet-HL_firstorder_10Percentile | -0.00073196 | 0.00025356 | 0.98112522 | 3 | 0.000720955 | -0.00218487 |
| logarithm_firstorder_Energy | -0.00073196 | 0.00091421 | 0.85007002 | 3 | 0.004506592 | -0.00597051 |
| wavelet-HH_glszm_HighGrayLevelZoneEmphasis | -0.00073196 | 0.00025356 | 0.98112522 | 3 | 0.000720955 | -0.00218487 |
| wavelet-HL_glcm_Imc2 | -0.00073196 | 0.00025356 | 0.98112522 | 3 | 0.000720955 | -0.00218487 |
| gradient_glrlm_HighGrayLevelRunEmphasis | -0.00073196 | 0.00050712 | 0.93519414 | 3 | 0.002173867 | -0.00363778 |
| original_firstorder_Mean | -0.00073196 | 0.00141175 | 0.76802813 | 3 | 0.007357515 | -0.00882143 |
| squareroot_firstorder_Variance | -0.00073196 | 0.00101423 | 0.83113309 | 3 | 0.005079692 | -0.00654361 |
| wavelet-LL_gldm_GrayLevelNonUniformity | -0.00087835 | 0.00087835 | 0.88729834 | 3 | 0.004154687 | -0.00591138 |
| wavelet-LH_firstorder_Minimum | -0.00087835 | 0.00116195 | 0.83968311 | 3 | 0.005779731 | -0.00753643 |
| squareroot_glszm_GrayLevelVariance | -0.00087835 | 0.0017567 | 0.76111648 | 3 | 0.009187722 | -0.01094442 |
| wavelet-LL_glszm_LargeAreaLowGrayLevelEmphasis | -0.00087835 | 0.00131752 | 0.81622777 | 3 | 0.006671205 | -0.0084279 |
| wavelet-HL_gldm_SmallDependenceEmphasis | -0.00087835 | 0.00043917 | 0.96291005 | 3 | 0.001638169 | -0.00339487 |
| original_glszm_HighGrayLevelZoneEmphasis | -0.00087835 | 0.00076067 | 0.90824829 | 3 | 0.003480388 | -0.00523709 |
| wavelet-HL_glrlm_GrayLevelNonUniformity | -0.00087835 | 0.00076067 | 0.90824829 | 3 | 0.003480388 | -0.00523709 |
| original_glrlm_ShortRunHighGrayLevelEmphasis | -0.00087835 | 0.00087835 | 0.88729834 | 3 | 0.004154687 | -0.00591138 |
| wavelet-HH_glszm_GrayLevelNonUniformityNormalized | -0.00087835 | 0.0017567 | 0.76111648 | 3 | 0.009187722 | -0.01094442 |
| wavelet-HL_glszm_LargeAreaLowGrayLevelEmphasis | -0.00087835 | 0 | 0.5 | 3 | -0.00087835 | -0.00087835 |
| wavelet-HL_glrlm_RunVariance | -0.00087835 | 0 | 0.5 | 3 | -0.00087835 | -0.00087835 |
| gradient_firstorder_Variance | -0.00087835 | 0.00043917 | 0.96291005 | 3 | 0.001638169 | -0.00339487 |
| original_glrlm_RunVariance | -0.00087835 | 0.00076067 | 0.90824829 | 3 | 0.003480388 | -0.00523709 |
| wavelet-LL_glrlm_RunPercentage | -0.00087835 | 0.00043917 | 0.96291005 | 3 | 0.001638169 | -0.00339487 |
| wavelet-HL_gldm_LargeDependenceEmphasis | -0.00087835 | 0.00043917 | 0.96291005 | 3 | 0.001638169 | -0.00339487 |
| square_glrlm_RunEntropy | -0.00087835 | 0.00043917 | 0.96291005 | 3 | 0.001638169 | -0.00339487 |
| wavelet-HL_gldm_LargeDependenceLowGrayLevelEmphasis | -0.00087835 | 0.00116195 | 0.83968311 | 3 | 0.005779731 | -0.00753643 |
| wavelet-LL_glrlm_RunLengthNonUniformityNormalized | -0.00087835 | 0 | 0.5 | 3 | -0.00087835 | -0.00087835 |
| squareroot_firstorder_Energy | -0.00087835 | 0.00043917 | 0.96291005 | 3 | 0.001638169 | -0.00339487 |
| gradient_glszm_SmallAreaEmphasis | -0.00087835 | 0 | 0.5 | 3 | -0.00087835 | -0.00087835 |
| wavelet-HL_glcm_JointEntropy | -0.00087835 | 0.00076067 | 0.90824829 | 3 | 0.003480388 | -0.00523709 |
| squareroot_glrlm_GrayLevelNonUniformity | -0.00087835 | 0.00076067 | 0.90824829 | 3 | 0.003480388 | -0.00523709 |
| wavelet-LH_ngtdm_Strength | -0.00087835 | 0.00043917 | 0.96291005 | 3 | 0.001638169 | -0.00339487 |
| square_firstorder_10Percentile | -0.00087835 | 0.00076067 | 0.90824829 | 3 | 0.003480388 | -0.00523709 |
| squareroot_glcm_JointEntropy | -0.00087835 | 0.00076067 | 0.90824829 | 3 | 0.003480388 | -0.00523709 |
| exponential_gldm_DependenceNonUniformityNormalized | -0.00087835 | 0 | 0.5 | 3 | -0.00087835 | -0.00087835 |
| original_firstorder_Kurtosis | -0.00087835 | 0.00043917 | 0.96291005 | 3 | 0.001638169 | -0.00339487 |
| original_glcm_MCC | -0.00087835 | 0.00087835 | 0.88729834 | 3 | 0.004154687 | -0.00591138 |
| wavelet-LH_gldm_GrayLevelNonUniformity | -0.00087835 | 0.00043917 | 0.96291005 | 3 | 0.001638169 | -0.00339487 |
| wavelet-HL_firstorder_Variance | -0.00087835 | 0.00116195 | 0.83968311 | 3 | 0.005779731 | -0.00753643 |
| logarithm_glszm_ZoneEntropy | -0.00087835 | 0 | 0.5 | 3 | -0.00087835 | -0.00087835 |
| wavelet-LH_glcm_JointAverage | -0.00087835 | 0.00043917 | 0.96291005 | 3 | 0.001638169 | -0.00339487 |
| square_ngtdm_Complexity | -0.00087835 | 0.00076067 | 0.90824829 | 3 | 0.003480388 | -0.00523709 |
| wavelet-HH_gldm_LargeDependenceLowGrayLevelEmphasis | -0.00087835 | 0 | 0.5 | 3 | -0.00087835 | -0.00087835 |
| original_glrlm_ShortRunEmphasis | -0.00087835 | 0.00076067 | 0.90824829 | 3 | 0.003480388 | -0.00523709 |
| wavelet-LL_glrlm_LowGrayLevelRunEmphasis | -0.00087835 | 0.00232389 | 0.71004201 | 3 | 0.012437812 | -0.01419451 |
| wavelet-HH_glcm_Idn | -0.00087835 | 0.00191432 | 0.74494897 | 3 | 0.010090898 | -0.0118476 |
| wavelet-LH_glcm_MCC | -0.00102474 | 0.00050712 | 0.96358633 | 3 | 0.001881084 | -0.00393057 |
| wavelet-HL_glszm_GrayLevelNonUniformityNormalized | -0.00102474 | 0.00067085 | 0.94095855 | 3 | 0.002819304 | -0.00486878 |
| logarithm_glcm_Contrast | -0.00102474 | 0.0017749 | 0.78867514 | 3 | 0.009145645 | -0.01119513 |
| wavelet-HH_glrlm_LongRunEmphasis | -0.00102474 | 0.0013417 | 0.84156503 | 3 | 0.006663349 | -0.00871283 |
| gradient_glcm_Contrast | -0.00102474 | 0.00091421 | 0.90414519 | 3 | 0.004213809 | -0.00626329 |
| logarithm_glcm_DifferenceVariance | -0.00102474 | 0.00141175 | 0.8322053 | 3 | 0.007064733 | -0.00911421 |
| wavelet-HL_glszm_SizeZoneNonUniformityNormalized | -0.00102474 | 0.00025356 | 0.99009803 | 3 | 0.000428172 | -0.00247765 |
| wavelet-LH_glrlm_ShortRunLowGrayLevelEmphasis | -0.00102474 | 0.00154233 | 0.81558437 | 3 | 0.00781298 | -0.00986246 |
| square_firstorder_InterquartileRange | -0.00102474 | 0.00110523 | 0.87523939 | 3 | 0.005308357 | -0.00735784 |
| squareroot_glcm_DifferenceVariance | -0.00102474 | 0.0013417 | 0.84156503 | 3 | 0.006663349 | -0.00871283 |
| wavelet-LH_glrlm_LongRunEmphasis | -0.00102474 | 0.00050712 | 0.96358633 | 3 | 0.001881084 | -0.00393057 |
| wavelet-LL_glszm_ZoneEntropy | -0.00102474 | 0.00025356 | 0.99009803 | 3 | 0.000428172 | -0.00247765 |
| squareroot_glszm_ZoneVariance | -0.00102474 | 0.00067085 | 0.94095855 | 3 | 0.002819304 | -0.00486878 |
| logarithm_firstorder_Entropy | -0.00102474 | 0.00025356 | 0.99009803 | 3 | 0.000428172 | -0.00247765 |
| wavelet-HL_firstorder_Median | -0.00102474 | 0.0013417 | 0.84156503 | 3 | 0.006663349 | -0.00871283 |
| wavelet-LL_glcm_Idmn | -0.00102474 | 0.00091421 | 0.90414519 | 3 | 0.004213809 | -0.00626329 |
| wavelet-LH_gldm_DependenceEntropy | -0.00102474 | 0.00025356 | 0.99009803 | 3 | 0.000428172 | -0.00247765 |
| logarithm_glszm_LargeAreaEmphasis | -0.00102474 | 0.00050712 | 0.96358633 | 3 | 0.001881084 | -0.00393057 |
| squareroot_glrlm_RunEntropy | -0.00102474 | 0.00050712 | 0.96358633 | 3 | 0.001881084 | -0.00393057 |
| wavelet-LH_glrlm_RunEntropy | -0.00102474 | 0.00126779 | 0.85176324 | 3 | 0.006239821 | -0.0082893 |
| square_glrlm_RunLengthNonUniformityNormalized | -0.00102474 | 0.00025356 | 0.99009803 | 3 | 0.000428172 | -0.00247765 |
| wavelet-HL_glcm_Idn | -0.00102474 | 0.00329625 | 0.677915 | 3 | 0.017863118 | -0.0199126 |
| wavelet-LL_glrlm_ShortRunLowGrayLevelEmphasis | -0.00117113 | 0.00101423 | 0.90824829 | 3 | 0.004640517 | -0.00698278 |
| wavelet-LL_firstorder_Entropy | -0.00117113 | 0.0013417 | 0.86514837 | 3 | 0.006516957 | -0.00885922 |
| wavelet-LH_glszm_LargeAreaLowGrayLevelEmphasis | -0.00117113 | 0.0013417 | 0.86514837 | 3 | 0.006516957 | -0.00885922 |
| wavelet-LL_glszm_SizeZoneNonUniformityNormalized | -0.00117113 | 0.00050712 | 0.97140452 | 3 | 0.001734693 | -0.00407696 |
| wavelet-HL_gldm_DependenceNonUniformityNormalized | -0.00117113 | 0.00067085 | 0.95291081 | 3 | 0.002672913 | -0.00501518 |
| wavelet-HH_glrlm_RunPercentage | -0.00117113 | 0.00101423 | 0.90824829 | 3 | 0.004640517 | -0.00698278 |
| wavelet-LL_firstorder_InterquartileRange | -0.00117113 | 0.00110523 | 0.89605902 | 3 | 0.005161966 | -0.00750423 |
| original_firstorder_10Percentile | -0.00117113 | 0.00025356 | 0.99236596 | 3 | 0.000281781 | -0.00262404 |
| wavelet-HL_glszm_SizeZoneNonUniformity | -0.00117113 | 0.00110523 | 0.89605902 | 3 | 0.005161966 | -0.00750423 |
| logarithm_ngtdm_Coarseness | -0.00117113 | 0.00067085 | 0.95291081 | 3 | 0.002672913 | -0.00501518 |
| wavelet-HH_gldm_LargeDependenceEmphasis | -0.00117113 | 0.00025356 | 0.99236596 | 3 | 0.000281781 | -0.00262404 |
| gradient_gldm_LargeDependenceLowGrayLevelEmphasis | -0.00117113 | 0.00067085 | 0.95291081 | 3 | 0.002672913 | -0.00501518 |
| gradient_firstorder_RobustMeanAbsoluteDeviation | -0.00117113 | 0.00067085 | 0.95291081 | 3 | 0.002672913 | -0.00501518 |
| wavelet-HL_firstorder_Maximum | -0.00117113 | 0.00101423 | 0.90824829 | 3 | 0.004640517 | -0.00698278 |
| logarithm_firstorder_Kurtosis | -0.00131752 | 6.41E-17 | 1 | 3 | -0.00131752 | -0.00131752 |
| wavelet-HL_firstorder_Minimum | -0.00131752 | 0.00076067 | 0.95226702 | 3 | 0.003041214 | -0.00567626 |
| wavelet-LH_glcm_Autocorrelation | -0.00131752 | 0.00116195 | 0.90575134 | 3 | 0.005340557 | -0.0079756 |
| wavelet-LH_glcm_Id | -0.00131752 | 0.00043917 | 0.98245064 | 3 | 0.001198995 | -0.00383404 |
| logarithm_gldm_LargeDependenceEmphasis | -0.00131752 | 0.00131752 | 0.88729834 | 3 | 0.00623203 | -0.00886708 |
| logarithm_glcm_MCC | -0.00131752 | 0.00287986 | 0.74440645 | 3 | 0.015184387 | -0.01781943 |
| pleural effusion | -0.00131752 | 0.00116195 | 0.90575134 | 3 | 0.005340557 | -0.0079756 |
| multiple pulmonary metastases | -0.00131752 | 0.00158347 | 0.85687321 | 3 | 0.007755911 | -0.01039096 |
| wavelet-LL_gldm_DependenceNonUniformity | -0.00131752 | 0.0017567 | 0.83824071 | 3 | 0.008748548 | -0.01138359 |
| wavelet-HL_firstorder_Skewness | -0.00131752 | 0.00076067 | 0.95226702 | 3 | 0.003041214 | -0.00567626 |
| original_firstorder_Maximum | -0.00131752 | 0.00043917 | 0.98245064 | 3 | 0.001198995 | -0.00383404 |
| wavelet-HH_glrlm_GrayLevelNonUniformityNormalized | -0.00131752 | 0 | 0.5 | 3 | -0.00131752 | -0.00131752 |
| wavelet-HH_ngtdm_Complexity | -0.00131752 | 0.00116195 | 0.90575134 | 3 | 0.005340557 | -0.0079756 |
| wavelet-LH_firstorder_Energy | -0.00131752 | 0.00087835 | 0.93915503 | 3 | 0.003715512 | -0.00635056 |
| squareroot_glszm_GrayLevelNonUniformityNormalized | -0.00131752 | 0.00076067 | 0.95226702 | 3 | 0.003041214 | -0.00567626 |
| exponential_firstorder_Median | -0.00131752 | 0.00152135 | 0.86380344 | 3 | 0.00739995 | -0.010035 |
| wavelet-LL_glszm_SmallAreaLowGrayLevelEmphasis | -0.00146392 | 0.0017749 | 0.85533453 | 3 | 0.008706471 | -0.0116343 |
| wavelet-HL_glcm_InverseVariance | -0.00146392 | 0.0021664 | 0.81878836 | 3 | 0.010949773 | -0.0138776 |
| logarithm_glcm_ClusterProminence | -0.00146392 | 0.00101423 | 0.93519414 | 3 | 0.004347734 | -0.00727556 |
| logarithm_gldm_SmallDependenceLowGrayLevelEmphasis | -0.00146392 | 0.00141175 | 0.8928371 | 3 | 0.006625558 | -0.00955339 |
| wavelet-LL_gldm_SmallDependenceLowGrayLevelEmphasis | -0.00146392 | 0.00126779 | 0.90824829 | 3 | 0.005800646 | -0.00872848 |
| wavelet-HH_glrlm_RunEntropy | -0.00146392 | 0.00091421 | 0.9454354 | 3 | 0.003774635 | -0.00670246 |
| logarithm_glszm_ZonePercentage | -0.00146392 | 0.00091421 | 0.9454354 | 3 | 0.003774635 | -0.00670246 |
| gradient_glszm_SmallAreaLowGrayLevelEmphasis | -0.00146392 | 0.0017749 | 0.85533453 | 3 | 0.008706471 | -0.0116343 |
| wavelet-LH_glrlm_LongRunHighGrayLevelEmphasis | -0.00146392 | 0.0013417 | 0.90032039 | 3 | 0.006224174 | -0.009152 |
| wavelet-HH_firstorder_InterquartileRange | -0.00146392 | 0.00025356 | 0.99507377 | 3 | -1.10E-05 | -0.00291683 |
| wavelet-LL_firstorder_10Percentile | -0.00146392 | 0.00154233 | 0.87904902 | 3 | 0.007373805 | -0.01030163 |
| square_glszm_GrayLevelNonUniformityNormalized | -0.00146392 | 0.00050712 | 0.98112522 | 3 | 0.00144191 | -0.00436974 |
| square_firstorder_Kurtosis | -0.00146392 | 0.00050712 | 0.98112522 | 3 | 0.00144191 | -0.00436974 |
| exponential_firstorder_10Percentile | -0.00161031 | 0.00141175 | 0.9065717 | 3 | 0.006479167 | -0.00969978 |
| wavelet-HL_gldm_DependenceVariance | -0.00161031 | 0.0017749 | 0.87165556 | 3 | 0.008560079 | -0.01178069 |
| wavelet-HL_glszm_SmallAreaLowGrayLevelEmphasis | -0.00161031 | 0.00067085 | 0.97336463 | 3 | 0.002233738 | -0.00545435 |
| wavelet-LL_firstorder_RobustMeanAbsoluteDeviation | -0.00161031 | 0.00067085 | 0.97336463 | 3 | 0.002233738 | -0.00545435 |
| logarithm_glrlm_ShortRunEmphasis | -0.00161031 | 0.00126779 | 0.92059551 | 3 | 0.005654255 | -0.00887487 |
| wavelet-LH_glcm_ClusterShade | -0.00161031 | 0.00101423 | 0.94464865 | 3 | 0.004201343 | -0.00742196 |
| original_glrlm_LongRunLowGrayLevelEmphasis | -0.00161031 | 0.00050712 | 0.984248 | 3 | 0.001295518 | -0.00451613 |
| wavelet-LL_glszm_GrayLevelNonUniformityNormalized | -0.00161031 | 0.00110523 | 0.93617837 | 3 | 0.004722791 | -0.0079434 |
| square_glrlm_GrayLevelNonUniformityNormalized | -0.00161031 | 0.00025356 | 0.9959183 | 3 | -0.00015739 | -0.00306322 |
| exponential_firstorder_Skewness | -0.00161031 | 0.00025356 | 0.9959183 | 3 | -0.00015739 | -0.00306322 |
| gradient_glszm_SizeZoneNonUniformity | -0.00161031 | 0.00050712 | 0.984248 | 3 | 0.001295518 | -0.00451613 |
| wavelet-HH_firstorder_Skewness | -0.00161031 | 0.00067085 | 0.97336463 | 3 | 0.002233738 | -0.00545435 |
| wavelet-HL_ngtdm_Contrast | -0.00161031 | 0.00067085 | 0.97336463 | 3 | 0.002233738 | -0.00545435 |
| wavelet-HH_firstorder_Range | -0.00161031 | 0.00025356 | 0.9959183 | 3 | -0.00015739 | -0.00306322 |
| logarithm_glrlm_RunPercentage | -0.0017567 | 0.00076067 | 0.97140452 | 3 | 0.002602039 | -0.00611543 |
| squareroot_glszm_SmallAreaLowGrayLevelEmphasis | -0.0017567 | 0.00152135 | 0.90824829 | 3 | 0.006960776 | -0.01047417 |
| wavelet-LH_firstorder_10Percentile | -0.0017567 | 0.00191432 | 0.87354368 | 3 | 0.009212549 | -0.01272594 |
| wavelet-HL_gldm_DependenceEntropy | -0.0017567 | 6.41E-17 | 1 | 3 | -0.0017567 | -0.0017567 |
| exponential_firstorder_Mean | -0.0017567 | 0.00219587 | 0.84992711 | 3 | 0.010825891 | -0.01433929 |
| wavelet-HL_glcm_Idmn | -0.0017567 | 0.00116195 | 0.93994135 | 3 | 0.004901383 | -0.00841478 |
| gradient_glrlm_ShortRunLowGrayLevelEmphasis | -0.0017567 | 0.00244522 | 0.83028913 | 3 | 0.01225468 | -0.01576808 |
| wavelet-LH_ngtdm_Busyness | -0.0017567 | 0.00043917 | 0.98989795 | 3 | 0.00075982 | -0.00427322 |
| wavelet-LH_glszm_SmallAreaHighGrayLevelEmphasis | -0.0017567 | 0.00043917 | 0.98989795 | 3 | 0.00075982 | -0.00427322 |
| logarithm_gldm_LargeDependenceLowGrayLevelEmphasis | -0.0017567 | 0.00116195 | 0.93994135 | 3 | 0.004901383 | -0.00841478 |
| square_glcm_Contrast | -0.0017567 | 0.00043917 | 0.98989795 | 3 | 0.00075982 | -0.00427322 |
| gradient_glrlm_RunLengthNonUniformity | -0.0017567 | 0.00043917 | 0.98989795 | 3 | 0.00075982 | -0.00427322 |
| original_glrlm_RunEntropy | -0.0017567 | 0.00043917 | 0.98989795 | 3 | 0.00075982 | -0.00427322 |
| wavelet-HH_firstorder_RobustMeanAbsoluteDeviation | -0.0017567 | 0.00043917 | 0.98989795 | 3 | 0.00075982 | -0.00427322 |
| wavelet-LL_firstorder_Mean | -0.0017567 | 0.00152135 | 0.90824829 | 3 | 0.006960776 | -0.01047417 |
| wavelet-HL_glcm_Imc1 | -0.00190309 | 0.00264722 | 0.83041357 | 3 | 0.01326576 | -0.01707194 |
| wavelet-HH_ngtdm_Busyness | -0.00190309 | 0.00050712 | 0.98856992 | 3 | 0.001002736 | -0.00480891 |
| wavelet-LL_gldm_DependenceEntropy | -0.00190309 | 0.00067085 | 0.98049383 | 3 | 0.001940955 | -0.00574713 |
| wavelet-LH_glrlm_LongRunLowGrayLevelEmphasis | -0.00190309 | 0.00025356 | 0.99706742 | 3 | -0.00045018 | -0.003356 |
| wavelet-LH_glrlm_GrayLevelVariance | -0.00190309 | 0.00067085 | 0.98049383 | 3 | 0.001940955 | -0.00574713 |
| wavelet-LH_firstorder_MeanAbsoluteDeviation | -0.00190309 | 0.00110523 | 0.95178123 | 3 | 0.004430009 | -0.00823619 |
| logarithm_gldm_LowGrayLevelEmphasis | -0.00190309 | 0.00141175 | 0.9276686 | 3 | 0.006186384 | -0.00999256 |
| square_glcm_Imc2 | -0.00190309 | 0.00067085 | 0.98049383 | 3 | 0.001940955 | -0.00574713 |
| wavelet-LH_gldm_LargeDependenceLowGrayLevelEmphasis | -0.00190309 | 0.00025356 | 0.99706742 | 3 | -0.00045018 | -0.003356 |
| original_gldm_LargeDependenceLowGrayLevelEmphasis | -0.00190309 | 0.00166269 | 0.90704579 | 3 | 0.007624294 | -0.01143047 |
| wavelet-HL_gldm_SmallDependenceLowGrayLevelEmphasis | -0.00190309 | 0.0013417 | 0.93333333 | 3 | 0.005785 | -0.00959118 |
| wavelet-LH_firstorder_Uniformity | -0.00190309 | 0.00050712 | 0.98856992 | 3 | 0.001002736 | -0.00480891 |
| original_glcm_JointEntropy | -0.00204948 | 0.00067085 | 0.98304589 | 3 | 0.001794564 | -0.00589353 |
| original_gldm_HighGrayLevelEmphasis | -0.00204948 | 0.0028235 | 0.8322053 | 3 | 0.014129465 | -0.01822843 |
| squareroot_firstorder_Kurtosis | -0.00204948 | 0.00067085 | 0.98304589 | 3 | 0.001794564 | -0.00589353 |
| squareroot_gldm_GrayLevelVariance | -0.00204948 | 0.00207546 | 0.88533732 | 3 | 0.009843119 | -0.01394208 |
| wavelet-LH_glcm_MaximumProbability | -0.00204948 | 0.00050712 | 0.99009803 | 3 | 0.000856344 | -0.00495531 |
| squareroot_glcm_ClusterShade | -0.00204948 | 0.00025356 | 0.99746834 | 3 | -0.00059657 | -0.00350239 |
| squareroot_glszm_SizeZoneNonUniformityNormalized | -0.00204948 | 0.00110523 | 0.95760432 | 3 | 0.004283617 | -0.00838258 |
| wavelet-LH_glrlm_LowGrayLevelRunEmphasis | -0.00204948 | 0.00067085 | 0.98304589 | 3 | 0.001794564 | -0.00589353 |
| wavelet-HL_glcm_ClusterProminence | -0.00204948 | 0.00025356 | 0.99746834 | 3 | -0.00059657 | -0.00350239 |
| wavelet-LH_glszm_LargeAreaEmphasis | -0.00204948 | 0.00050712 | 0.99009803 | 3 | 0.000856344 | -0.00495531 |
| wavelet-HH_glcm_SumEntropy | -0.00219587 | 0.00043917 | 0.99346377 | 3 | 0.000320646 | -0.00471239 |
| gradient_glszm_LowGrayLevelZoneEmphasis | -0.00219587 | 0.00228202 | 0.88124643 | 3 | 0.010880338 | -0.01527208 |
| logarithm_glcm_ClusterShade | -0.00219587 | 0.00043917 | 0.99346377 | 3 | 0.000320646 | -0.00471239 |
| wavelet-LH_glrlm_ShortRunHighGrayLevelEmphasis | -0.00219587 | 0.00076067 | 0.98112522 | 3 | 0.002162865 | -0.00655461 |
| square_firstorder_Skewness | -0.00219587 | 0.00076067 | 0.98112522 | 3 | 0.002162865 | -0.00655461 |
| squareroot_gldm_DependenceEntropy | -0.00219587 | 0.00043917 | 0.99346377 | 3 | 0.000320646 | -0.00471239 |
| wavelet-LH_glcm_Imc2 | -0.00234226 | 0.00067085 | 0.9868645 | 3 | 0.001501781 | -0.00618631 |
| wavelet-LH_gldm_LowGrayLevelEmphasis | -0.00234226 | 0.0026834 | 0.86514837 | 3 | 0.013033914 | -0.01771844 |
| wavelet-HH_gldm_LowGrayLevelEmphasis | -0.00234226 | 0.00091421 | 0.97639307 | 3 | 0.002896286 | -0.00758081 |
| wavelet-HL_ngtdm_Strength | -0.00234226 | 0.00067085 | 0.9868645 | 3 | 0.001501781 | -0.00618631 |
| exponential_glrlm_RunLengthNonUniformity | -0.00234226 | 0.00241878 | 0.88225479 | 3 | 0.011517636 | -0.01620216 |
| exponential_firstorder_Variance | -0.00248866 | 0.00050712 | 0.99322003 | 3 | 0.00041717 | -0.00539448 |
| wavelet-HH_glszm_GrayLevelVariance | -0.00263505 | 0.00201255 | 0.92426407 | 3 | 0.008897087 | -0.01416718 |
| wavelet-HL_glcm_MCC | -0.00263505 | 0.00116195 | 0.97043754 | 3 | 0.004023034 | -0.00929313 |
| lymphatic metastasis | -0.00263505 | 0.00152135 | 0.95226702 | 3 | 0.006082427 | -0.01135252 |
| wavelet-HH_glrlm_HighGrayLevelRunEmphasis | -0.00263505 | 0.00043917 | 0.99543369 | 3 | -0.00011853 | -0.00515156 |
| logarithm_glrlm_GrayLevelVariance | -0.00263505 | 0.00087835 | 0.98245064 | 3 | 0.002397989 | -0.00766808 |
| logarithm_firstorder_Maximum | -0.00263505 | 0.00116195 | 0.97043754 | 3 | 0.004023034 | -0.00929313 |
| wavelet-LL_firstorder_Maximum | -0.00278144 | 0.0013417 | 0.965217 | 3 | 0.004906651 | -0.01046953 |
| gradient_glcm_Idmn | -0.00278144 | 0.00110523 | 0.97559487 | 3 | 0.00355166 | -0.00911454 |
| wavelet-HH_glszm_GrayLevelNonUniformity | -0.00278144 | 0.00225367 | 0.91700372 | 3 | 0.010132328 | -0.0156952 |
| squareroot_glcm_InverseVariance | -0.00278144 | 0.00166269 | 0.94933458 | 3 | 0.006745945 | -0.01230882 |
| exponential_glrlm_ShortRunEmphasis | -0.00278144 | 0.00101423 | 0.97921161 | 3 | 0.003030211 | -0.00859309 |
| squareroot_ngtdm_Complexity | -0.00292783 | 0.00101423 | 0.98112522 | 3 | 0.00288382 | -0.00873948 |
| wavelet-LH_glcm_InverseVariance | -0.00292783 | 0.00249725 | 0.91030497 | 3 | 0.011381697 | -0.01723736 |
| logarithm_glszm_SmallAreaLowGrayLevelEmphasis | -0.00292783 | 0.00067085 | 0.99147319 | 3 | 0.000916215 | -0.00677187 |
| wavelet-LH_firstorder_Skewness | -0.00307422 | 0.00076067 | 0.99009803 | 3 | 0.001284516 | -0.00743296 |
| squareroot_glcm_Idn | -0.00307422 | 0.00158347 | 0.96089885 | 3 | 0.005999213 | -0.01214765 |
| wavelet-LH_glszm_LowGrayLevelZoneEmphasis | -0.00307422 | 0.00116195 | 0.97776654 | 3 | 0.00358386 | -0.0097323 |
| wavelet-LH_firstorder_Mean | -0.00307422 | 0.00191432 | 0.94570018 | 3 | 0.007895026 | -0.01404347 |
| wavelet-LH_gldm_LargeDependenceHighGrayLevelEmphasis | -0.00307422 | 0.00331569 | 0.87523939 | 3 | 0.015925072 | -0.02207351 |
| wavelet-HL_glrlm_RunEntropy | -0.00322061 | 0.00050712 | 0.9959183 | 3 | -0.00031479 | -0.00612644 |
| wavelet-LH_ngtdm_Complexity | -0.00322061 | 0.00050712 | 0.9959183 | 3 | -0.00031479 | -0.00612644 |
| wavelet-HH_firstorder_MeanAbsoluteDeviation | -0.00322061 | 0.00025356 | 0.99897013 | 3 | -0.0017677 | -0.00467352 |
| wavelet-HL_firstorder_Range | -0.00322061 | 0.00025356 | 0.99897013 | 3 | -0.0017677 | -0.00467352 |
| wavelet-HH_firstorder_Maximum | -0.0035134 | 0.00076067 | 0.99236596 | 3 | 0.000845342 | -0.00787213 |
| wavelet-HH_firstorder_Mean | -0.0035134 | 0.00232389 | 0.93994135 | 3 | 0.009802765 | -0.01682956 |
| wavelet-LH_gldm_DependenceVariance | -0.0035134 | 0.00307422 | 0.9068381 | 3 | 0.014102229 | -0.02112902 |
| logarithm_firstorder_Range | -0.00365979 | 0.0028235 | 0.9230609 | 3 | 0.012519159 | -0.01983873 |
| wavelet-HL_firstorder_Kurtosis | -0.00365979 | 0.00141175 | 0.97690475 | 3 | 0.004429686 | -0.01174926 |
| gradient_glcm_DifferenceVariance | -0.00380618 | 0.00198035 | 0.96019501 | 3 | 0.007541429 | -0.01515379 |
| wavelet-HH_glcm_MaximumProbability | -0.00395257 | 0.00043917 | 0.997955 | 3 | -0.00143605 | -0.00646909 |
| logarithm_firstorder_Minimum | -0.00395257 | 0.00087835 | 0.99196735 | 3 | 0.001080466 | -0.00898561 |
| squareroot_glcm_Idmn | -0.00409896 | 0.00292417 | 0.93204938 | 3 | 0.01265684 | -0.02085476 |
| logarithm_gldm_SmallDependenceHighGrayLevelEmphasis | -0.00409896 | 0.00225367 | 0.95614465 | 3 | 0.008814805 | -0.01701273 |
| wavelet-HH_glcm_ClusterShade | -0.00439174 | 0.00232389 | 0.95899255 | 3 | 0.008924417 | -0.0177079 |
| logarithm_glszm_LargeAreaLowGrayLevelEmphasis | -0.00453814 | 0.00101423 | 0.99187763 | 3 | 0.001273514 | -0.01034978 |
| square_glcm_Autocorrelation | -0.0051237 | 0.00067085 | 0.99716712 | 3 | -0.00127966 | -0.00896775 |
| wavelet-LH_glszm_SmallAreaEmphasis | -0.00541648 | 0.00241878 | 0.96974896 | 3 | 0.008443415 | -0.01927638 |
| gradient_ngtdm_Complexity | -0.00614844 | 0.00445713 | 0.93027728 | 3 | 0.019391425 | -0.03168831 |
| wavelet-HH_firstorder_10Percentile | -0.00614844 | 0.00348584 | 0.95374261 | 3 | 0.013825799 | -0.02612268 |
| logarithm_ngtdm_Complexity | -0.00629483 | 0.00067085 | 0.99811777 | 3 | -0.00245079 | -0.01013888 |
| logarithm_glcm_InverseVariance | -0.0086371 | 0.0046547 | 0.9576532 | 3 | 0.018034827 | -0.03530902 |

The feature name. ‘importance’: The estimated feature importance score. ‘stddev’: The standard deviation of the feature importance score. If NaN, then not enough num_shuffle_sets were used to calculate a variance. ‘p_value’: P-value for a statistical t-test of the null hypothesis: importance = 0, vs the (one-sided) alternative: importance > 0.

Features with low p-value appear confidently useful to the predictor, while the other features may be useless to the predictor (or even harmful to include in its training data). A p-value of 0.01 indicates that there is a 1% chance that the feature is useless or harmful, and a 99% chance that the feature is useful. A p-value of 0.99 indicates that there is a 99% chance that the feature is useless or harmful, and a 1% chance that the feature is useful.

‘n’: The number of shuffles performed to estimate importance score (corresponds to sample-size used to determine confidence interval for true score). ‘p99_high’: Upper end of 99% confidence interval for true feature importance score. ‘p99_low’: Lower end of 99% confidence interval for true feature importance score.
