# Supplementary material for: Using combined CT-clinical radiomics models to identify epidermal growth factor receptor mutation subtypes in lung adenocarcinoma
Source: Front Oncol. 2022 Aug 18;12:846589. doi: 10.3389/fonc.2022.846589 (PMC9434115; doi:10.3389/fonc.2022.846589)
Supplement: Supplementary file 2 [file Table_2.docx]

**Supplementary Table 2** The radiomics, clinical, and CT morphology features and their importance scores for model 3

| **Features** | **importance** | **stddev** | **p_value** | **n** | **p99_high** | **p99_low** |
| --- | --- | --- | --- | --- | --- | --- |
| smoking history | 0.083077971 | 0.0305431 | 0.02111057 | 3 | 0.258093297 | -0.09193736 |
| sex | 0.075700829 | 0.01932775 | 0.01052275 | 3 | 0.186450976 | -0.03504932 |
| pleural retraction | 0.055725797 | 0.02383466 | 0.02795706 | 3 | 0.192301012 | -0.08084942 |
| vscular convergence sign | 0.016229713 | 0.0058612 | 0.02041497 | 3 | 0.049815021 | -0.0173556 |
| wavelet-HH_firstorder_Median | 0.011576439 | 0.00566678 | 0.03571087 | 3 | 0.044047685 | -0.02089481 |
| wavelet-HL_firstorder_Mean | 0.00783112 | 0.00238338 | 0.01475785 | 3 | 0.021488177 | -0.00582594 |
| wavelet-HH_glszm_SizeZoneNonUniformity | 0.007377142 | 0.00226705 | 0.0150334 | 3 | 0.020367595 | -0.00561331 |
| gradient_gldm_LowGrayLevelEmphasis | 0.006128703 | 0.00238338 | 0.02344696 | 3 | 0.01978576 | -0.00752835 |
| squareroot_glcm_Correlation | 0.005674725 | 0.00128905 | 0.0083843 | 3 | 0.013061122 | -0.00171167 |
| wavelet-LH_gldm_DependenceNonUniformityNormalized | 0.00556123 | 0.00327171 | 0.04930022 | 3 | 0.024308513 | -0.01318605 |
| gradient_glcm_ClusterProminence | 0.005220747 | 0.00193607 | 0.02145603 | 3 | 0.016314649 | -0.00587316 |
| lymphatic metastasis | 0.004993758 | 0.00870955 | 0.21265756 | 3 | 0.054900429 | -0.04491291 |
| wavelet-LH_firstorder_10Percentile | 0.004880263 | 0.00019658 | 0.0002702 | 3 | 0.006006678 | 0.003753848 |
| logarithm_glcm_InverseVariance | 0.004766769 | 0.00863379 | 0.2199253 | 3 | 0.05423935 | -0.04470581 |
| location | 0.004653274 | 0.00264469 | 0.04645574 | 3 | 0.019807639 | -0.01050109 |
| wavelet-LH_glcm_ClusterTendency | 0.004653274 | 0.00039316 | 0.00118554 | 3 | 0.006906104 | 0.002400444 |
| wavelet-LH_glszm_GrayLevelNonUniformityNormalized | 0.00453978 | 0.00160906 | 0.01970789 | 3 | 0.013759885 | -0.00468033 |
| wavelet-LH_firstorder_RobustMeanAbsoluteDeviation | 0.004312791 | 0.00137605 | 0.01614926 | 3 | 0.012197696 | -0.00357212 |
| spiculation | 0.004199296 | 0.00305172 | 0.06999994 | 3 | 0.021685961 | -0.01328737 |
| exponential_gldm_SmallDependenceHighGrayLevelEmphasis | 0.004199296 | 0.00316363 | 0.07412144 | 3 | 0.022327231 | -0.01392864 |
| exponential_glrlm_ShortRunHighGrayLevelEmphasis | 0.004199296 | 0.00187524 | 0.03025104 | 3 | 0.014944611 | -0.00654602 |
| wavelet-LH_firstorder_RootMeanSquared | 0.003858813 | 0.00157263 | 0.02557626 | 3 | 0.012870133 | -0.00515251 |
| logarithm_glcm_MCC | 0.003858813 | 0.00255552 | 0.06018137 | 3 | 0.018502209 | -0.01078458 |
| wavelet-LH_glcm_SumSquares | 0.003745318 | 0.00345553 | 0.10063759 | 3 | 0.023545888 | -0.01605525 |
| logarithm_glcm_Correlation | 0.003745318 | 0.00383705 | 0.11648643 | 3 | 0.025732063 | -0.01824143 |
| wavelet-LH_firstorder_Skewness | 0.003518329 | 0.00289578 | 0.08500335 | 3 | 0.02011146 | -0.0130748 |
| wavelet-HL_gldm_LargeDependenceEmphasis | 0.003404835 | 0.0017692 | 0.03971269 | 3 | 0.013542571 | -0.0067329 |
| exponential_glszm_SmallAreaLowGrayLevelEmphasis | 0.003404835 | 0.00212632 | 0.0545646 | 3 | 0.015588877 | -0.00877921 |
| original_glcm_Idn | 0.003404835 | 0.00034048 | 0.00165838 | 3 | 0.005355843 | 0.001453827 |
| squareroot_glszm_LowGrayLevelZoneEmphasis | 0.003404835 | 0.00360334 | 0.12167505 | 3 | 0.024052364 | -0.01724269 |
| original_firstorder_MeanAbsoluteDeviation | 0.00329134 | 0.0005201 | 0.00411046 | 3 | 0.006271555 | 0.000311126 |
| wavelet-HL_ngtdm_Complexity | 0.00329134 | 0.00085687 | 0.01092714 | 3 | 0.00820127 | -0.00161859 |
| original_glszm_LargeAreaLowGrayLevelEmphasis | 0.00329134 | 0.00561884 | 0.20853903 | 3 | 0.035487901 | -0.02890522 |
| wavelet-LH_glcm_MCC | 0.003177846 | 0.00411877 | 0.15659033 | 3 | 0.026778863 | -0.02042317 |
| original_glcm_Imc2 | 0.003177846 | 0.00171373 | 0.04239569 | 3 | 0.012997705 | -0.00664201 |
| wavelet-LH_gldm_SmallDependenceEmphasis | 0.003064351 | 0.00117947 | 0.02300095 | 3 | 0.009822842 | -0.00369414 |
| wavelet-LH_glrlm_RunPercentage | 0.003064351 | 0.00034048 | 0.002045 | 3 | 0.00501536 | 0.001113343 |
| wavelet-LH_gldm_GrayLevelVariance | 0.002950857 | 0.00157263 | 0.04152535 | 3 | 0.011962177 | -0.00606046 |
| clinical stage | 0.002950857 | 0.00289578 | 0.10980485 | 3 | 0.019543987 | -0.01364227 |
| wavelet-LH_glszm_ZonePercentage | 0.002950857 | 0.00039316 | 0.00293258 | 3 | 0.005203687 | 0.000698027 |
| gradient_ngtdm_Strength | 0.002950857 | 0.00308947 | 0.11994153 | 3 | 0.020653859 | -0.01475215 |
| wavelet-LL_glrlm_RunVariance | 0.002837362 | 0.0027521 | 0.10803297 | 3 | 0.018607173 | -0.01293245 |
| gradient_gldm_GrayLevelVariance | 0.002837362 | 0.00373499 | 0.15941445 | 3 | 0.024239249 | -0.01856452 |
| squareroot_glcm_Imc1 | 0.002837362 | 0.00199505 | 0.0663798 | 3 | 0.014269227 | -0.0085945 |
| wavelet-LL_glcm_ClusterTendency | 0.002837362 | 0.0010945 | 0.02309525 | 3 | 0.009108976 | -0.00343425 |
| gradient_glrlm_HighGrayLevelRunEmphasis | 0.002837362 | 0.0027521 | 0.10803297 | 3 | 0.018607173 | -0.01293245 |
| wavelet-HH_firstorder_RobustMeanAbsoluteDeviation | 0.002837362 | 0.00039316 | 0.0031696 | 3 | 0.005090193 | 0.000584532 |
| squareroot_glcm_Idn | 0.002837362 | 0.002189 | 0.0769391 | 3 | 0.01538059 | -0.00970587 |
| squareroot_glrlm_LongRunLowGrayLevelEmphasis | 0.002837362 | 0.0045341 | 0.19584484 | 3 | 0.028818267 | -0.02314354 |
| original_glszm_ZonePercentage | 0.002723868 | 0.00034048 | 0.002584 | 3 | 0.004674876 | 0.00077286 |
| square_glcm_Idmn | 0.002723868 | 0.00180167 | 0.06005866 | 3 | 0.013047633 | -0.0075999 |
| squareroot_glrlm_LongRunHighGrayLevelEmphasis | 0.002610373 | 0.00187524 | 0.06871631 | 3 | 0.013355688 | -0.00813494 |
| wavelet-HL_glcm_ClusterProminence | 0.002610373 | 0.00019658 | 0.00094251 | 3 | 0.003736788 | 0.001483958 |
| wavelet-HH_glszm_GrayLevelNonUniformityNormalized | 0.002610373 | 0.00208039 | 0.0809167 | 3 | 0.01453123 | -0.00931048 |
| wavelet-HL_glrlm_RunVariance | 0.002610373 | 0.00039316 | 0.00373837 | 3 | 0.004863204 | 0.000357543 |
| wavelet-HH_firstorder_InterquartileRange | 0.002610373 | 0.0005201 | 0.00648776 | 3 | 0.005590588 | -0.00036984 |
| squareroot_firstorder_Minimum | 0.002610373 | 0.00019658 | 0.00094251 | 3 | 0.003736788 | 0.001483958 |
| original_glrlm_RunEntropy | 0.002610373 | 0.00119574 | 0.0316838 | 3 | 0.009462089 | -0.00424134 |
| wavelet-LL_glcm_JointEnergy | 0.002496879 | 0.0027521 | 0.12834444 | 3 | 0.01826669 | -0.01327293 |
| maximum diameter | 0.002496879 | 0.00039316 | 0.0040817 | 3 | 0.004749709 | 0.000244049 |
| square_glcm_Correlation | 0.002496879 | 0.0005201 | 0.00707821 | 3 | 0.005477093 | -0.00048334 |
| squareroot_glszm_GrayLevelVariance | 0.002496879 | 0.00187524 | 0.07375866 | 3 | 0.013242194 | -0.00824844 |
| wavelet-HL_ngtdm_Strength | 0.002496879 | 0.00406209 | 0.1992784 | 3 | 0.025773096 | -0.02077934 |
| exponential_glrlm_ShortRunLowGrayLevelEmphasis | 0.002496879 | 0.00208039 | 0.08659469 | 3 | 0.014417736 | -0.00942398 |
| wavelet-HH_ngtdm_Coarseness | 0.002383384 | 0 | 0.5 | 3 | 0.002383384 | 0.002383384 |
| exponential_glszm_ZonePercentage | 0.002383384 | 0 | 0.5 | 3 | 0.002383384 | 0.002383384 |
| wavelet-HL_ngtdm_Contrast | 0.002383384 | 0.00117947 | 0.03641368 | 3 | 0.009141875 | -0.00437511 |
| wavelet-HH_glrlm_ShortRunEmphasis | 0.002383384 | 0.00058974 | 0.00990197 | 3 | 0.00576263 | -0.00099586 |
| wavelet-LH_firstorder_MeanAbsoluteDeviation | 0.002383384 | 0.00379146 | 0.19497873 | 3 | 0.024108892 | -0.01934212 |
| exponential_gldm_DependenceEntropy | 0.002383384 | 0.00156029 | 0.05904145 | 3 | 0.011324027 | -0.00655726 |
| pleural effusion | 0.002383384 | 0.0020429 | 0.09035599 | 3 | 0.014089433 | -0.00932266 |
| exponential_glrlm_ShortRunEmphasis | 0.002383384 | 0.00238338 | 0.11270167 | 3 | 0.016040441 | -0.01127367 |
| original_glszm_SmallAreaLowGrayLevelEmphasis | 0.002383384 | 0.00294867 | 0.14823677 | 3 | 0.01927961 | -0.01451284 |
| squareroot_glcm_ClusterProminence | 0.002383384 | 0 | 0.5 | 3 | 0.002383384 | 0.002383384 |
| wavelet-LH_glrlm_RunLengthNonUniformityNormalized | 0.00226989 | 0.00196578 | 0.09175171 | 3 | 0.013534041 | -0.00899426 |
| square_gldm_LargeDependenceEmphasis | 0.00226989 | 0.00070877 | 0.01549842 | 3 | 0.006331237 | -0.00179146 |
| wavelet-LH_firstorder_Variance | 0.00226989 | 0.00239148 | 0.12095098 | 3 | 0.015973321 | -0.01143354 |
| wavelet-HH_firstorder_Mean | 0.00226989 | 0.00160906 | 0.06725768 | 3 | 0.011489995 | -0.00695022 |
| squareroot_glrlm_RunEntropy | 0.002156395 | 0.00137605 | 0.05657797 | 3 | 0.010041301 | -0.00572851 |
| squareroot_glszm_SizeZoneNonUniformity | 0.002156395 | 0.00208039 | 0.10722334 | 3 | 0.014077252 | -0.00976446 |
| wavelet-LH_glcm_Id | 0.002156395 | 0.00137605 | 0.05657797 | 3 | 0.010041301 | -0.00572851 |
| original_gldm_GrayLevelVariance | 0.002156395 | 0.00128905 | 0.05066542 | 3 | 0.009542793 | -0.00523 |
| wavelet-LH_glcm_Idm | 0.002156395 | 0.00137605 | 0.05657797 | 3 | 0.010041301 | -0.00572851 |
| wavelet-LH_glrlm_GrayLevelVariance | 0.002156395 | 0.00019658 | 0.00137931 | 3 | 0.00328281 | 0.00102998 |
| wavelet-LL_gldm_SmallDependenceLowGrayLevelEmphasis | 0.002042901 | 0.00238338 | 0.13796691 | 3 | 0.015699958 | -0.01161416 |
| wavelet-HH_firstorder_10Percentile | 0.002042901 | 0.00122763 | 0.0511208 | 3 | 0.009077361 | -0.00499156 |
| gradient_glcm_Imc2 | 0.002042901 | 0.00034048 | 0.00456631 | 3 | 0.003993909 | 9.19E-05 |
| wavelet-LH_gldm_LargeDependenceEmphasis | 0.002042901 | 0.00148413 | 0.06996337 | 3 | 0.010547148 | -0.00646135 |
| wavelet-LL_glrlm_RunEntropy | 0.002042901 | 0.00102145 | 0.03708995 | 3 | 0.007895925 | -0.00381012 |
| squareroot_gldm_LowGrayLevelEmphasis | 0.001929406 | 0.00104019 | 0.04237538 | 3 | 0.007889835 | -0.00403102 |
| gradient_glcm_MCC | 0.001929406 | 0.00208039 | 0.12471583 | 3 | 0.013850263 | -0.00999145 |
| necrosis | 0.001929406 | 0.0005201 | 0.01168776 | 3 | 0.004909621 | -0.00105081 |
| original_glcm_SumSquares | 0.001815912 | 0.00141755 | 0.07836298 | 3 | 0.009938607 | -0.00630678 |
| square_ngtdm_Coarseness | 0.001815912 | 0.00019658 | 0.00194176 | 3 | 0.002942327 | 0.000689497 |
| square_firstorder_MeanAbsoluteDeviation | 0.001815912 | 0.00128905 | 0.06740954 | 3 | 0.00920231 | -0.00557049 |
| wavelet-LL_glrlm_RunLengthNonUniformityNormalized | 0.001815912 | 0.00187524 | 0.11774521 | 3 | 0.012561227 | -0.0089294 |
| wavelet-LH_ngtdm_Coarseness | 0.001815912 | 0.00128905 | 0.06740954 | 3 | 0.00920231 | -0.00557049 |
| wavelet-HL_glcm_ClusterShade | 0.001815912 | 0.00085687 | 0.03343053 | 3 | 0.006725841 | -0.00309402 |
| wavelet-LL_glcm_Idm | 0.001815912 | 0.00070877 | 0.02360693 | 3 | 0.005877259 | -0.00224544 |
| gradient_gldm_SmallDependenceLowGrayLevelEmphasis | 0.001815912 | 0.00160906 | 0.09490425 | 3 | 0.011036017 | -0.00740419 |
| original_glcm_Correlation | 0.001815912 | 0.00193607 | 0.12287638 | 3 | 0.012909814 | -0.00927799 |
| wavelet-LL_glcm_SumEntropy | 0.001815912 | 0.00019658 | 0.00194176 | 3 | 0.002942327 | 0.000689497 |
| logarithm_glszm_SmallAreaLowGrayLevelEmphasis | 0.001702417 | 0.00102145 | 0.05098675 | 3 | 0.007555442 | -0.00415061 |
| original_glcm_DifferenceAverage | 0.001702417 | 0.00034048 | 0.00653623 | 3 | 0.003653426 | -0.00024859 |
| gradient_firstorder_Variance | 0.001702417 | 0.00324801 | 0.2298941 | 3 | 0.020313849 | -0.01690901 |
| wavelet-LH_firstorder_90Percentile | 0.001702417 | 0.00156029 | 0.09967962 | 3 | 0.01064306 | -0.00723823 |
| wavelet-LH_glcm_DifferenceAverage | 0.001702417 | 0.00148413 | 0.09265592 | 3 | 0.010206665 | -0.00680183 |
| wavelet-LL_firstorder_Entropy | 0.001702417 | 0.00148413 | 0.09265592 | 3 | 0.010206665 | -0.00680183 |
| square_glcm_Idn | 0.001702417 | 0.0017692 | 0.11875357 | 3 | 0.011840153 | -0.00843532 |
| wavelet-HL_glszm_LargeAreaLowGrayLevelEmphasis | 0.001588923 | 0.0005201 | 0.01695411 | 3 | 0.004569137 | -0.00139129 |
| original_glszm_SizeZoneNonUniformity | 0.001588923 | 0.0027521 | 0.21132487 | 3 | 0.017358734 | -0.01418089 |
| squareroot_glcm_Idmn | 0.001588923 | 0.00160906 | 0.11466268 | 3 | 0.010809028 | -0.00763118 |
| wavelet-LL_glcm_Imc2 | 0.001588923 | 0.00208039 | 0.15843497 | 3 | 0.01350978 | -0.01033193 |
| wavelet-LH_firstorder_InterquartileRange | 0.001588923 | 0.00160906 | 0.11466268 | 3 | 0.010809028 | -0.00763118 |
| gradient_glrlm_ShortRunLowGrayLevelEmphasis | 0.001588923 | 0.00314525 | 0.23692389 | 3 | 0.019611564 | -0.01643372 |
| squareroot_glcm_SumSquares | 0.001588923 | 0.00246312 | 0.19003479 | 3 | 0.015702863 | -0.01252502 |
| wavelet-LL_glcm_MaximumProbability | 0.001588923 | 0.00432472 | 0.29482637 | 3 | 0.026370055 | -0.02319221 |
| exponential_glszm_SmallAreaHighGrayLevelEmphasis | 0.001588923 | 0.00085687 | 0.04239569 | 3 | 0.006498852 | -0.00332101 |
| logarithm_ngtdm_Busyness | 0.001588923 | 0.0005201 | 0.01695411 | 3 | 0.004569137 | -0.00139129 |
| square_glrlm_RunLengthNonUniformity | 0.001588923 | 0.00137605 | 0.09175171 | 3 | 0.009473828 | -0.00629598 |
| wavelet-LH_ngtdm_Strength | 0.001588923 | 0.00141755 | 0.09585481 | 3 | 0.009711618 | -0.00653377 |
| wavelet-LL_glszm_SizeZoneNonUniformity | 0.001475428 | 0.00289578 | 0.23529954 | 3 | 0.018068559 | -0.0151177 |
| wavelet-LL_glcm_SumSquares | 0.001475428 | 0.00137605 | 0.10220646 | 3 | 0.009360334 | -0.00640948 |
| wavelet-LL_glrlm_LongRunEmphasis | 0.001475428 | 0.00255552 | 0.21132487 | 3 | 0.016118824 | -0.01316797 |
| squareroot_glszm_LargeAreaLowGrayLevelEmphasis | 0.001475428 | 0.00098289 | 0.0607707 | 3 | 0.007107504 | -0.00415665 |
| original_glrlm_GrayLevelVariance | 0.001475428 | 0.00137605 | 0.10220646 | 3 | 0.009360334 | -0.00640948 |
| wavelet-HH_glcm_Imc1 | 0.001475428 | 0.00141755 | 0.1066021 | 3 | 0.009598123 | -0.00664727 |
| wavelet-LL_glrlm_GrayLevelVariance | 0.001475428 | 0.00137605 | 0.10220646 | 3 | 0.009360334 | -0.00640948 |
| squareroot_gldm_GrayLevelVariance | 0.001475428 | 0.00137605 | 0.10220646 | 3 | 0.009360334 | -0.00640948 |
| original_glcm_ClusterTendency | 0.001475428 | 0.00137605 | 0.10220646 | 3 | 0.009360334 | -0.00640948 |
| squareroot_glrlm_ShortRunLowGrayLevelEmphasis | 0.001475428 | 0.00273095 | 0.22409037 | 3 | 0.017124087 | -0.01417323 |
| logarithm_ngtdm_Strength | 0.001475428 | 0.00199505 | 0.16434144 | 3 | 0.012907293 | -0.00995644 |
| wavelet-LL_firstorder_MeanAbsoluteDeviation | 0.001475428 | 0.00137605 | 0.10220646 | 3 | 0.009360334 | -0.00640948 |
| squareroot_glrlm_GrayLevelVariance | 0.001475428 | 0.00137605 | 0.10220646 | 3 | 0.009360334 | -0.00640948 |
| squareroot_glcm_SumEntropy | 0.001475428 | 0.00137605 | 0.10220646 | 3 | 0.009360334 | -0.00640948 |
| wavelet-LL_firstorder_Variance | 0.001475428 | 0.00137605 | 0.10220646 | 3 | 0.009360334 | -0.00640948 |
| squareroot_ngtdm_Strength | 0.001475428 | 0.00070877 | 0.03452533 | 3 | 0.005536776 | -0.00258592 |
| original_firstorder_Variance | 0.001475428 | 0.00137605 | 0.10220646 | 3 | 0.009360334 | -0.00640948 |
| wavelet-HL_glcm_DifferenceVariance | 0.001475428 | 0.00098289 | 0.0607707 | 3 | 0.007107504 | -0.00415665 |
| square_glszm_ZonePercentage | 0.001475428 | 0.00153533 | 0.11896325 | 3 | 0.010273011 | -0.00732216 |
| wavelet-LH_gldm_DependenceEntropy | 0.001475428 | 0.00137605 | 0.10220646 | 3 | 0.009360334 | -0.00640948 |
| logarithm_gldm_LargeDependenceHighGrayLevelEmphasis | 0.001475428 | 0.00128905 | 0.09295421 | 3 | 0.008861826 | -0.00591097 |
| squareroot_firstorder_Entropy | 0.001475428 | 0.00196578 | 0.16162352 | 3 | 0.012739579 | -0.00978872 |
| gradient_glcm_SumSquares | 0.001361934 | 0.00412814 | 0.31268284 | 3 | 0.02501665 | -0.02229278 |
| square_firstorder_Uniformity | 0.001361934 | 0.00034048 | 0.01010205 | 3 | 0.003312942 | -0.00058907 |
| gradient_gldm_LargeDependenceHighGrayLevelEmphasis | 0.001361934 | 0.00302628 | 0.2586446 | 3 | 0.018702874 | -0.01597901 |
| gradient_firstorder_RobustMeanAbsoluteDeviation | 0.001361934 | 0.00272387 | 0.23888352 | 3 | 0.016969999 | -0.01424613 |
| gradient_glrlm_LowGrayLevelRunEmphasis | 0.001361934 | 0.00180167 | 0.16031689 | 3 | 0.011685699 | -0.00896183 |
| gradient_glszm_GrayLevelNonUniformity | 0.001361934 | 0.00117947 | 0.09175171 | 3 | 0.008120424 | -0.00539656 |
| squareroot_glszm_ZoneVariance | 0.001248439 | 0.00241559 | 0.23258085 | 3 | 0.01509006 | -0.01259318 |
| square_glcm_DifferenceAverage | 0.001248439 | 0.00160906 | 0.15557664 | 3 | 0.010468544 | -0.00797167 |
| gradient_glcm_ClusterShade | 0.001248439 | 0.0027521 | 0.25716907 | 3 | 0.01701825 | -0.01452137 |
| original_firstorder_Energy | 0.001248439 | 0.00085687 | 0.06382163 | 3 | 0.006158369 | -0.00366149 |
| gradient_firstorder_10Percentile | 0.001248439 | 0.00160906 | 0.15557664 | 3 | 0.010468544 | -0.00797167 |
| square_glrlm_RunPercentage | 0.001248439 | 0.00160906 | 0.15557664 | 3 | 0.010468544 | -0.00797167 |
| square_glcm_Id | 0.001248439 | 0.00160906 | 0.15557664 | 3 | 0.010468544 | -0.00797167 |
| original_glszm_ZoneVariance | 0.001248439 | 0.00241559 | 0.23258085 | 3 | 0.01509006 | -0.01259318 |
| wavelet-LH_glcm_MaximumProbability | 0.001248439 | 0.0005201 | 0.02663537 | 3 | 0.004228654 | -0.00173178 |
| wavelet-LH_glcm_SumEntropy | 0.001248439 | 0.00187524 | 0.18403326 | 3 | 0.011993754 | -0.00949688 |
| square_glcm_Idm | 0.001248439 | 0.00160906 | 0.15557664 | 3 | 0.010468544 | -0.00797167 |
| wavelet-LH_glszm_ZoneVariance | 0.001248439 | 0.00411877 | 0.3259877 | 3 | 0.024849456 | -0.02235258 |
| square_glszm_SmallAreaHighGrayLevelEmphasis | 0.001248439 | 0.00196578 | 0.19301994 | 3 | 0.01251259 | -0.01001571 |
| gradient_gldm_DependenceNonUniformityNormalized | 0.001248439 | 0.00078631 | 0.05535135 | 3 | 0.0057541 | -0.00325722 |
| logarithm_gldm_LowGrayLevelEmphasis | 0.001248439 | 0.00264469 | 0.24974134 | 3 | 0.016402804 | -0.01390593 |
| squareroot_glcm_Imc2 | 0.001248439 | 0.00098289 | 0.07940449 | 3 | 0.006880515 | -0.00438364 |
| wavelet-HL_glcm_SumSquares | 0.001134945 | 0.0005201 | 0.03170709 | 3 | 0.004115159 | -0.00184527 |
| wavelet-HL_firstorder_Entropy | 0.001134945 | 0.00393157 | 0.33333333 | 3 | 0.023663246 | -0.02139336 |
| wavelet-HH_glrlm_RunLengthNonUniformityNormalized | 0.001134945 | 0.00019658 | 0.00492623 | 3 | 0.00226136 | 8.53E-06 |
| logarithm_glszm_LowGrayLevelZoneEmphasis | 0.001134945 | 0.00128905 | 0.13338221 | 3 | 0.008521343 | -0.00625145 |
| squareroot_glcm_MCC | 0.001134945 | 0.0043113 | 0.3465709 | 3 | 0.025839156 | -0.02356927 |
| wavelet-HL_firstorder_InterquartileRange | 0.001134945 | 0.00098289 | 0.09175171 | 3 | 0.00676702 | -0.00449713 |
| wavelet-LH_glcm_JointEntropy | 0.001134945 | 0.00199505 | 0.21416903 | 3 | 0.012566809 | -0.01029692 |
| wavelet-LH_ngtdm_Contrast | 0.001134945 | 0.00255552 | 0.26109076 | 3 | 0.015778341 | -0.01350845 |
| gradient_firstorder_Minimum | 0.001134945 | 0.00137605 | 0.14466547 | 3 | 0.00901985 | -0.00674996 |
| wavelet-LL_glcm_Imc1 | 0.001134945 | 0.00153533 | 0.16442197 | 3 | 0.009932528 | -0.00766264 |
| wavelet-LL_glcm_Contrast | 0.001134945 | 0.00469736 | 0.35812391 | 3 | 0.028051316 | -0.02578143 |
| wavelet-HL_glrlm_LongRunEmphasis | 0.001134945 | 0.0010945 | 0.1071629 | 3 | 0.007406559 | -0.00513667 |
| gradient_ngtdm_Busyness | 0.001134945 | 0.00085687 | 0.07437174 | 3 | 0.006044874 | -0.00377499 |
| wavelet-LL_gldm_GrayLevelVariance | 0.001134945 | 0.00153533 | 0.16442197 | 3 | 0.009932528 | -0.00766264 |
| wavelet-LH_firstorder_Mean | 0.001134945 | 0.00019658 | 0.00492623 | 3 | 0.00226136 | 8.53E-06 |
| wavelet-HL_ngtdm_Coarseness | 0.001134945 | 0.00137605 | 0.14466547 | 3 | 0.00901985 | -0.00674996 |
| wavelet-HL_ngtdm_Busyness | 0.001134945 | 0.00039316 | 0.01887478 | 3 | 0.003387775 | -0.00111789 |
| original_glcm_Imc1 | 0.001134945 | 0.00098289 | 0.09175171 | 3 | 0.00676702 | -0.00449713 |
| wavelet-HL_gldm_DependenceNonUniformityNormalized | 0.001134945 | 0.0010945 | 0.1071629 | 3 | 0.007406559 | -0.00513667 |
| wavelet-LL_ngtdm_Coarseness | 0.001134945 | 0.00039316 | 0.01887478 | 3 | 0.003387775 | -0.00111789 |
| exponential_firstorder_Energy | 0.00102145 | 0.00034048 | 0.01754936 | 3 | 0.002972459 | -0.00092956 |
| squareroot_gldm_LargeDependenceLowGrayLevelEmphasis | 0.00102145 | 0.00122763 | 0.14312679 | 3 | 0.00805591 | -0.00601301 |
| wavelet-LL_glcm_InverseVariance | 0.00102145 | 0.0020429 | 0.23888352 | 3 | 0.012727499 | -0.0106846 |
| square_glrlm_LongRunLowGrayLevelEmphasis | 0.00102145 | 0.00102145 | 0.11270167 | 3 | 0.006874475 | -0.00483157 |
| square_gldm_DependenceVariance | 0.00102145 | 0.00245526 | 0.27300503 | 3 | 0.01509037 | -0.01304747 |
| wavelet-HL_glcm_Imc1 | 0.00102145 | 0.00180167 | 0.21482409 | 3 | 0.011345215 | -0.00930231 |
| gradient_glcm_Correlation | 0.00102145 | 0.00122763 | 0.14312679 | 3 | 0.00805591 | -0.00601301 |
| logarithm_glrlm_GrayLevelNonUniformityNormalized | 0.000907956 | 0.00316363 | 0.33419467 | 3 | 0.019035891 | -0.01721998 |
| wavelet-HH_gldm_GrayLevelVariance | 0.000907956 | 0.00283509 | 0.31742581 | 3 | 0.017153345 | -0.01533743 |
| wavelet-HL_glcm_InverseVariance | 0.000907956 | 0.0010945 | 0.14365168 | 3 | 0.00717957 | -0.00536366 |
| gradient_glcm_InverseVariance | 0.000907956 | 0.00167957 | 0.22397378 | 3 | 0.010532051 | -0.00871614 |
| square_gldm_SmallDependenceHighGrayLevelEmphasis | 0.000907956 | 0.00231762 | 0.28370477 | 3 | 0.014188194 | -0.01237228 |
| gradient_glszm_GrayLevelVariance | 0.000907956 | 0.00255552 | 0.30049813 | 3 | 0.015551352 | -0.01373544 |
| wavelet-HL_glrlm_RunLengthNonUniformity | 0.000907956 | 0.00104019 | 0.13485163 | 3 | 0.006868384 | -0.00505247 |
| original_glcm_DifferenceVariance | 0.000907956 | 0.00039316 | 0.02859548 | 3 | 0.003160786 | -0.00134487 |
| gradient_ngtdm_Coarseness | 0.000907956 | 0.00128905 | 0.17340137 | 3 | 0.008294354 | -0.00647844 |
| wavelet-HH_glcm_ClusterShade | 0.000907956 | 0.00098289 | 0.12536568 | 3 | 0.006540031 | -0.00472412 |
| square_firstorder_10Percentile | 0.000907956 | 0.00241559 | 0.29091665 | 3 | 0.014749576 | -0.01293366 |
| logarithm_glcm_Imc2 | 0.000907956 | 0.00199505 | 0.25656775 | 3 | 0.01233982 | -0.01052391 |
| wavelet-LH_glrlm_RunEntropy | 0.000907956 | 0.00167957 | 0.22397378 | 3 | 0.010532051 | -0.00871614 |
| wavelet-HH_glrlm_RunVariance | 0.000851209 | 0.00029487 | 0.01887478 | 3 | 0.002540831 | -0.00083841 |
| gradient_glcm_Idm | 0.000794461 | 0.00153533 | 0.23234831 | 3 | 0.009592044 | -0.00800312 |
| original_shape2D_PerimeterSurfaceRatio | 0.000794461 | 0.00078631 | 0.11111111 | 3 | 0.005300122 | -0.0037112 |
| original_glrlm_RunPercentage | 0.000794461 | 0.00039316 | 0.03641368 | 3 | 0.003047292 | -0.00145837 |
| wavelet-LH_firstorder_Entropy | 0.000794461 | 0.00085687 | 0.12476061 | 3 | 0.005704391 | -0.00411547 |
| square_firstorder_InterquartileRange | 0.000794461 | 0.00119574 | 0.18441563 | 3 | 0.007646177 | -0.00605725 |
| gradient_glcm_Autocorrelation | 0.000794461 | 0.00019658 | 0.00990197 | 3 | 0.001920877 | -0.00033195 |
| gradient_glcm_ClusterTendency | 0.000794461 | 0.00373499 | 0.37395058 | 3 | 0.022196348 | -0.02060743 |
| gradient_gldm_LargeDependenceEmphasis | 0.000794461 | 0.00039316 | 0.03641368 | 3 | 0.003047292 | -0.00145837 |
| wavelet-LL_glszm_LowGrayLevelZoneEmphasis | 0.000794461 | 0.00157263 | 0.23692389 | 3 | 0.009805782 | -0.00821686 |
| wavelet-LL_glcm_Id | 0.000794461 | 0.00039316 | 0.03641368 | 3 | 0.003047292 | -0.00145837 |
| original_ngtdm_Strength | 0.000794461 | 0.00489081 | 0.40243843 | 3 | 0.028819342 | -0.02723042 |
| gradient_glcm_Imc1 | 0.000794461 | 0.00205234 | 0.28580348 | 3 | 0.01255458 | -0.01096566 |
| wavelet-LH_glcm_Contrast | 0.000794461 | 0.00137605 | 0.21132487 | 3 | 0.008679367 | -0.00709044 |
| wavelet-LH_glcm_DifferenceVariance | 0.000794461 | 0.00137605 | 0.21132487 | 3 | 0.008679367 | -0.00709044 |
| gradient_ngtdm_Complexity | 0.000794461 | 0.00231762 | 0.30644956 | 3 | 0.014074699 | -0.01248578 |
| original_glcm_ClusterProminence | 0.000794461 | 0.00137605 | 0.21132487 | 3 | 0.008679367 | -0.00709044 |
| original_gldm_SmallDependenceLowGrayLevelEmphasis | 0.000794461 | 0.00174723 | 0.25673318 | 3 | 0.010806258 | -0.00921734 |
| squareroot_glcm_JointEntropy | 0.000794461 | 0.00137605 | 0.21132487 | 3 | 0.008679367 | -0.00709044 |
| wavelet-LL_glcm_ClusterProminence | 0.000794461 | 0.00137605 | 0.21132487 | 3 | 0.008679367 | -0.00709044 |
| squareroot_firstorder_Variance | 0.000794461 | 0.00119574 | 0.18441563 | 3 | 0.007646177 | -0.00605725 |
| wavelet-LH_firstorder_Range | 0.000794461 | 0.0010945 | 0.1677947 | 3 | 0.007066075 | -0.00547715 |
| gradient_firstorder_MeanAbsoluteDeviation | 0.000794461 | 0.00264469 | 0.32735773 | 3 | 0.015948826 | -0.0143599 |
| wavelet-LH_firstorder_Kurtosis | 0.000794461 | 0.00085687 | 0.12476061 | 3 | 0.005704391 | -0.00411547 |
| wavelet-LH_glszm_GrayLevelVariance | 0.000680967 | 0.00090084 | 0.16031689 | 3 | 0.005842849 | -0.00448092 |
| wavelet-LL_glrlm_ShortRunEmphasis | 0.000680967 | 0.00034048 | 0.03708995 | 3 | 0.002631975 | -0.00127004 |
| original_glcm_MaximumProbability | 0.000680967 | 0.00379146 | 0.39258277 | 3 | 0.022406474 | -0.02104454 |
| gradient_gldm_LargeDependenceLowGrayLevelEmphasis | 0.000680967 | 0.00034048 | 0.03708995 | 3 | 0.002631975 | -0.00127004 |
| gradient_glcm_Id | 0.000680967 | 0.00034048 | 0.03708995 | 3 | 0.002631975 | -0.00127004 |
| logarithm_glrlm_RunEntropy | 0.000680967 | 0.00090084 | 0.16031689 | 3 | 0.005842849 | -0.00448092 |
| original_glcm_Idm | 0.000680967 | 0.00034048 | 0.03708995 | 3 | 0.002631975 | -0.00127004 |
| wavelet-LL_glcm_JointEntropy | 0.000680967 | 0 | 0.5 | 3 | 0.000680967 | 0.000680967 |
| wavelet-HL_glrlm_GrayLevelVariance | 0.000680967 | 0.00122763 | 0.21902426 | 3 | 0.007715427 | -0.00635349 |
| gradient_glcm_MaximumProbability | 0.000680967 | 0.00034048 | 0.03708995 | 3 | 0.002631975 | -0.00127004 |
| wavelet-LL_firstorder_Uniformity | 0.000680967 | 0.0022327 | 0.32503645 | 3 | 0.013474583 | -0.01211265 |
| original_firstorder_InterquartileRange | 0.000680967 | 0 | 0.5 | 3 | 0.000680967 | 0.000680967 |
| gradient_glrlm_RunPercentage | 0.000680967 | 0.00034048 | 0.03708995 | 3 | 0.002631975 | -0.00127004 |
| gradient_glrlm_ShortRunHighGrayLevelEmphasis | 0.000680967 | 0.00122763 | 0.21902426 | 3 | 0.007715427 | -0.00635349 |
| logarithm_glcm_ClusterTendency | 0.000680967 | 0 | 0.5 | 3 | 0.000680967 | 0.000680967 |
| wavelet-LL_glcm_Idn | 0.000680967 | 0.00156029 | 0.26429774 | 3 | 0.009621609 | -0.00825968 |
| squareroot_glcm_InverseVariance | 0.000680967 | 0.00148413 | 0.25505103 | 3 | 0.009185214 | -0.00782328 |
| wavelet-LH_glcm_DifferenceEntropy | 0.000680967 | 0.0017692 | 0.28679928 | 3 | 0.010818703 | -0.00945677 |
| wavelet-LH_gldm_DependenceVariance | 0.000680967 | 0.00491052 | 0.41627816 | 3 | 0.028818806 | -0.02745687 |
| squareroot_glszm_SmallAreaLowGrayLevelEmphasis | 0.000680967 | 0.00156029 | 0.26429774 | 3 | 0.009621609 | -0.00825968 |
| squareroot_gldm_SmallDependenceLowGrayLevelEmphasis | 0.000680967 | 0.00156029 | 0.26429774 | 3 | 0.009621609 | -0.00825968 |
| wavelet-HL_glszm_GrayLevelNonUniformity | 0.000680967 | 0.00117947 | 0.21132487 | 3 | 0.007439457 | -0.00607752 |
| wavelet-LH_gldm_SmallDependenceHighGrayLevelEmphasis | 0.000680967 | 0.00257059 | 0.34569665 | 3 | 0.015410755 | -0.01404882 |
| wavelet-HH_gldm_SmallDependenceEmphasis | 0.000680967 | 0 | 0.5 | 3 | 0.000680967 | 0.000680967 |
| logarithm_firstorder_Energy | 0.000680967 | 0 | 0.5 | 3 | 0.000680967 | 0.000680967 |
| wavelet-HH_glszm_ZonePercentage | 0.000680967 | 0 | 0.5 | 3 | 0.000680967 | 0.000680967 |
| original_glcm_Id | 0.000567472 | 0.00019658 | 0.01887478 | 3 | 0.001693888 | -0.00055894 |
| wavelet-LL_gldm_SmallDependenceEmphasis | 0.000567472 | 0.0005201 | 0.09967962 | 3 | 0.003547687 | -0.00241274 |
| wavelet-LL_glcm_DifferenceAverage | 0.000567472 | 0.0005201 | 0.09967962 | 3 | 0.003547687 | -0.00241274 |
| original_gldm_SmallDependenceEmphasis | 0.000567472 | 0.0005201 | 0.09967962 | 3 | 0.003547687 | -0.00241274 |
| wavelet-LH_firstorder_Uniformity | 0.000567472 | 0.0005201 | 0.09967962 | 3 | 0.003547687 | -0.00241274 |
| wavelet-LL_glszm_ZonePercentage | 0.000567472 | 0.0005201 | 0.09967962 | 3 | 0.003547687 | -0.00241274 |
| original_glcm_DifferenceEntropy | 0.000567472 | 0.0005201 | 0.09967962 | 3 | 0.003547687 | -0.00241274 |
| squareroot_glszm_ZoneEntropy | 0.000567472 | 0.00167957 | 0.30882022 | 3 | 0.010191567 | -0.00905662 |
| gradient_glrlm_RunEntropy | 0.000567472 | 0.00153533 | 0.29380348 | 3 | 0.009365055 | -0.00823011 |
| wavelet-LL_glcm_MCC | 0.000567472 | 0.0069806 | 0.45046348 | 3 | 0.040567105 | -0.03943216 |
| square_gldm_DependenceEntropy | 0.000567472 | 0.0005201 | 0.09967962 | 3 | 0.003547687 | -0.00241274 |
| original_glcm_JointEnergy | 0.000567472 | 0.00527841 | 0.4347283 | 3 | 0.030813343 | -0.0296784 |
| wavelet-LL_glrlm_LongRunLowGrayLevelEmphasis | 0.000567472 | 0.00104019 | 0.22222222 | 3 | 0.006527901 | -0.00539296 |
| original_ngtdm_Coarseness | 0.000567472 | 0.00141755 | 0.27988727 | 3 | 0.008690167 | -0.00755522 |
| gradient_firstorder_Maximum | 0.000567472 | 0.00411877 | 0.41680521 | 3 | 0.024168489 | -0.02303354 |
| original_glrlm_RunLengthNonUniformityNormalized | 0.000453978 | 0.00039316 | 0.09175171 | 3 | 0.002706808 | -0.00179885 |
| original_glrlm_ShortRunEmphasis | 0.000453978 | 0.00039316 | 0.09175171 | 3 | 0.002706808 | -0.00179885 |
| square_firstorder_Entropy | 0.000453978 | 0.00039316 | 0.09175171 | 3 | 0.002706808 | -0.00179885 |
| square_firstorder_RootMeanSquared | 0.000453978 | 0.00039316 | 0.09175171 | 3 | 0.002706808 | -0.00179885 |
| square_glcm_InverseVariance | 0.000453978 | 0.00039316 | 0.09175171 | 3 | 0.002706808 | -0.00179885 |
| original_firstorder_Entropy | 0.000453978 | 0.00039316 | 0.09175171 | 3 | 0.002706808 | -0.00179885 |
| square_glcm_SumEntropy | 0.000453978 | 0.00039316 | 0.09175171 | 3 | 0.002706808 | -0.00179885 |
| square_glrlm_ShortRunEmphasis | 0.000453978 | 0.00039316 | 0.09175171 | 3 | 0.002706808 | -0.00179885 |
| square_glcm_MaximumProbability | 0.000453978 | 0.00039316 | 0.09175171 | 3 | 0.002706808 | -0.00179885 |
| square_glcm_JointEnergy | 0.000453978 | 0.00039316 | 0.09175171 | 3 | 0.002706808 | -0.00179885 |
| logarithm_firstorder_Minimum | 0.000453978 | 0.00070877 | 0.1913933 | 3 | 0.004515325 | -0.00360737 |
| original_glcm_SumEntropy | 0.000453978 | 0.00039316 | 0.09175171 | 3 | 0.002706808 | -0.00179885 |
| wavelet-HL_firstorder_90Percentile | 0.000453978 | 0.00039316 | 0.09175171 | 3 | 0.002706808 | -0.00179885 |
| wavelet-HH_firstorder_90Percentile | 0.000453978 | 0.00039316 | 0.09175171 | 3 | 0.002706808 | -0.00179885 |
| wavelet-HL_glcm_Idmn | 0.000453978 | 0.0005201 | 0.13485163 | 3 | 0.003434192 | -0.00252624 |
| square_glcm_JointEntropy | 0.000453978 | 0.00039316 | 0.09175171 | 3 | 0.002706808 | -0.00179885 |
| gradient_firstorder_Median | 0.000453978 | 0.00039316 | 0.09175171 | 3 | 0.002706808 | -0.00179885 |
| square_glcm_DifferenceEntropy | 0.000453978 | 0.00039316 | 0.09175171 | 3 | 0.002706808 | -0.00179885 |
| wavelet-HH_gldm_LargeDependenceEmphasis | 0.000453978 | 0.00039316 | 0.09175171 | 3 | 0.002706808 | -0.00179885 |
| original_glrlm_LongRunEmphasis | 0.000453978 | 0.00357643 | 0.42319046 | 3 | 0.020947304 | -0.02003935 |
| square_gldm_LargeDependenceLowGrayLevelEmphasis | 0.000453978 | 0.00039316 | 0.09175171 | 3 | 0.002706808 | -0.00179885 |
| logarithm_gldm_SmallDependenceLowGrayLevelEmphasis | 0.000453978 | 0.00193607 | 0.36198689 | 3 | 0.01154788 | -0.01063992 |
| square_gldm_SmallDependenceEmphasis | 0.000453978 | 0.00039316 | 0.09175171 | 3 | 0.002706808 | -0.00179885 |
| squareroot_ngtdm_Coarseness | 0.000453978 | 0.00174723 | 0.34838039 | 3 | 0.010465774 | -0.00955782 |
| exponential_gldm_LargeDependenceHighGrayLevelEmphasis | 0.000453978 | 0.00153533 | 0.32974869 | 3 | 0.009251561 | -0.00834361 |
| wavelet-HH_ngtdm_Complexity | 0.000453978 | 0.00019658 | 0.02859548 | 3 | 0.001580393 | -0.00067244 |
| logarithm_glszm_SizeZoneNonUniformityNormalized | 0.000453978 | 0.00104019 | 0.26429774 | 3 | 0.006414406 | -0.00550645 |
| wavelet-HL_firstorder_10Percentile | 0.000453978 | 0.00241559 | 0.38784557 | 3 | 0.014295598 | -0.01338764 |
| exponential_gldm_SmallDependenceEmphasis | 0.000453978 | 0.00205234 | 0.36925591 | 3 | 0.012214097 | -0.01130614 |
| gradient_glcm_DifferenceVariance | 0.000453978 | 0.00019658 | 0.02859548 | 3 | 0.001580393 | -0.00067244 |
| wavelet-HH_ngtdm_Strength | 0.000453978 | 0.0025781 | 0.39459075 | 3 | 0.015226773 | -0.01431882 |
| wavelet-HL_gldm_SmallDependenceEmphasis | 0.000453978 | 0.00039316 | 0.09175171 | 3 | 0.002706808 | -0.00179885 |
| wavelet-HL_firstorder_MeanAbsoluteDeviation | 0.000340483 | 0.00034048 | 0.11270167 | 3 | 0.002291492 | -0.00161053 |
| logarithm_glszm_LargeAreaLowGrayLevelEmphasis | 0.000340483 | 0 | 0.5 | 3 | 0.000340483 | 0.000340483 |
| original_glrlm_GrayLevelNonUniformityNormalized | 0.000340483 | 0.00034048 | 0.11270167 | 3 | 0.002291492 | -0.00161053 |
| wavelet-HL_firstorder_Uniformity | 0.000340483 | 0 | 0.5 | 3 | 0.000340483 | 0.000340483 |
| wavelet-LH_glrlm_LongRunEmphasis | 0.000340483 | 0.00058974 | 0.21132487 | 3 | 0.003719729 | -0.00303876 |
| wavelet-HL_glrlm_RunPercentage | 0.000340483 | 0 | 0.5 | 3 | 0.000340483 | 0.000340483 |
| wavelet-LL_glrlm_GrayLevelNonUniformityNormalized | 0.000340483 | 0.00034048 | 0.11270167 | 3 | 0.002291492 | -0.00161053 |
| logarithm_firstorder_InterquartileRange | 0.000340483 | 0.00156029 | 0.37090056 | 3 | 0.009281126 | -0.00860016 |
| logarithm_gldm_DependenceEntropy | 0.000340483 | 0.00148413 | 0.36474956 | 3 | 0.008844731 | -0.00816376 |
| logarithm_glrlm_ShortRunLowGrayLevelEmphasis | 0.000340483 | 0.00058974 | 0.21132487 | 3 | 0.003719729 | -0.00303876 |
| exponential_gldm_DependenceVariance | 0.000340483 | 0.00090084 | 0.28995799 | 3 | 0.005502366 | -0.0048214 |
| logarithm_glrlm_LowGrayLevelRunEmphasis | 0.000340483 | 0.0022327 | 0.40820149 | 3 | 0.013134099 | -0.01245313 |
| wavelet-HH_firstorder_Uniformity | 0.000226989 | 0.00070877 | 0.31742581 | 3 | 0.004288336 | -0.00383436 |
| wavelet-LL_gldm_LargeDependenceEmphasis | 0.000226989 | 0.00137605 | 0.40098525 | 3 | 0.008111894 | -0.00765792 |
| wavelet-HL_glszm_ZonePercentage | 0.000226989 | 0.00019658 | 0.09175171 | 3 | 0.001353404 | -0.00089943 |
| original_glcm_JointEntropy | 0.000226989 | 0.00141755 | 0.40377496 | 3 | 0.008349684 | -0.00789571 |
| original_gldm_LargeDependenceLowGrayLevelEmphasis | 0.000226989 | 0.00019658 | 0.09175171 | 3 | 0.001353404 | -0.00089943 |
| square_glrlm_GrayLevelNonUniformityNormalized | 0.000226989 | 0.0005201 | 0.26429774 | 3 | 0.003207203 | -0.00275323 |
| gradient_glszm_SmallAreaLowGrayLevelEmphasis | 0.000226989 | 0.00373499 | 0.46288652 | 3 | 0.021628875 | -0.0211749 |
| wavelet-HL_glcm_ClusterTendency | 0.000226989 | 0.00039316 | 0.21132487 | 3 | 0.002479819 | -0.00202584 |
| wavelet-HL_firstorder_Variance | 0.000226989 | 0.00039316 | 0.21132487 | 3 | 0.002479819 | -0.00202584 |
| wavelet-HL_glcm_MaximumProbability | 0.000226989 | 0.00039316 | 0.21132487 | 3 | 0.002479819 | -0.00202584 |
| wavelet-LH_firstorder_Maximum | 0.000226989 | 0.00098289 | 0.36391724 | 3 | 0.005859064 | -0.00540509 |
| wavelet-HL_glrlm_RunLengthNonUniformityNormalized | 0.000226989 | 0.00019658 | 0.09175171 | 3 | 0.001353404 | -0.00089943 |
| gradient_glrlm_LongRunHighGrayLevelEmphasis | 0.000226989 | 0.00019658 | 0.09175171 | 3 | 0.001353404 | -0.00089943 |
| wavelet-HL_glcm_Id | 0.000226989 | 0.00019658 | 0.09175171 | 3 | 0.001353404 | -0.00089943 |
| wavelet-HL_glcm_Idm | 0.000226989 | 0.00019658 | 0.09175171 | 3 | 0.001353404 | -0.00089943 |
| wavelet-HL_firstorder_Energy | 0.000226989 | 0.00039316 | 0.21132487 | 3 | 0.002479819 | -0.00202584 |
| wavelet-HL_glrlm_ShortRunEmphasis | 0.000226989 | 0.00019658 | 0.09175171 | 3 | 0.001353404 | -0.00089943 |
| gradient_glcm_Idn | 0.000226989 | 0.00039316 | 0.21132487 | 3 | 0.002479819 | -0.00202584 |
| wavelet-LH_glrlm_RunVariance | 0.000226989 | 0.00039316 | 0.21132487 | 3 | 0.002479819 | -0.00202584 |
| logarithm_glszm_GrayLevelVariance | 0.000226989 | 0.00205234 | 0.43288439 | 3 | 0.011987108 | -0.01153313 |
| wavelet-LL_gldm_DependenceVariance | 0.000226989 | 0.00443065 | 0.46868879 | 3 | 0.025615095 | -0.02516112 |
| wavelet-HH_glszm_GrayLevelNonUniformity | 0.000226989 | 0.0005201 | 0.26429774 | 3 | 0.003207203 | -0.00275323 |
| exponential_firstorder_Maximum | 0.000226989 | 0.0005201 | 0.26429774 | 3 | 0.003207203 | -0.00275323 |
| original_glcm_ClusterShade | 0.000226989 | 0.00039316 | 0.21132487 | 3 | 0.002479819 | -0.00202584 |
| gradient_glszm_ZoneEntropy | 0.000226989 | 0.00205234 | 0.43288439 | 3 | 0.011987108 | -0.01153313 |
| exponential_gldm_DependenceNonUniformityNormalized | 0.000226989 | 0.00078631 | 0.33333333 | 3 | 0.004732649 | -0.00427867 |
| wavelet-HH_firstorder_Entropy | 0.000113494 | 0.00070877 | 0.40377496 | 3 | 0.004174842 | -0.00394785 |
| square_gldm_GrayLevelVariance | 0.000113494 | 0.00019658 | 0.21132487 | 3 | 0.00123991 | -0.00101292 |
| squareroot_ngtdm_Busyness | 0.000113494 | 0.0005201 | 0.37090056 | 3 | 0.003093709 | -0.00286672 |
| squareroot_gldm_LargeDependenceHighGrayLevelEmphasis | 0.000113494 | 0.00019658 | 0.21132487 | 3 | 0.00123991 | -0.00101292 |
| gradient_glcm_JointAverage | 0.000113494 | 0.00019658 | 0.21132487 | 3 | 0.00123991 | -0.00101292 |
| original_gldm_DependenceEntropy | 0.000113494 | 0.00327171 | 0.47877618 | 3 | 0.018860778 | -0.01863379 |
| squareroot_glcm_Contrast | 0.000113494 | 0.00019658 | 0.21132487 | 3 | 0.00123991 | -0.00101292 |
| original_glszm_GrayLevelVariance | 0.000113494 | 0.00221532 | 0.46868879 | 3 | 0.012807548 | -0.01258056 |
| wavelet-HL_firstorder_RobustMeanAbsoluteDeviation | 0.000113494 | 0.00019658 | 0.21132487 | 3 | 0.00123991 | -0.00101292 |
| wavelet-LH_glcm_Idmn | 0.000113494 | 0.00293554 | 0.47635081 | 3 | 0.016934459 | -0.01670747 |
| original_gldm_LargeDependenceHighGrayLevelEmphasis | 0.000113494 | 0.00260049 | 0.47331197 | 3 | 0.015014565 | -0.01478758 |
| logarithm_glcm_Idmn | 0.000113494 | 0.00231762 | 0.47006578 | 3 | 0.013393732 | -0.01316674 |
| wavelet-HL_glcm_JointEnergy | 0.000113494 | 0.0005201 | 0.37090056 | 3 | 0.003093709 | -0.00286672 |
| wavelet-LL_gldm_DependenceEntropy | 0.000113494 | 0.00321812 | 0.47842341 | 3 | 0.018553704 | -0.01832672 |
| logarithm_firstorder_Range | 3.70E-17 | 0.00207108 | 0.5 | 3 | 0.011867519 | -0.01186752 |
| original_glrlm_LongRunHighGrayLevelEmphasis | 3.70E-17 | 0.00294867 | 0.5 | 3 | 0.016896226 | -0.01689623 |
| square_glszm_GrayLevelNonUniformityNormalized | 3.70E-17 | 0.00491052 | 0.5 | 3 | 0.028137839 | -0.02813784 |
| exponential_firstorder_RootMeanSquared | 3.70E-17 | 0.00117947 | 0.5 | 3 | 0.00675849 | -0.00675849 |
| square_glcm_ClusterProminence | 0 | 0 | 0.5 | 3 | 0 | 0 |
| square_glcm_Contrast | 0 | 0 | 0.5 | 3 | 0 | 0 |
| square_glcm_Autocorrelation | 0 | 0 | 0.5 | 3 | 0 | 0 |
| square_glcm_SumSquares | 0 | 0 | 0.5 | 3 | 0 | 0 |
| square_firstorder_RobustMeanAbsoluteDeviation | 0 | 0 | 0.5 | 3 | 0 | 0 |
| square_firstorder_Variance | 0 | 0 | 0.5 | 3 | 0 | 0 |
| square_glcm_ClusterShade | 0 | 0 | 0.5 | 3 | 0 | 0 |
| square_gldm_HighGrayLevelEmphasis | 0 | 0 | 0.5 | 3 | 0 | 0 |
| wavelet-LH_ngtdm_Complexity | 0 | 0 | 0.5 | 3 | 0 | 0 |
| wavelet-HH_glszm_LargeAreaLowGrayLevelEmphasis | 0 | 0 | 0.5 | 3 | 0 | 0 |
| wavelet-HH_ngtdm_Busyness | 0 | 0 | 0.5 | 3 | 0 | 0 |
| wavelet-HL_firstorder_Median | 0 | 0.00058974 | 0.5 | 3 | 0.003379245 | -0.00337925 |
| wavelet-HL_glcm_DifferenceAverage | 0 | 0 | 0.5 | 3 | 0 | 0 |
| wavelet-LH_glcm_ClusterProminence | 0 | 0 | 0.5 | 3 | 0 | 0 |
| wavelet-LH_glszm_LargeAreaLowGrayLevelEmphasis | 0 | 0 | 0.5 | 3 | 0 | 0 |
| wavelet-LL_glcm_ClusterShade | 0 | 0 | 0.5 | 3 | 0 | 0 |
| square_gldm_LargeDependenceHighGrayLevelEmphasis | 0 | 0 | 0.5 | 3 | 0 | 0 |
| wavelet-LL_gldm_DependenceNonUniformityNormalized | 0 | 0.00034048 | 0.5 | 3 | 0.001951008 | -0.00195101 |
| wavelet-LL_gldm_LowGrayLevelEmphasis | 0 | 0 | 0.5 | 3 | 0 | 0 |
| wavelet-LL_glszm_LargeAreaEmphasis | 0 | 0 | 0.5 | 3 | 0 | 0 |
| wavelet-LL_glszm_LargeAreaHighGrayLevelEmphasis | 0 | 0 | 0.5 | 3 | 0 | 0 |
| wavelet-LL_glszm_ZoneVariance | 0 | 0 | 0.5 | 3 | 0 | 0 |
| wavelet-HH_glcm_InverseVariance | 0 | 0 | 0.5 | 3 | 0 | 0 |
| wavelet-HH_glcm_ClusterProminence | 0 | 0 | 0.5 | 3 | 0 | 0 |
| wavelet-HH_firstorder_MeanAbsoluteDeviation | 0 | 0.00102145 | 0.5 | 3 | 0.005853024 | -0.00585302 |
| wavelet-HH_firstorder_Kurtosis | 0 | 0.00245526 | 0.5 | 3 | 0.01406892 | -0.01406892 |
| squareroot_glszm_ZonePercentage | 0 | 0 | 0.5 | 3 | 0 | 0 |
| squareroot_glszm_LargeAreaHighGrayLevelEmphasis | 0 | 0 | 0.5 | 3 | 0 | 0 |
| squareroot_glszm_LargeAreaEmphasis | 0 | 0 | 0.5 | 3 | 0 | 0 |
| squareroot_glszm_GrayLevelNonUniformityNormalized | 0 | 0.00102145 | 0.5 | 3 | 0.005853024 | -0.00585302 |
| squareroot_glrlm_LongRunEmphasis | 0 | 0.00090084 | 0.5 | 3 | 0.005161882 | -0.00516188 |
| squareroot_gldm_SmallDependenceEmphasis | 0 | 0 | 0.5 | 3 | 0 | 0 |
| squareroot_gldm_DependenceNonUniformityNormalized | 0 | 0 | 0.5 | 3 | 0 | 0 |
| squareroot_glcm_ClusterShade | 0 | 0 | 0.5 | 3 | 0 | 0 |
| square_ngtdm_Contrast | 0 | 0 | 0.5 | 3 | 0 | 0 |
| square_glrlm_ShortRunHighGrayLevelEmphasis | 0 | 0.00058974 | 0.5 | 3 | 0.003379245 | -0.00337925 |
| square_glrlm_LongRunEmphasis | 0 | 0.00058974 | 0.5 | 3 | 0.003379245 | -0.00337925 |
| square_glrlm_HighGrayLevelRunEmphasis | 0 | 0 | 0.5 | 3 | 0 | 0 |
| square_firstorder_Minimum | 0 | 0 | 0.5 | 3 | 0 | 0 |
| square_firstorder_Mean | 0 | 0 | 0.5 | 3 | 0 | 0 |
| exponential_glcm_DifferenceVariance | 0 | 0 | 0.5 | 3 | 0 | 0 |
| exponential_glcm_JointEntropy | 0 | 0 | 0.5 | 3 | 0 | 0 |
| exponential_glcm_JointEnergy | 0 | 0 | 0.5 | 3 | 0 | 0 |
| exponential_glcm_JointAverage | 0 | 0 | 0.5 | 3 | 0 | 0 |
| exponential_glcm_InverseVariance | 0 | 0 | 0.5 | 3 | 0 | 0 |
| exponential_glcm_Imc2 | 0 | 0 | 0.5 | 3 | 0 | 0 |
| exponential_glcm_Imc1 | 0 | 0 | 0.5 | 3 | 0 | 0 |
| exponential_glcm_Idn | 0 | 0 | 0.5 | 3 | 0 | 0 |
| exponential_glcm_Idmn | 0 | 0 | 0.5 | 3 | 0 | 0 |
| exponential_glcm_Idm | 0 | 0 | 0.5 | 3 | 0 | 0 |
| exponential_glcm_Id | 0 | 0 | 0.5 | 3 | 0 | 0 |
| exponential_glcm_DifferenceEntropy | 0 | 0 | 0.5 | 3 | 0 | 0 |
| exponential_glcm_MaximumProbability | 0 | 0 | 0.5 | 3 | 0 | 0 |
| exponential_glcm_DifferenceAverage | 0 | 0 | 0.5 | 3 | 0 | 0 |
| exponential_glcm_Correlation | 0 | 0 | 0.5 | 3 | 0 | 0 |
| exponential_glcm_Contrast | 0 | 0 | 0.5 | 3 | 0 | 0 |
| exponential_glcm_ClusterTendency | 0 | 0 | 0.5 | 3 | 0 | 0 |
| exponential_glcm_ClusterShade | 0 | 0 | 0.5 | 3 | 0 | 0 |
| exponential_glcm_ClusterProminence | 0 | 0 | 0.5 | 3 | 0 | 0 |
| exponential_glcm_Autocorrelation | 0 | 0 | 0.5 | 3 | 0 | 0 |
| exponential_firstorder_Variance | 0 | 0 | 0.5 | 3 | 0 | 0 |
| exponential_firstorder_Uniformity | 0 | 0 | 0.5 | 3 | 0 | 0 |
| exponential_firstorder_Range | 0 | 0 | 0.5 | 3 | 0 | 0 |
| exponential_glcm_MCC | 0 | 0 | 0.5 | 3 | 0 | 0 |
| logarithm_gldm_LargeDependenceLowGrayLevelEmphasis | 0 | 0 | 0.5 | 3 | 0 | 0 |
| original_gldm_LowGrayLevelEmphasis | 0 | 0 | 0.5 | 3 | 0 | 0 |
| exponential_glszm_GrayLevelVariance | 0 | 0 | 0.5 | 3 | 0 | 0 |
| exponential_ngtdm_Contrast | 0 | 0 | 0.5 | 3 | 0 | 0 |
| exponential_ngtdm_Strength | 0 | 0 | 0.5 | 3 | 0 | 0 |
| exponential_ngtdm_Busyness | 0 | 0 | 0.5 | 3 | 0 | 0 |
| exponential_glszm_ZoneVariance | 0 | 0 | 0.5 | 3 | 0 | 0 |
| exponential_glszm_ZoneEntropy | 0 | 0 | 0.5 | 3 | 0 | 0 |
| exponential_glszm_SmallAreaEmphasis | 0 | 0 | 0.5 | 3 | 0 | 0 |
| exponential_glszm_SizeZoneNonUniformityNormalized | 0 | 0 | 0.5 | 3 | 0 | 0 |
| exponential_glszm_SizeZoneNonUniformity | 0 | 0 | 0.5 | 3 | 0 | 0 |
| exponential_glszm_LowGrayLevelZoneEmphasis | 0 | 0 | 0.5 | 3 | 0 | 0 |
| exponential_glszm_HighGrayLevelZoneEmphasis | 0 | 0 | 0.5 | 3 | 0 | 0 |
| exponential_glszm_GrayLevelNonUniformityNormalized | 0 | 0 | 0.5 | 3 | 0 | 0 |
| exponential_glcm_SumEntropy | 0 | 0 | 0.5 | 3 | 0 | 0 |
| exponential_glszm_GrayLevelNonUniformity | 0 | 0 | 0.5 | 3 | 0 | 0 |
| exponential_glrlm_LowGrayLevelRunEmphasis | 0 | 0 | 0.5 | 3 | 0 | 0 |
| exponential_glrlm_HighGrayLevelRunEmphasis | 0 | 0 | 0.5 | 3 | 0 | 0 |
| exponential_glrlm_GrayLevelVariance | 0 | 0 | 0.5 | 3 | 0 | 0 |
| exponential_glrlm_GrayLevelNonUniformityNormalized | 0 | 0 | 0.5 | 3 | 0 | 0 |
| exponential_gldm_LowGrayLevelEmphasis | 0 | 0 | 0.5 | 3 | 0 | 0 |
| exponential_gldm_HighGrayLevelEmphasis | 0 | 0 | 0.5 | 3 | 0 | 0 |
| exponential_gldm_GrayLevelVariance | 0 | 0 | 0.5 | 3 | 0 | 0 |
| exponential_glcm_SumSquares | 0 | 0 | 0.5 | 3 | 0 | 0 |
| exponential_ngtdm_Coarseness | 0 | 0 | 0.5 | 3 | 0 | 0 |
| exponential_firstorder_MeanAbsoluteDeviation | 0 | 0 | 0.5 | 3 | 0 | 0 |
| exponential_ngtdm_Complexity | 0 | 0 | 0.5 | 3 | 0 | 0 |
| subsolid | 0 | 0 | 0.5 | 3 | 0 | 0 |
| calcification | 0 | 0 | 0.5 | 3 | 0 | 0 |
| original_glszm_LargeAreaHighGrayLevelEmphasis | 0 | 0.00034048 | 0.5 | 3 | 0.001951008 | -0.00195101 |
| original_glszm_LowGrayLevelZoneEmphasis | 0 | 0 | 0.5 | 3 | 0 | 0 |
| original_glrlm_ShortRunLowGrayLevelEmphasis | 0 | 0 | 0.5 | 3 | 0 | 0 |
| original_glrlm_LowGrayLevelRunEmphasis | 0 | 0 | 0.5 | 3 | 0 | 0 |
| exponential_firstorder_Entropy | 0 | 0 | 0.5 | 3 | 0 | 0 |
| spiculation | 0 | 0 | 0.5 | 3 | 0 | 0 |
| wavelet-HH_glrlm_GrayLevelNonUniformityNormalized | -3.70E-17 | 0.00148413 | 0.5 | 3 | 0.008504247 | -0.00850425 |
| wavelet-LH_glcm_Imc2 | -3.70E-17 | 0.00238338 | 0.5 | 3 | 0.013657057 | -0.01365706 |
| logarithm_glcm_ClusterShade | -0.00011349 | 0.0005201 | 0.62909945 | 3 | 0.00286672 | -0.00309371 |
| original_gldm_DependenceNonUniformity | -0.00011349 | 0.00098289 | 0.570014 | 3 | 0.005518581 | -0.00574557 |
| exponential_firstorder_Kurtosis | -0.00011349 | 0.00167957 | 0.54123931 | 3 | 0.0095106 | -0.00973759 |
| wavelet-LH_glszm_SizeZoneNonUniformity | -0.00011349 | 0.00364068 | 0.51907619 | 3 | 0.020748005 | -0.02097499 |
| square_glrlm_LowGrayLevelRunEmphasis | -0.00011349 | 0.0005201 | 0.62909945 | 3 | 0.00286672 | -0.00309371 |
| wavelet-HH_glrlm_GrayLevelVariance | -0.00011349 | 0.00285546 | 0.52431083 | 3 | 0.01624863 | -0.01647562 |
| squareroot_firstorder_Energy | -0.00011349 | 0.00104019 | 0.56622662 | 3 | 0.005846934 | -0.00607392 |
| gradient_glrlm_ShortRunEmphasis | -0.00011349 | 0.00231762 | 0.52993422 | 3 | 0.013166743 | -0.01339373 |
| squareroot_gldm_GrayLevelNonUniformity | -0.00011349 | 0.00019658 | 0.78867514 | 3 | 0.001012921 | -0.00123991 |
| wavelet-LH_glszm_LargeAreaHighGrayLevelEmphasis | -0.00011349 | 0.00019658 | 0.78867514 | 3 | 0.001012921 | -0.00123991 |
| logarithm_firstorder_Median | -0.00011349 | 0.00085687 | 0.58006408 | 3 | 0.004796435 | -0.00502342 |
| gradient_glrlm_RunVariance | -0.00011349 | 0.00019658 | 0.78867514 | 3 | 0.001012921 | -0.00123991 |
| wavelet-HL_gldm_LargeDependenceLowGrayLevelEmphasis | -0.00011349 | 0.00019658 | 0.78867514 | 3 | 0.001012921 | -0.00123991 |
| gradient_glrlm_LongRunEmphasis | -0.00011349 | 0.00019658 | 0.78867514 | 3 | 0.001012921 | -0.00123991 |
| square_glcm_ClusterTendency | -0.00011349 | 0.00019658 | 0.78867514 | 3 | 0.001012921 | -0.00123991 |
| gradient_gldm_DependenceNonUniformity | -0.00011349 | 0.00019658 | 0.78867514 | 3 | 0.001012921 | -0.00123991 |
| square_firstorder_Median | -0.00011349 | 0.00308947 | 0.52247333 | 3 | 0.017589508 | -0.0178165 |
| original_glszm_LargeAreaEmphasis | -0.00011349 | 0.00019658 | 0.78867514 | 3 | 0.001012921 | -0.00123991 |
| wavelet-LH_glszm_LargeAreaEmphasis | -0.00011349 | 0.00019658 | 0.78867514 | 3 | 0.001012921 | -0.00123991 |
| exponential_firstorder_Minimum | -0.00011349 | 0.00019658 | 0.78867514 | 3 | 0.001012921 | -0.00123991 |
| exponential_firstorder_Median | -0.00011349 | 0.00085687 | 0.58006408 | 3 | 0.004796435 | -0.00502342 |
| wavelet-LL_ngtdm_Busyness | -0.00011349 | 0.00019658 | 0.78867514 | 3 | 0.001012921 | -0.00123991 |
| wavelet-HH_glrlm_RunEntropy | -0.00011349 | 0.00070877 | 0.59622505 | 3 | 0.003947853 | -0.00417484 |
| wavelet-LH_ngtdm_Busyness | -0.00011349 | 0.00019658 | 0.78867514 | 3 | 0.001012921 | -0.00123991 |
| square_glszm_HighGrayLevelZoneEmphasis | -0.00011349 | 0.00085687 | 0.58006408 | 3 | 0.004796435 | -0.00502342 |
| square_gldm_LowGrayLevelEmphasis | -0.00011349 | 0.00137605 | 0.55025189 | 3 | 0.007771411 | -0.0079984 |
| original_ngtdm_Busyness | -0.00011349 | 0.00019658 | 0.78867514 | 3 | 0.001012921 | -0.00123991 |
| wavelet-LL_glrlm_ShortRunLowGrayLevelEmphasis | -0.00011349 | 0.00085687 | 0.58006408 | 3 | 0.004796435 | -0.00502342 |
| wavelet-HL_glrlm_LongRunLowGrayLevelEmphasis | -0.00011349 | 0.00382697 | 0.51814885 | 3 | 0.021815467 | -0.02204246 |
| logarithm_gldm_GrayLevelNonUniformity | -0.00011349 | 0.00019658 | 0.78867514 | 3 | 0.001012921 | -0.00123991 |
| wavelet-HL_glszm_LargeAreaHighGrayLevelEmphasis | -0.00011349 | 0.00019658 | 0.78867514 | 3 | 0.001012921 | -0.00123991 |
| wavelet-LH_glcm_InverseVariance | -0.00011349 | 0.00193607 | 0.53580574 | 3 | 0.010980407 | -0.0112074 |
| wavelet-HL_glszm_SizeZoneNonUniformity | -0.00011349 | 0.00527841 | 0.51316245 | 3 | 0.030132376 | -0.03035937 |
| square_firstorder_Skewness | -0.00011349 | 0.0005201 | 0.62909945 | 3 | 0.00286672 | -0.00309371 |
| logarithm_glcm_DifferenceEntropy | -0.00011349 | 0.00171373 | 0.5404226 | 3 | 0.009706364 | -0.00993335 |
| wavelet-HH_glcm_Idm | -0.00011349 | 0.00137605 | 0.55025189 | 3 | 0.007771411 | -0.0079984 |
| wavelet-HH_glcm_Id | -0.00011349 | 0.00137605 | 0.55025189 | 3 | 0.007771411 | -0.0079984 |
| wavelet-HH_glcm_DifferenceEntropy | -0.00011349 | 0.00137605 | 0.55025189 | 3 | 0.007771411 | -0.0079984 |
| wavelet-LL_glszm_GrayLevelNonUniformityNormalized | -0.00022699 | 0.00141755 | 0.59622505 | 3 | 0.007895706 | -0.00834968 |
| gradient_glrlm_LongRunLowGrayLevelEmphasis | -0.00022699 | 0.00070877 | 0.68257419 | 3 | 0.003834358 | -0.00428834 |
| wavelet-HH_glszm_LargeAreaHighGrayLevelEmphasis | -0.00022699 | 0.00019658 | 0.90824829 | 3 | 0.000899426 | -0.0013534 |
| square_glrlm_LongRunHighGrayLevelEmphasis | -0.00022699 | 0.00019658 | 0.90824829 | 3 | 0.000899426 | -0.0013534 |
| squareroot_glcm_ClusterTendency | -0.00022699 | 0.00141755 | 0.59622505 | 3 | 0.007895706 | -0.00834968 |
| wavelet-HL_glszm_LargeAreaEmphasis | -0.00022699 | 0.00019658 | 0.90824829 | 3 | 0.000899426 | -0.0013534 |
| square_firstorder_Kurtosis | -0.00022699 | 0.00039316 | 0.78867514 | 3 | 0.002025841 | -0.00247982 |
| square_glszm_LargeAreaEmphasis | -0.00022699 | 0.00039316 | 0.78867514 | 3 | 0.002025841 | -0.00247982 |
| square_glszm_LargeAreaHighGrayLevelEmphasis | -0.00022699 | 0.00039316 | 0.78867514 | 3 | 0.002025841 | -0.00247982 |
| square_glszm_LargeAreaLowGrayLevelEmphasis | -0.00022699 | 0.00039316 | 0.78867514 | 3 | 0.002025841 | -0.00247982 |
| gradient_firstorder_Skewness | -0.00022699 | 0.00019658 | 0.90824829 | 3 | 0.000899426 | -0.0013534 |
| logarithm_gldm_DependenceNonUniformityNormalized | -0.00022699 | 0.00039316 | 0.78867514 | 3 | 0.002025841 | -0.00247982 |
| wavelet-HL_glszm_HighGrayLevelZoneEmphasis | -0.00022699 | 0.00335913 | 0.54123931 | 3 | 0.0190212 | -0.01947518 |
| original_firstorder_Uniformity | -0.00022699 | 0.00335913 | 0.54123931 | 3 | 0.0190212 | -0.01947518 |
| wavelet-HL_glcm_Autocorrelation | -0.00022699 | 0.00226705 | 0.56085806 | 3 | 0.012763463 | -0.01321744 |
| square_glrlm_GrayLevelVariance | -0.00034048 | 0 | 0.5 | 3 | -0.00034048 | -0.00034048 |
| exponential_glrlm_LongRunHighGrayLevelEmphasis | -0.00034048 | 0.00102145 | 0.68898224 | 3 | 0.005512541 | -0.00619351 |
| gradient_gldm_DependenceEntropy | -0.00034048 | 0.00180167 | 0.6127469 | 3 | 0.009983281 | -0.01066425 |
| logarithm_glcm_ClusterProminence | -0.00034048 | 0.00117947 | 0.66666667 | 3 | 0.006418007 | -0.00709897 |
| original_glrlm_LongRunLowGrayLevelEmphasis | -0.00034048 | 0 | 0.5 | 3 | -0.00034048 | -0.00034048 |
| gradient_firstorder_Kurtosis | -0.00034048 | 0.00034048 | 0.88729834 | 3 | 0.001610525 | -0.00229149 |
| wavelet-HL_gldm_HighGrayLevelEmphasis | -0.00034048 | 0.00335337 | 0.56170176 | 3 | 0.018874718 | -0.01955569 |
| logarithm_glcm_Imc1 | -0.00034048 | 0.00136193 | 0.64638501 | 3 | 0.007463549 | -0.00814452 |
| wavelet-HH_gldm_SmallDependenceHighGrayLevelEmphasis | -0.00034048 | 0.00235894 | 0.58703883 | 3 | 0.013176497 | -0.01385746 |
| exponential_gldm_SmallDependenceLowGrayLevelEmphasis | -0.00034048 | 0.00235894 | 0.58703883 | 3 | 0.013176497 | -0.01385746 |
| gradient_glcm_SumEntropy | -0.00045398 | 0.00167957 | 0.65713484 | 3 | 0.009170117 | -0.01007807 |
| wavelet-HL_gldm_SmallDependenceHighGrayLevelEmphasis | -0.00045398 | 0.00039316 | 0.90824829 | 3 | 0.001798852 | -0.00270681 |
| gradient_firstorder_Uniformity | -0.00045398 | 0.00167957 | 0.65713484 | 3 | 0.009170117 | -0.01007807 |
| square_glrlm_RunLengthNonUniformityNormalized | -0.00045398 | 0.00141755 | 0.68257419 | 3 | 0.007668717 | -0.00857667 |
| gradient_glcm_JointEntropy | -0.00045398 | 0.00167957 | 0.65713484 | 3 | 0.009170117 | -0.01007807 |
| gradient_glcm_JointEnergy | -0.00045398 | 0.00167957 | 0.65713484 | 3 | 0.009170117 | -0.01007807 |
| square_firstorder_Energy | -0.00045398 | 0.00104019 | 0.73570226 | 3 | 0.00550645 | -0.00641441 |
| wavelet-HL_glszm_ZoneVariance | -0.00045398 | 0.00039316 | 0.90824829 | 3 | 0.001798852 | -0.00270681 |
| wavelet-HL_glrlm_ShortRunHighGrayLevelEmphasis | -0.00045398 | 0.00297477 | 0.59186304 | 3 | 0.016591775 | -0.01749973 |
| logarithm_glszm_LargeAreaEmphasis | -0.00045398 | 0.00347782 | 0.5789337 | 3 | 0.019474339 | -0.0203823 |
| squareroot_glrlm_RunVariance | -0.00045398 | 0.00167957 | 0.65713484 | 3 | 0.009170117 | -0.01007807 |
| logarithm_glcm_Contrast | -0.00045398 | 0.00078631 | 0.78867514 | 3 | 0.004051682 | -0.00495964 |
| logarithm_ngtdm_Complexity | -0.00045398 | 0.00070877 | 0.8086067 | 3 | 0.003607369 | -0.00451533 |
| wavelet-LH_glszm_SmallAreaLowGrayLevelEmphasis | -0.00045398 | 0.0010945 | 0.72645541 | 3 | 0.005817636 | -0.00672559 |
| logarithm_glcm_DifferenceVariance | -0.00045398 | 0.00078631 | 0.78867514 | 3 | 0.004051682 | -0.00495964 |
| wavelet-LL_glszm_GrayLevelNonUniformity | -0.00045398 | 0.00403345 | 0.56827887 | 3 | 0.022658127 | -0.02356608 |
| wavelet-LH_glrlm_LowGrayLevelRunEmphasis | -0.00045398 | 0.00098289 | 0.74618298 | 3 | 0.005178097 | -0.00608605 |
| wavelet-HL_gldm_GrayLevelVariance | -0.00045398 | 0.00153533 | 0.67025131 | 3 | 0.008343605 | -0.00925156 |
| wavelet-HH_glszm_ZoneEntropy | -0.00045398 | 0.00335913 | 0.58164966 | 3 | 0.018794211 | -0.01970217 |
| square_glcm_Imc1 | -0.00045398 | 0.0010945 | 0.72645541 | 3 | 0.005817636 | -0.00672559 |
| wavelet-LL_glcm_Correlation | -0.00045398 | 0.00104019 | 0.73570226 | 3 | 0.00550645 | -0.00641441 |
| logarithm_firstorder_Entropy | -0.00045398 | 0.00444371 | 0.56207709 | 3 | 0.025008983 | -0.02591694 |
| squareroot_gldm_DependenceVariance | -0.00056747 | 0.00085687 | 0.81497039 | 3 | 0.004342457 | -0.0054774 |
| square_firstorder_90Percentile | -0.00056747 | 0.00070877 | 0.85007002 | 3 | 0.003493875 | -0.00462882 |
| wavelet-HH_gldm_LowGrayLevelEmphasis | -0.00056747 | 0.0005201 | 0.90032039 | 3 | 0.002412742 | -0.00354769 |
| wavelet-HH_glrlm_LongRunEmphasis | -0.00056747 | 0.00085687 | 0.81497039 | 3 | 0.004342457 | -0.0054774 |
| square_glcm_Imc2 | -0.00056747 | 0.00137605 | 0.72541741 | 3 | 0.007317433 | -0.00845238 |
| squareroot_glcm_Id | -0.00056747 | 0.00160906 | 0.6982629 | 3 | 0.008652632 | -0.00978758 |
| wavelet-LL_glcm_DifferenceEntropy | -0.00056747 | 0.00160906 | 0.6982629 | 3 | 0.008652632 | -0.00978758 |
| original_gldm_DependenceVariance | -0.00056747 | 0.00382697 | 0.58934271 | 3 | 0.021361489 | -0.02249643 |
| square_glszm_ZoneEntropy | -0.00056747 | 0.00160906 | 0.6982629 | 3 | 0.008652632 | -0.00978758 |
| square_ngtdm_Complexity | -0.00056747 | 0.00098289 | 0.78867514 | 3 | 0.005064603 | -0.00619955 |
| wavelet-HH_firstorder_Skewness | -0.00056747 | 0.00208039 | 0.65843106 | 3 | 0.011353384 | -0.01248833 |
| wavelet-HH_glrlm_ShortRunHighGrayLevelEmphasis | -0.00056747 | 0.00137605 | 0.72541741 | 3 | 0.007317433 | -0.00845238 |
| wavelet-HL_glcm_Correlation | -0.00056747 | 0.00128905 | 0.7372895 | 3 | 0.006818925 | -0.00795387 |
| square_glcm_JointAverage | -0.00056747 | 0.00098289 | 0.78867514 | 3 | 0.005064603 | -0.00619955 |
| logarithm_glszm_GrayLevelNonUniformityNormalized | -0.00056747 | 0.00098289 | 0.78867514 | 3 | 0.005064603 | -0.00619955 |
| wavelet-LL_gldm_LargeDependenceLowGrayLevelEmphasis | -0.00056747 | 0.00085687 | 0.81497039 | 3 | 0.004342457 | -0.0054774 |
| gradient_firstorder_Energy | -0.00056747 | 0.00098289 | 0.78867514 | 3 | 0.005064603 | -0.00619955 |
| wavelet-LL_firstorder_Skewness | -0.00056747 | 0.00193607 | 0.66893435 | 3 | 0.010526429 | -0.01166137 |
| logarithm_glrlm_ShortRunEmphasis | -0.00056747 | 0.00196578 | 0.66666667 | 3 | 0.010696678 | -0.01183162 |
| wavelet-LH_firstorder_Median | -0.00056747 | 0.00231762 | 0.64362125 | 3 | 0.012712765 | -0.01384771 |
| square_glszm_SizeZoneNonUniformity | -0.00056747 | 0.00193607 | 0.66893435 | 3 | 0.010526429 | -0.01166137 |
| gradient_firstorder_Mean | -0.00068097 | 0.00034048 | 0.96291005 | 3 | 0.001270041 | -0.00263198 |
| original_glcm_InverseVariance | -0.00068097 | 0.00442629 | 0.59258201 | 3 | 0.024682139 | -0.02604407 |
| original_firstorder_RootMeanSquared | -0.00068097 | 0.00117947 | 0.78867514 | 3 | 0.006077523 | -0.00743946 |
| gradient_firstorder_Entropy | -0.00068097 | 0.00148413 | 0.74494897 | 3 | 0.00782328 | -0.00918521 |
| logarithm_glszm_LargeAreaHighGrayLevelEmphasis | -0.00068097 | 0.00058974 | 0.90824829 | 3 | 0.002698278 | -0.00406021 |
| gradient_firstorder_InterquartileRange | -0.00068097 | 0.00245526 | 0.66081688 | 3 | 0.013387953 | -0.01474989 |
| original_gldm_LargeDependenceEmphasis | -0.00068097 | 0.00117947 | 0.78867514 | 3 | 0.006077523 | -0.00743946 |
| wavelet-LL_glszm_SmallAreaLowGrayLevelEmphasis | -0.00068097 | 0.00170242 | 0.71997067 | 3 | 0.009074074 | -0.01043601 |
| square_firstorder_Range | -0.00068097 | 0.00238338 | 0.66514457 | 3 | 0.01297609 | -0.01433802 |
| gradient_gldm_DependenceVariance | -0.00068097 | 0.00034048 | 0.96291005 | 3 | 0.001270041 | -0.00263198 |
| logarithm_glcm_DifferenceAverage | -0.00068097 | 0.00090084 | 0.83968311 | 3 | 0.004480915 | -0.00584285 |
| wavelet-LL_firstorder_RootMeanSquared | -0.00068097 | 0.00122763 | 0.78097574 | 3 | 0.006353493 | -0.00771543 |
| wavelet-HH_glcm_SumSquares | -0.00068097 | 0.00148413 | 0.74494897 | 3 | 0.00782328 | -0.00918521 |
| wavelet-HH_firstorder_RootMeanSquared | -0.00068097 | 0.00034048 | 0.96291005 | 3 | 0.001270041 | -0.00263198 |
| original_firstorder_Skewness | -0.00068097 | 0.00212632 | 0.68257419 | 3 | 0.011503075 | -0.01286501 |
| exponential_gldm_LargeDependenceEmphasis | -0.00068097 | 0.00122763 | 0.78097574 | 3 | 0.006353493 | -0.00771543 |
| exponential_gldm_LargeDependenceLowGrayLevelEmphasis | -0.00068097 | 0.00122763 | 0.78097574 | 3 | 0.006353493 | -0.00771543 |
| exponential_glrlm_RunPercentage | -0.00068097 | 0.00122763 | 0.78097574 | 3 | 0.006353493 | -0.00771543 |
| squareroot_glrlm_LowGrayLevelRunEmphasis | -0.00079446 | 0.00141755 | 0.78295823 | 3 | 0.007328233 | -0.00891716 |
| wavelet-LL_glrlm_RunPercentage | -0.00079446 | 0.0025781 | 0.67655165 | 3 | 0.013978334 | -0.01556726 |
| wavelet-HH_glrlm_LongRunLowGrayLevelEmphasis | -0.00079446 | 0.00196578 | 0.72180349 | 3 | 0.010469689 | -0.01205861 |
| original_ngtdm_Complexity | -0.00079446 | 0.0025781 | 0.67655165 | 3 | 0.013978334 | -0.01556726 |
| logarithm_glszm_SizeZoneNonUniformity | -0.00079446 | 0.00070877 | 0.90414519 | 3 | 0.003266886 | -0.00485581 |
| logarithm_ngtdm_Coarseness | -0.00079446 | 0.00119574 | 0.81558437 | 3 | 0.006057254 | -0.00764618 |
| wavelet-HH_glcm_Imc2 | -0.00079446 | 0.00171373 | 0.74687097 | 3 | 0.009025397 | -0.01061432 |
| wavelet-HH_gldm_LargeDependenceLowGrayLevelEmphasis | -0.00079446 | 0.00196578 | 0.72180349 | 3 | 0.010469689 | -0.01205861 |
| original_glrlm_GrayLevelNonUniformity | -0.00079446 | 0.00070877 | 0.90414519 | 3 | 0.003266886 | -0.00485581 |
| squareroot_ngtdm_Contrast | -0.00079446 | 0.00098289 | 0.85176324 | 3 | 0.004837614 | -0.00642654 |
| logarithm_glrlm_RunVariance | -0.00079446 | 0.00137605 | 0.78867514 | 3 | 0.007090444 | -0.00867937 |
| squareroot_glszm_SizeZoneNonUniformityNormalized | -0.00079446 | 0.00341051 | 0.63717582 | 3 | 0.01874811 | -0.02033703 |
| squareroot_gldm_SmallDependenceHighGrayLevelEmphasis | -0.00079446 | 0.00273095 | 0.66781216 | 3 | 0.014854197 | -0.01644312 |
| logarithm_glcm_JointEnergy | -0.00079446 | 0.00250974 | 0.68073922 | 3 | 0.013586643 | -0.01517557 |
| squareroot_gldm_DependenceEntropy | -0.00090796 | 0.00226705 | 0.72019275 | 3 | 0.012082496 | -0.01389841 |
| square_gldm_DependenceNonUniformityNormalized | -0.00090796 | 0.002189 | 0.72645541 | 3 | 0.011635271 | -0.01345118 |
| wavelet-LH_glcm_Correlation | -0.00090796 | 0.00397555 | 0.63468701 | 3 | 0.021872379 | -0.02368829 |
| square_glszm_GrayLevelVariance | -0.00090796 | 0.00160906 | 0.78426762 | 3 | 0.008312149 | -0.01012806 |
| wavelet-HH_firstorder_Range | -0.00090796 | 0.00449559 | 0.62006005 | 3 | 0.024852248 | -0.02666816 |
| logarithm_gldm_SmallDependenceHighGrayLevelEmphasis | -0.00090796 | 0.00019658 | 0.99236596 | 3 | 0.000218459 | -0.00203437 |
| original_glrlm_RunVariance | -0.00090796 | 0.0045341 | 0.61909827 | 3 | 0.025072949 | -0.02688886 |
| wavelet-HL_glcm_SumEntropy | -0.00090796 | 0.002189 | 0.72645541 | 3 | 0.011635271 | -0.01345118 |
| gradient_glszm_GrayLevelNonUniformityNormalized | -0.00090796 | 0.00221532 | 0.72430886 | 3 | 0.011786097 | -0.01360201 |
| wavelet-HL_gldm_LowGrayLevelEmphasis | -0.00090796 | 0.00344433 | 0.65361908 | 3 | 0.018828431 | -0.02064434 |
| square_gldm_SmallDependenceLowGrayLevelEmphasis | -0.00090796 | 0.0010945 | 0.85634832 | 3 | 0.005363658 | -0.00717957 |
| wavelet-HH_glrlm_RunPercentage | -0.00090796 | 0.00174723 | 0.76846242 | 3 | 0.00910384 | -0.01091975 |
| wavelet-HH_firstorder_Variance | -0.00090796 | 0.00137605 | 0.81426968 | 3 | 0.00697695 | -0.00879286 |
| wavelet-HH_glcm_DifferenceVariance | -0.00090796 | 0.00137605 | 0.81426968 | 3 | 0.00697695 | -0.00879286 |
| wavelet-HH_glcm_ClusterTendency | -0.00090796 | 0.00137605 | 0.81426968 | 3 | 0.00697695 | -0.00879286 |
| logarithm_glrlm_GrayLevelVariance | -0.00090796 | 0.00137605 | 0.81426968 | 3 | 0.00697695 | -0.00879286 |
| wavelet-HH_glcm_Contrast | -0.00090796 | 0.00137605 | 0.81426968 | 3 | 0.00697695 | -0.00879286 |
| exponential_firstorder_InterquartileRange | -0.00102145 | 0.00353841 | 0.66666667 | 3 | 0.019254021 | -0.02129692 |
| logarithm_glcm_Idn | -0.00102145 | 0.00238338 | 0.732379 | 3 | 0.012635606 | -0.01467851 |
| wavelet-HL_glcm_Idn | -0.00102145 | 0.00122763 | 0.85687321 | 3 | 0.006013009 | -0.00805591 |
| gradient_glcm_DifferenceAverage | -0.00102145 | 0.00207108 | 0.75851825 | 3 | 0.010846069 | -0.01288897 |
| wavelet-HH_glrlm_LowGrayLevelRunEmphasis | -0.00102145 | 0.0017692 | 0.78867514 | 3 | 0.009116285 | -0.01115919 |
| wavelet-HL_glrlm_GrayLevelNonUniformityNormalized | -0.00102145 | 0.00207108 | 0.75851825 | 3 | 0.010846069 | -0.01288897 |
| original_glcm_MCC | -0.00102145 | 0.0044524 | 0.63525045 | 3 | 0.024491291 | -0.02653419 |
| wavelet-HL_firstorder_RootMeanSquared | -0.00102145 | 0.00090084 | 0.90575134 | 3 | 0.004140432 | -0.00618333 |
| wavelet-HH_glcm_DifferenceAverage | -0.00102145 | 0.00383705 | 0.65498823 | 3 | 0.020965295 | -0.0230082 |
| wavelet-LL_firstorder_RobustMeanAbsoluteDeviation | -0.00102145 | 0 | 0.5 | 3 | -0.00102145 | -0.00102145 |
| square_glcm_DifferenceVariance | -0.00102145 | 0.0017692 | 0.78867514 | 3 | 0.009116285 | -0.01115919 |
| wavelet-LL_firstorder_InterquartileRange | -0.00102145 | 0 | 0.5 | 3 | -0.00102145 | -0.00102145 |
| wavelet-HL_gldm_DependenceVariance | -0.00102145 | 0.00245526 | 0.72699497 | 3 | 0.013047469 | -0.01509037 |
| air space | -0.00102145 | 0.00068097 | 0.93915503 | 3 | 0.002880566 | -0.00492347 |
| original_firstorder_RobustMeanAbsoluteDeviation | -0.00102145 | 0 | 0.5 | 3 | -0.00102145 | -0.00102145 |
| wavelet-LH_firstorder_Energy | -0.00102145 | 0 | 0.5 | 3 | -0.00102145 | -0.00102145 |
| wavelet-LH_glrlm_RunLengthNonUniformity | -0.00102145 | 0.00117947 | 0.86380344 | 3 | 0.00573704 | -0.00777994 |
| logarithm_glcm_Id | -0.00102145 | 0.00180167 | 0.78517591 | 3 | 0.009302314 | -0.01134522 |
| squareroot_glrlm_RunLengthNonUniformityNormalized | -0.00113495 | 0.00226705 | 0.76135419 | 3 | 0.011855507 | -0.0141254 |
| squareroot_gldm_LargeDependenceEmphasis | -0.00113495 | 0.002189 | 0.76802813 | 3 | 0.011408282 | -0.01367817 |
| squareroot_firstorder_Median | -0.00113495 | 0.00019658 | 0.99507377 | 3 | -8.53E-06 | -0.00226136 |
| gradient_firstorder_RootMeanSquared | -0.00113495 | 0.00196578 | 0.78867514 | 3 | 0.010129206 | -0.0123991 |
| wavelet-LL_glszm_ZoneEntropy | -0.00113495 | 0.00205234 | 0.78038608 | 3 | 0.010625174 | -0.01289506 |
| squareroot_glcm_DifferenceAverage | -0.00113495 | 0.00196578 | 0.78867514 | 3 | 0.010129206 | -0.0123991 |
| wavelet-HL_firstorder_Maximum | -0.00113495 | 0.00297477 | 0.71166688 | 3 | 0.015910808 | -0.0181807 |
| wavelet-HL_gldm_SmallDependenceLowGrayLevelEmphasis | -0.00113495 | 0.00019658 | 0.99507377 | 3 | -8.53E-06 | -0.00226136 |
| wavelet-HL_glszm_SmallAreaHighGrayLevelEmphasis | -0.00113495 | 0.0039901 | 0.66448792 | 3 | 0.021728784 | -0.02399867 |
| wavelet-HH_glcm_JointEntropy | -0.00113495 | 0.00160906 | 0.82686023 | 3 | 0.00808516 | -0.01035505 |
| logarithm_firstorder_Variance | -0.00113495 | 0.00098289 | 0.90824829 | 3 | 0.00449713 | -0.00676702 |
| logarithm_gldm_GrayLevelVariance | -0.00113495 | 0.00098289 | 0.90824829 | 3 | 0.00449713 | -0.00676702 |
| original_glszm_GrayLevelNonUniformityNormalized | -0.00113495 | 0.00019658 | 0.99507377 | 3 | -8.53E-06 | -0.00226136 |
| gradient_glszm_SizeZoneNonUniformity | -0.00113495 | 0.00462272 | 0.64397807 | 3 | 0.025353778 | -0.02762367 |
| wavelet-LL_glrlm_LowGrayLevelRunEmphasis | -0.00113495 | 0.00119574 | 0.87904902 | 3 | 0.00571677 | -0.00798666 |
| wavelet-LH_glcm_Autocorrelation | -0.00124844 | 0.0010945 | 0.9065717 | 3 | 0.005023174 | -0.00752005 |
| wavelet-LH_glszm_SmallAreaEmphasis | -0.00124844 | 0.00439123 | 0.66441735 | 3 | 0.023913767 | -0.02641065 |
| square_glszm_SmallAreaEmphasis | -0.00124844 | 0.00728136 | 0.60275439 | 3 | 0.040474559 | -0.04297144 |
| squareroot_glcm_DifferenceEntropy | -0.00124844 | 0.00216236 | 0.78867514 | 3 | 0.011142126 | -0.01363901 |
| squareroot_glrlm_RunPercentage | -0.00124844 | 0.00216236 | 0.78867514 | 3 | 0.011142126 | -0.01363901 |
| squareroot_glrlm_ShortRunEmphasis | -0.00124844 | 0.00216236 | 0.78867514 | 3 | 0.011142126 | -0.01363901 |
| squareroot_firstorder_90Percentile | -0.00124844 | 0.00160906 | 0.84442336 | 3 | 0.007971665 | -0.01046854 |
| gradient_glcm_DifferenceEntropy | -0.00124844 | 0.0010945 | 0.9065717 | 3 | 0.005023174 | -0.00752005 |
| wavelet-LH_gldm_LargeDependenceLowGrayLevelEmphasis | -0.00124844 | 0.0010945 | 0.9065717 | 3 | 0.005023174 | -0.00752005 |
| original_glszm_ZoneEntropy | -0.00124844 | 0.00273095 | 0.74426358 | 3 | 0.014400219 | -0.0168971 |
| gradient_firstorder_Range | -0.00124844 | 0.00316363 | 0.71757664 | 3 | 0.016879496 | -0.01937637 |
| original_firstorder_10Percentile | -0.00124844 | 0.00039316 | 0.984248 | 3 | 0.001004391 | -0.00350127 |
| wavelet-HH_glrlm_GrayLevelNonUniformity | -0.00124844 | 0.00019658 | 0.9959183 | 3 | -0.00012202 | -0.00237486 |
| logarithm_glszm_HighGrayLevelZoneEmphasis | -0.00136193 | 0.00207108 | 0.81362502 | 3 | 0.010505585 | -0.01322945 |
| gradient_gldm_HighGrayLevelEmphasis | -0.00136193 | 0.00180167 | 0.83968311 | 3 | 0.008961831 | -0.0116857 |
| logarithm_firstorder_Kurtosis | -0.00136193 | 0.00117947 | 0.90824829 | 3 | 0.005396556 | -0.00812042 |
| wavelet-LL_firstorder_Median | -0.00136193 | 0.00170242 | 0.84992711 | 3 | 0.008393107 | -0.01111698 |
| exponential_glrlm_RunLengthNonUniformityNormalized | -0.00136193 | 0.00245526 | 0.78097574 | 3 | 0.012706986 | -0.01543085 |
| wavelet-HH_gldm_SmallDependenceLowGrayLevelEmphasis | -0.00136193 | 0.00238338 | 0.78669109 | 3 | 0.012295123 | -0.01501899 |
| logarithm_glrlm_LongRunEmphasis | -0.00136193 | 0.00235894 | 0.78867514 | 3 | 0.012155047 | -0.01487892 |
| square_glrlm_RunVariance | -0.00136193 | 0.00122763 | 0.90269363 | 3 | 0.005672526 | -0.00839639 |
| wavelet-HH_gldm_DependenceEntropy | -0.00136193 | 0.00034048 | 0.98989795 | 3 | 0.000589074 | -0.00331294 |
| gradient_glcm_Contrast | -0.00136193 | 0.00034048 | 0.98989795 | 3 | 0.000589074 | -0.00331294 |
| logarithm_gldm_LargeDependenceEmphasis | -0.00136193 | 0.00335337 | 0.72268089 | 3 | 0.017853268 | -0.02057714 |
| squareroot_glrlm_GrayLevelNonUniformity | -0.00136193 | 0.00058974 | 0.97140452 | 3 | 0.002017311 | -0.00474118 |
| logarithm_glcm_MaximumProbability | -0.00136193 | 0.00290909 | 0.748708 | 3 | 0.015307487 | -0.01803136 |
| wavelet-LH_glrlm_GrayLevelNonUniformityNormalized | -0.00147543 | 0.00019658 | 0.99706742 | 3 | -0.00034901 | -0.00260184 |
| squareroot_firstorder_InterquartileRange | -0.00147543 | 0.00373499 | 0.71775819 | 3 | 0.019926458 | -0.02287732 |
| logarithm_glrlm_LongRunHighGrayLevelEmphasis | -0.00147543 | 0.00255552 | 0.78867514 | 3 | 0.013167967 | -0.01611882 |
| wavelet-LL_ngtdm_Complexity | -0.00147543 | 0.00255552 | 0.78867514 | 3 | 0.013167967 | -0.01611882 |
| wavelet-LL_firstorder_Mean | -0.00147543 | 0.00137605 | 0.89779354 | 3 | 0.006409477 | -0.00936033 |
| logarithm_glszm_SmallAreaEmphasis | -0.00147543 | 0.00171373 | 0.86279462 | 3 | 0.00834443 | -0.01129529 |
| square_firstorder_Maximum | -0.00147543 | 0.00255552 | 0.78867514 | 3 | 0.013167967 | -0.01611882 |
| original_firstorder_Mean | -0.00147543 | 0.00137605 | 0.89779354 | 3 | 0.006409477 | -0.00936033 |
| wavelet-LH_glrlm_ShortRunEmphasis | -0.00147543 | 0.00070877 | 0.96547467 | 3 | 0.002585919 | -0.00553678 |
| gradient_glrlm_RunLengthNonUniformityNormalized | -0.00147543 | 0.00285546 | 0.76737427 | 3 | 0.014886696 | -0.01783755 |
| original_ngtdm_Contrast | -0.00147543 | 0.00019658 | 0.99706742 | 3 | -0.00034901 | -0.00260184 |
| square_gldm_GrayLevelNonUniformity | -0.00147543 | 0.00039316 | 0.98856992 | 3 | 0.000777402 | -0.00372826 |
| logarithm_glrlm_RunLengthNonUniformity | -0.00147543 | 0.00039316 | 0.98856992 | 3 | 0.000777402 | -0.00372826 |
| original_gldm_GrayLevelNonUniformity | -0.00147543 | 0.00039316 | 0.98856992 | 3 | 0.000777402 | -0.00372826 |
| wavelet-HH_glszm_ZoneVariance | -0.00147543 | 0.00171373 | 0.86279462 | 3 | 0.00834443 | -0.01129529 |
| square_glrlm_RunEntropy | -0.00147543 | 0.00104019 | 0.93333333 | 3 | 0.004485 | -0.00743586 |
| wavelet-LL_ngtdm_Contrast | -0.00147543 | 0.00019658 | 0.99706742 | 3 | -0.00034901 | -0.00260184 |
| wavelet-HH_gldm_DependenceNonUniformityNormalized | -0.00147543 | 0.00409053 | 0.70204271 | 3 | 0.021963751 | -0.02491461 |
| wavelet-HH_glrlm_RunLengthNonUniformity | -0.00147543 | 0.00039316 | 0.98856992 | 3 | 0.000777402 | -0.00372826 |
| wavelet-HH_glcm_MaximumProbability | -0.00158892 | 0.00264469 | 0.79633363 | 3 | 0.013565442 | -0.01674329 |
| logarithm_glcm_JointAverage | -0.00158892 | 0.00160906 | 0.88533732 | 3 | 0.007631182 | -0.01080903 |
| gradient_glrlm_GrayLevelNonUniformityNormalized | -0.00158892 | 0.0027521 | 0.78867514 | 3 | 0.014180888 | -0.01735873 |
| wavelet-HH_glrlm_ShortRunLowGrayLevelEmphasis | -0.00158892 | 0.00255552 | 0.80291963 | 3 | 0.013054473 | -0.01623232 |
| squareroot_gldm_DependenceNonUniformity | -0.00158892 | 0.00019658 | 0.99746834 | 3 | -0.00046251 | -0.00271534 |
| wavelet-LL_firstorder_90Percentile | -0.00158892 | 0.00137605 | 0.90824829 | 3 | 0.006295983 | -0.00947383 |
| wavelet-LL_glszm_LargeAreaLowGrayLevelEmphasis | -0.00158892 | 0.00160906 | 0.88533732 | 3 | 0.007631182 | -0.01080903 |
| wavelet-LH_gldm_GrayLevelNonUniformity | -0.00158892 | 0.0005201 | 0.98304589 | 3 | 0.001391291 | -0.00456914 |
| logarithm_ngtdm_Contrast | -0.00158892 | 0.00078631 | 0.96358633 | 3 | 0.002916737 | -0.00609458 |
| logarithm_glcm_JointEntropy | -0.00158892 | 0.00308947 | 0.76648545 | 3 | 0.01611408 | -0.01929193 |
| logarithm_gldm_SmallDependenceEmphasis | -0.00170242 | 0.00170242 | 0.88729834 | 3 | 0.008052623 | -0.01145746 |
| original_glcm_Idmn | -0.00170242 | 0.00034048 | 0.99346377 | 3 | 0.000248591 | -0.00365343 |
| logarithm_glrlm_LongRunLowGrayLevelEmphasis | -0.00170242 | 0.00090084 | 0.95899255 | 3 | 0.003459465 | -0.0068643 |
| wavelet-LL_glszm_GrayLevelVariance | -0.00170242 | 0.00414216 | 0.72480899 | 3 | 0.022032621 | -0.02543746 |
| wavelet-LL_glcm_Idmn | -0.00170242 | 0.00034048 | 0.99346377 | 3 | 0.000248591 | -0.00365343 |
| logarithm_glszm_ZoneVariance | -0.00170242 | 0.00360334 | 0.75041771 | 3 | 0.018945112 | -0.02234995 |
| wavelet-LH_gldm_DependenceNonUniformity | -0.00170242 | 0 | 0.5 | 3 | -0.00170242 | -0.00170242 |
| exponential_glszm_LargeAreaHighGrayLevelEmphasis | -0.00170242 | 0 | 0.5 | 3 | -0.00170242 | -0.00170242 |
| wavelet-HH_gldm_GrayLevelNonUniformity | -0.00170242 | 0 | 0.5 | 3 | -0.00170242 | -0.00170242 |
| wavelet-LH_glrlm_ShortRunHighGrayLevelEmphasis | -0.00170242 | 0 | 0.5 | 3 | -0.00170242 | -0.00170242 |
| gradient_glcm_Idmn | -0.00170242 | 0 | 0.5 | 3 | -0.00170242 | -0.00170242 |
| exponential_glrlm_LongRunEmphasis | -0.00170242 | 0 | 0.5 | 3 | -0.00170242 | -0.00170242 |
| gradient_glszm_ZonePercentage | -0.00170242 | 0 | 0.5 | 3 | -0.00170242 | -0.00170242 |
| squareroot_firstorder_MeanAbsoluteDeviation | -0.00170242 | 0.00170242 | 0.88729834 | 3 | 0.008052623 | -0.01145746 |
| exponential_glrlm_LongRunLowGrayLevelEmphasis | -0.00170242 | 0 | 0.5 | 3 | -0.00170242 | -0.00170242 |
| wavelet-LH_glrlm_HighGrayLevelRunEmphasis | -0.00170242 | 0 | 0.5 | 3 | -0.00170242 | -0.00170242 |
| wavelet-HH_gldm_DependenceNonUniformity | -0.00170242 | 0 | 0.5 | 3 | -0.00170242 | -0.00170242 |
| gradient_gldm_SmallDependenceEmphasis | -0.00170242 | 0 | 0.5 | 3 | -0.00170242 | -0.00170242 |
| exponential_glszm_LargeAreaLowGrayLevelEmphasis | -0.00170242 | 0 | 0.5 | 3 | -0.00170242 | -0.00170242 |
| logarithm_firstorder_Maximum | -0.00170242 | 0.00238338 | 0.82921346 | 3 | 0.01195464 | -0.01535947 |
| wavelet-LH_gldm_LowGrayLevelEmphasis | -0.00170242 | 0 | 0.5 | 3 | -0.00170242 | -0.00170242 |
| exponential_gldm_DependenceNonUniformity | -0.00170242 | 0 | 0.5 | 3 | -0.00170242 | -0.00170242 |
| exponential_glszm_LargeAreaEmphasis | -0.00170242 | 0 | 0.5 | 3 | -0.00170242 | -0.00170242 |
| wavelet-HH_glszm_SmallAreaLowGrayLevelEmphasis | -0.00181591 | 0.00307065 | 0.79329423 | 3 | 0.015779254 | -0.01941108 |
| wavelet-LH_glcm_JointEnergy | -0.00181591 | 0.00160906 | 0.90509575 | 3 | 0.007404193 | -0.01103602 |
| logarithm_glcm_SumSquares | -0.00181591 | 0.00019658 | 0.99805825 | 3 | -0.0006895 | -0.00294233 |
| wavelet-LH_glrlm_LongRunLowGrayLevelEmphasis | -0.00181591 | 0.00085687 | 0.96656948 | 3 | 0.003094018 | -0.00672584 |
| square_gldm_DependenceNonUniformity | -0.00181591 | 0.00019658 | 0.99805825 | 3 | -0.0006895 | -0.00294233 |
| logarithm_glrlm_GrayLevelNonUniformity | -0.00181591 | 0.00019658 | 0.99805825 | 3 | -0.0006895 | -0.00294233 |
| wavelet-LL_glrlm_RunLengthNonUniformity | -0.00181591 | 0.00239148 | 0.84050261 | 3 | 0.011887519 | -0.01551934 |
| square_glszm_ZoneVariance | -0.00181591 | 0.00019658 | 0.99805825 | 3 | -0.0006895 | -0.00294233 |
| wavelet-LL_glrlm_GrayLevelNonUniformity | -0.00181591 | 0.00019658 | 0.99805825 | 3 | -0.0006895 | -0.00294233 |
| wavelet-HL_glszm_ZoneEntropy | -0.00181591 | 0.0043113 | 0.7292273 | 3 | 0.022888299 | -0.02652012 |
| squareroot_glszm_GrayLevelNonUniformity | -0.00181591 | 0.00019658 | 0.99805825 | 3 | -0.0006895 | -0.00294233 |
| wavelet-LH_glrlm_GrayLevelNonUniformity | -0.00181591 | 0.00019658 | 0.99805825 | 3 | -0.0006895 | -0.00294233 |
| logarithm_glszm_GrayLevelNonUniformity | -0.00181591 | 0.00019658 | 0.99805825 | 3 | -0.0006895 | -0.00294233 |
| gradient_glszm_LargeAreaEmphasis | -0.00181591 | 0.00019658 | 0.99805825 | 3 | -0.0006895 | -0.00294233 |
| gradient_gldm_GrayLevelNonUniformity | -0.00181591 | 0.00019658 | 0.99805825 | 3 | -0.0006895 | -0.00294233 |
| logarithm_firstorder_Skewness | -0.00181591 | 0.00167957 | 0.89900373 | 3 | 0.007808183 | -0.01144001 |
| gradient_glszm_LargeAreaHighGrayLevelEmphasis | -0.00181591 | 0.00019658 | 0.99805825 | 3 | -0.0006895 | -0.00294233 |
| wavelet-HH_glszm_LargeAreaEmphasis | -0.00181591 | 0.00019658 | 0.99805825 | 3 | -0.0006895 | -0.00294233 |
| wavelet-HL_gldm_GrayLevelNonUniformity | -0.00181591 | 0.00019658 | 0.99805825 | 3 | -0.0006895 | -0.00294233 |
| gradient_glszm_LargeAreaLowGrayLevelEmphasis | -0.00181591 | 0.00019658 | 0.99805825 | 3 | -0.0006895 | -0.00294233 |
| original_glcm_Contrast | -0.00192941 | 0.00260049 | 0.8362548 | 3 | 0.012971664 | -0.01683048 |
| wavelet-HH_gldm_DependenceVariance | -0.00192941 | 0.00085687 | 0.97005107 | 3 | 0.002980523 | -0.00683934 |
| squareroot_glcm_JointEnergy | -0.00192941 | 0.00104019 | 0.95762462 | 3 | 0.004031022 | -0.00788984 |
| wavelet-LH_glszm_SizeZoneNonUniformityNormalized | -0.00192941 | 0.00791456 | 0.64304414 | 3 | 0.043421917 | -0.04728073 |
| logarithm_firstorder_RootMeanSquared | -0.00192941 | 0.00171373 | 0.90476191 | 3 | 0.007890453 | -0.01174927 |
| logarithm_firstorder_Uniformity | -0.00192941 | 0.00316363 | 0.79921417 | 3 | 0.016198529 | -0.02005734 |
| wavelet-LL_glrlm_LongRunHighGrayLevelEmphasis | -0.00192941 | 0.0027521 | 0.82572071 | 3 | 0.013840405 | -0.01769922 |
| wavelet-HH_glcm_Correlation | -0.00192941 | 0.00193607 | 0.88676339 | 3 | 0.009164495 | -0.01302331 |
| wavelet-LH_glszm_GrayLevelNonUniformity | -0.00192941 | 0.00393157 | 0.75757576 | 3 | 0.020598895 | -0.02445771 |
| logarithm_gldm_DependenceNonUniformity | -0.00192941 | 0.00104019 | 0.95762462 | 3 | 0.004031022 | -0.00788984 |
| original_glszm_GrayLevelNonUniformity | -0.0020429 | 0.00090084 | 0.97043754 | 3 | 0.003118981 | -0.00720478 |
| wavelet-LL_glcm_DifferenceVariance | -0.0020429 | 0.0027025 | 0.83968311 | 3 | 0.013442746 | -0.01752855 |
| original_gldm_DependenceNonUniformityNormalized | -0.0020429 | 0.00683516 | 0.67187345 | 3 | 0.037123314 | -0.04120912 |
| logarithm_gldm_DependenceVariance | -0.0020429 | 0.00180167 | 0.90575134 | 3 | 0.008280864 | -0.01236667 |
| squareroot_firstorder_Skewness | -0.0020429 | 0.00148413 | 0.93003663 | 3 | 0.006461346 | -0.01054715 |
| wavelet-HL_glrlm_LowGrayLevelRunEmphasis | -0.0020429 | 0.00383705 | 0.77310371 | 3 | 0.019943844 | -0.02402965 |
| logarithm_glrlm_HighGrayLevelRunEmphasis | -0.0020429 | 0.0017692 | 0.90824829 | 3 | 0.008094835 | -0.01218064 |
| logarithm_gldm_HighGrayLevelEmphasis | -0.0020429 | 0.0017692 | 0.90824829 | 3 | 0.008094835 | -0.01218064 |
| logarithm_glrlm_ShortRunHighGrayLevelEmphasis | -0.0020429 | 0.0017692 | 0.90824829 | 3 | 0.008094835 | -0.01218064 |
| logarithm_glcm_Autocorrelation | -0.0020429 | 0.0017692 | 0.90824829 | 3 | 0.008094835 | -0.01218064 |
| squareroot_glrlm_GrayLevelNonUniformityNormalized | -0.0020429 | 0.00257059 | 0.84874292 | 3 | 0.012686887 | -0.01677269 |
| wavelet-LL_firstorder_Energy | -0.0020429 | 0.00102145 | 0.96291005 | 3 | 0.003810123 | -0.00789593 |
| logarithm_glszm_SmallAreaHighGrayLevelEmphasis | -0.0020429 | 0.0017692 | 0.90824829 | 3 | 0.008094835 | -0.01218064 |
| squareroot_glcm_Idm | -0.0021564 | 0.00137605 | 0.94342203 | 3 | 0.00572851 | -0.0100413 |
| exponential_firstorder_Mean | -0.0021564 | 0.00373499 | 0.78867514 | 3 | 0.019245491 | -0.02355828 |
| original_firstorder_Minimum | -0.0021564 | 0.00403345 | 0.77389922 | 3 | 0.02095571 | -0.0252685 |
| original_firstorder_Kurtosis | -0.0021564 | 0.00226705 | 0.87939346 | 3 | 0.010834057 | -0.01514685 |
| wavelet-HL_firstorder_Kurtosis | -0.0021564 | 0.00221532 | 0.88307697 | 3 | 0.010537658 | -0.01485045 |
| wavelet-HH_ngtdm_Contrast | -0.0021564 | 0.00289578 | 0.83693028 | 3 | 0.014436735 | -0.01874953 |
| logarithm_glcm_SumEntropy | -0.0021564 | 0.00553569 | 0.71529831 | 3 | 0.029563741 | -0.03387653 |
| wavelet-HH_glszm_GrayLevelVariance | -0.0021564 | 0.00171373 | 0.91943525 | 3 | 0.007663464 | -0.01197625 |
| wavelet-HL_gldm_DependenceEntropy | -0.0021564 | 0.00491446 | 0.73668778 | 3 | 0.026003981 | -0.03031677 |
| square_ngtdm_Strength | -0.00226989 | 0.00308947 | 0.83445003 | 3 | 0.015433113 | -0.01997289 |
| wavelet-LH_glszm_SmallAreaHighGrayLevelEmphasis | -0.00226989 | 0.00264469 | 0.86226178 | 3 | 0.012884475 | -0.01742426 |
| exponential_firstorder_90Percentile | -0.00226989 | 0.00241559 | 0.87742568 | 3 | 0.01157173 | -0.01611151 |
| gradient_glszm_ZoneVariance | -0.00226989 | 0.00070877 | 0.98450158 | 3 | 0.001791457 | -0.00633124 |
| wavelet-HH_glcm_SumEntropy | -0.00226989 | 0.00128905 | 0.95360921 | 3 | 0.005116508 | -0.00965629 |
| wavelet-LH_glcm_Idn | -0.00226989 | 0.00264469 | 0.86226178 | 3 | 0.012884475 | -0.01742426 |
| squareroot_firstorder_Uniformity | -0.00226989 | 0.00246312 | 0.87424059 | 3 | 0.01184405 | -0.01638383 |
| gradient_glrlm_GrayLevelVariance | -0.00226989 | 0.00264469 | 0.86226178 | 3 | 0.012884475 | -0.01742426 |
| wavelet-LH_gldm_SmallDependenceLowGrayLevelEmphasis | -0.00226989 | 0.00157263 | 0.93519414 | 3 | 0.006741431 | -0.01128121 |
| square_glrlm_GrayLevelNonUniformity | -0.00226989 | 0.00314525 | 0.83113309 | 3 | 0.015752751 | -0.02029253 |
| original_glszm_HighGrayLevelZoneEmphasis | -0.00226989 | 0.00406209 | 0.78239125 | 3 | 0.021006327 | -0.02554611 |
| exponential_firstorder_RobustMeanAbsoluteDeviation | -0.00238338 | 0.00450417 | 0.77192584 | 3 | 0.023426027 | -0.0281928 |
| wavelet-LH_glszm_ZoneEntropy | -0.00238338 | 0.00170242 | 0.93191229 | 3 | 0.007371656 | -0.01213843 |
| squareroot_firstorder_Mean | -0.00238338 | 0.00122763 | 0.96089885 | 3 | 0.004651075 | -0.00941784 |
| wavelet-HL_glrlm_HighGrayLevelRunEmphasis | -0.00238338 | 0.00494581 | 0.75413972 | 3 | 0.025956646 | -0.03072342 |
| multiple pulmonary metastases | -0.00238338 | 0.01388488 | 0.60286705 | 3 | 0.077178526 | -0.0819453 |
| squareroot_firstorder_RootMeanSquared | -0.00238338 | 0.00180167 | 0.92548147 | 3 | 0.00794038 | -0.01270715 |
| wavelet-HH_glcm_JointEnergy | -0.00249688 | 0.0010945 | 0.97075654 | 3 | 0.003774735 | -0.00876849 |
| wavelet-HH_glcm_JointAverage | -0.00249688 | 0.00449559 | 0.7812205 | 3 | 0.023263325 | -0.02825708 |
| wavelet-LH_glszm_HighGrayLevelZoneEmphasis | -0.00249688 | 0.0010945 | 0.97075654 | 3 | 0.003774735 | -0.00876849 |
| gradient_firstorder_90Percentile | -0.00261037 | 0.00349445 | 0.83750618 | 3 | 0.017413219 | -0.02263397 |
| gradient_glrlm_GrayLevelNonUniformity | -0.00261037 | 0.00349445 | 0.83750618 | 3 | 0.017413219 | -0.02263397 |
| original_glrlm_RunLengthNonUniformity | -0.00272387 | 0.00180167 | 0.93994135 | 3 | 0.007599897 | -0.01304763 |
| original_glszm_SmallAreaHighGrayLevelEmphasis | -0.00272387 | 0.00392665 | 0.82373368 | 3 | 0.019776256 | -0.02522399 |
| squareroot_glcm_DifferenceVariance | -0.00272387 | 0.00212632 | 0.92163702 | 3 | 0.009460174 | -0.01490791 |
| wavelet-HL_glrlm_GrayLevelNonUniformity | -0.00272387 | 0.00090084 | 0.98271079 | 3 | 0.002438014 | -0.00788575 |
| wavelet-HL_glcm_DifferenceEntropy | -0.00272387 | 0.00442629 | 0.80094192 | 3 | 0.022639238 | -0.02808697 |
| logarithm_firstorder_Mean | -0.00272387 | 0.0017692 | 0.9417261 | 3 | 0.007413868 | -0.0128616 |
| squareroot_firstorder_Maximum | -0.00272387 | 0.00156029 | 0.95291081 | 3 | 0.006216775 | -0.01166451 |
| original_firstorder_Median | -0.00272387 | 0.00180167 | 0.93994135 | 3 | 0.007599897 | -0.01304763 |
| wavelet-LH_firstorder_Minimum | -0.00283736 | 0.00344433 | 0.85511937 | 3 | 0.016899024 | -0.02257375 |
| wavelet-LL_firstorder_Maximum | -0.00283736 | 0.00341051 | 0.85685167 | 3 | 0.016705209 | -0.02237993 |
| squareroot_glszm_SmallAreaHighGrayLevelEmphasis | -0.00283736 | 0.00406209 | 0.82503186 | 3 | 0.020438854 | -0.02611358 |
| wavelet-HL_glcm_Contrast | -0.00283736 | 0.00264469 | 0.89787938 | 3 | 0.012317003 | -0.01799173 |
| wavelet-LH_glrlm_ShortRunLowGrayLevelEmphasis | -0.00283736 | 0.00193607 | 0.93678535 | 3 | 0.008256539 | -0.01393126 |
| wavelet-HH_firstorder_Maximum | -0.00283736 | 0.00239148 | 0.911889 | 3 | 0.010866068 | -0.01654079 |
| wavelet-HL_firstorder_Minimum | -0.00283736 | 0.0053871 | 0.77103556 | 3 | 0.028031343 | -0.03370607 |
| square_glszm_LowGrayLevelZoneEmphasis | -0.00283736 | 0.00246312 | 0.90792208 | 3 | 0.011276578 | -0.0169513 |
| wavelet-HL_glcm_JointAverage | -0.00283736 | 0.00478296 | 0.79389401 | 3 | 0.024569499 | -0.03024422 |
| original_shape2D_MaximumDiameter | -0.00283736 | 0.00559817 | 0.763699 | 3 | 0.029240756 | -0.03491548 |
| wavelet-HL_glszm_GrayLevelNonUniformityNormalized | -0.00283736 | 0.00289578 | 0.88411568 | 3 | 0.013755768 | -0.01943049 |
| wavelet-HH_gldm_LargeDependenceHighGrayLevelEmphasis | -0.00295086 | 0.00255552 | 0.90824829 | 3 | 0.011692539 | -0.01759425 |
| original_shape2D_PixelSurface | -0.00295086 | 0.00484317 | 0.79902841 | 3 | 0.024801048 | -0.03070276 |
| wavelet-HH_gldm_HighGrayLevelEmphasis | -0.00295086 | 0.00255552 | 0.90824829 | 3 | 0.011692539 | -0.01759425 |
| wavelet-LL_ngtdm_Strength | -0.00295086 | 0.00342746 | 0.86279462 | 3 | 0.016688861 | -0.02259058 |
| squareroot_glrlm_RunLengthNonUniformity | -0.00295086 | 0.00305172 | 0.88202262 | 3 | 0.014535807 | -0.02043752 |
| square_glcm_MCC | -0.00295086 | 0.00323609 | 0.87249437 | 3 | 0.015592275 | -0.02149399 |
| gradient_gldm_SmallDependenceHighGrayLevelEmphasis | -0.00295086 | 0.00216236 | 0.92906394 | 3 | 0.009439709 | -0.01534142 |
| wavelet-HL_glcm_JointEntropy | -0.00295086 | 0.00160906 | 0.95677344 | 3 | 0.006269248 | -0.01217096 |
| wavelet-HL_glrlm_ShortRunLowGrayLevelEmphasis | -0.00306435 | 0.00156029 | 0.96169026 | 3 | 0.005876291 | -0.01200499 |
| original_shape2D_Sphericity | -0.00306435 | 0.00306435 | 0.88729834 | 3 | 0.014494722 | -0.02062343 |
| logarithm_firstorder_MeanAbsoluteDeviation | -0.00306435 | 0.00122763 | 0.97522251 | 3 | 0.003970108 | -0.01009881 |
| logarithm_firstorder_10Percentile | -0.00306435 | 0.00235894 | 0.92332439 | 3 | 0.010452629 | -0.01658133 |
| original_shape2D_MeshSurface | -0.00306435 | 0.00296827 | 0.8921702 | 3 | 0.013944143 | -0.02007285 |
| wavelet-HH_glrlm_HighGrayLevelRunEmphasis | -0.00317785 | 0.00277308 | 0.90721081 | 3 | 0.012712194 | -0.01906789 |
| wavelet-HL_glcm_MCC | -0.00317785 | 0.00321812 | 0.88533732 | 3 | 0.015262364 | -0.02161806 |
| wavelet-HL_glszm_LowGrayLevelZoneEmphasis | -0.00317785 | 0.00264469 | 0.91355733 | 3 | 0.011976519 | -0.01833221 |
| wavelet-HL_gldm_DependenceNonUniformity | -0.00317785 | 0.0010945 | 0.98132992 | 3 | 0.003093768 | -0.00944946 |
| original_firstorder_Range | -0.00317785 | 0.00285546 | 0.9031386 | 3 | 0.013184278 | -0.01953997 |
| square_glszm_GrayLevelNonUniformity | -0.00329134 | 0.00137605 | 0.97318962 | 3 | 0.004593565 | -0.01117625 |
| gradient_glszm_SmallAreaEmphasis | -0.00329134 | 0.00174723 | 0.9587597 | 3 | 0.006720456 | -0.01330314 |
| wavelet-LH_glcm_Imc1 | -0.00329134 | 0.00260049 | 0.9201575 | 3 | 0.01160973 | -0.01819241 |
| wavelet-HL_glrlm_RunEntropy | -0.00329134 | 0.00540858 | 0.79879446 | 3 | 0.027700431 | -0.03428311 |
| wavelet-LH_glszm_LowGrayLevelZoneEmphasis | -0.00329134 | 0.00246312 | 0.92665517 | 3 | 0.0108226 | -0.01740528 |
| wavelet-LL_gldm_DependenceNonUniformity | -0.00340484 | 0.00265926 | 0.92157457 | 3 | 0.011833026 | -0.0186427 |
| logarithm_glszm_ZoneEntropy | -0.00340484 | 0.00207108 | 0.94781108 | 3 | 0.008462684 | -0.01527235 |
| logarithm_firstorder_90Percentile | -0.00340484 | 0.00034048 | 0.99834162 | 3 | -0.00145383 | -0.00535584 |
| logarithm_glszm_ZonePercentage | -0.00340484 | 0.00156029 | 0.96829291 | 3 | 0.005535808 | -0.01234548 |
| gradient_glszm_SmallAreaHighGrayLevelEmphasis | -0.00351833 | 0.00255552 | 0.93005809 | 3 | 0.011125067 | -0.01816173 |
| original_gldm_HighGrayLevelEmphasis | -0.00351833 | 0.00255552 | 0.93005809 | 3 | 0.011125067 | -0.01816173 |
| gradient_glrlm_RunLengthNonUniformity | -0.00351833 | 0.00285546 | 0.91679318 | 3 | 0.012843795 | -0.01988045 |
| exponential_firstorder_Skewness | -0.00351833 | 0.00277308 | 0.92045737 | 3 | 0.012371711 | -0.01940837 |
| wavelet-LL_gldm_SmallDependenceHighGrayLevelEmphasis | -0.00363182 | 0.00658757 | 0.77979897 | 3 | 0.034115688 | -0.04137934 |
| original_glrlm_HighGrayLevelRunEmphasis | -0.00363182 | 0.00437801 | 0.85634832 | 3 | 0.021454631 | -0.02871828 |
| squareroot_firstorder_Range | -0.00363182 | 0.00375047 | 0.88225479 | 3 | 0.017858806 | -0.02512245 |
| wavelet-HH_glcm_Autocorrelation | -0.00363182 | 0.00226705 | 0.94547684 | 3 | 0.009358628 | -0.01662228 |
| wavelet-LL_firstorder_Kurtosis | -0.00363182 | 0.00308947 | 0.9106616 | 3 | 0.014071179 | -0.02133483 |
| squareroot_firstorder_10Percentile | -0.00374532 | 0.00501564 | 0.83743813 | 3 | 0.024994826 | -0.03248546 |
| wavelet-LL_glszm_HighGrayLevelZoneEmphasis | -0.00374532 | 0.00547956 | 0.82094851 | 3 | 0.027653186 | -0.03514382 |
| gradient_ngtdm_Contrast | -0.00385881 | 0.00264469 | 0.93632795 | 3 | 0.011295552 | -0.01901318 |
| squareroot_firstorder_RobustMeanAbsoluteDeviation | -0.00385881 | 0.00323609 | 0.91255331 | 3 | 0.01468432 | -0.02240195 |
| squareroot_glszm_SmallAreaEmphasis | -0.00385881 | 0.00437801 | 0.86680207 | 3 | 0.021227642 | -0.02894527 |
| wavelet-LL_gldm_LargeDependenceHighGrayLevelEmphasis | -0.00385881 | 0.00246312 | 0.94339445 | 3 | 0.010255128 | -0.01797275 |
| original_glcm_JointAverage | -0.00385881 | 0.00019658 | 0.99956803 | 3 | -0.0027324 | -0.00498523 |
| wavelet-LL_firstorder_Minimum | -0.00385881 | 0.00444371 | 0.86426713 | 3 | 0.021604148 | -0.02932177 |
| wavelet-LL_gldm_GrayLevelNonUniformity | -0.00385881 | 0.00308947 | 0.91851107 | 3 | 0.01384419 | -0.02156182 |
| wavelet-HL_glszm_SizeZoneNonUniformityNormalized | -0.00397231 | 0.00618831 | 0.80902141 | 3 | 0.031487411 | -0.03943203 |
| gradient_glszm_SizeZoneNonUniformityNormalized | -0.00397231 | 0.00160906 | 0.97470993 | 3 | 0.005247797 | -0.01319241 |
| squareroot_glcm_MaximumProbability | -0.00397231 | 0.00250974 | 0.94435713 | 3 | 0.010408798 | -0.01835341 |
| squareroot_glszm_HighGrayLevelZoneEmphasis | -0.00397231 | 0.00216236 | 0.95690193 | 3 | 0.008418258 | -0.01636287 |
| squareroot_glrlm_ShortRunHighGrayLevelEmphasis | -0.00397231 | 0.00239148 | 0.94871795 | 3 | 0.009731123 | -0.01767574 |
| squareroot_gldm_HighGrayLevelEmphasis | -0.00397231 | 0.00239148 | 0.94871795 | 3 | 0.009731123 | -0.01767574 |
| wavelet-LH_glcm_JointAverage | -0.00397231 | 0.00160906 | 0.97470993 | 3 | 0.005247797 | -0.01319241 |
| wavelet-HH_firstorder_Energy | -0.0040858 | 0.00480311 | 0.86072217 | 3 | 0.023436555 | -0.03160816 |
| original_firstorder_90Percentile | -0.0040858 | 0.00090084 | 0.9920899 | 3 | 0.00107608 | -0.00924768 |
| squareroot_glrlm_HighGrayLevelRunEmphasis | -0.0040858 | 0.0020429 | 0.96291005 | 3 | 0.007620247 | -0.01579185 |
| wavelet-HL_glcm_Imc2 | -0.0040858 | 0.00414216 | 0.88516444 | 3 | 0.019649237 | -0.02782084 |
| wavelet-HH_glszm_SmallAreaHighGrayLevelEmphasis | -0.0040858 | 0.00379146 | 0.8985267 | 3 | 0.017639705 | -0.02581131 |
| logarithm_glrlm_RunLengthNonUniformityNormalized | -0.0040858 | 0.00189573 | 0.9675719 | 3 | 0.006776952 | -0.01494856 |
| squareroot_glcm_JointAverage | -0.0040858 | 0.00068097 | 0.99543369 | 3 | -0.00018379 | -0.00798782 |
| wavelet-HH_glszm_HighGrayLevelZoneEmphasis | -0.0041993 | 0.00070877 | 0.99531859 | 3 | -0.00013795 | -0.00826064 |
| wavelet-LH_glcm_ClusterShade | -0.0041993 | 0.00364068 | 0.90809927 | 3 | 0.016662203 | -0.0250608 |
| wavelet-LL_firstorder_Range | -0.0041993 | 0.00498472 | 0.85903721 | 3 | 0.024363712 | -0.0327623 |
| wavelet-HH_glcm_MCC | -0.0041993 | 0.00379656 | 0.90226898 | 3 | 0.017555392 | -0.02595399 |
| wavelet-HH_firstorder_Minimum | -0.0041993 | 0.00239148 | 0.95338235 | 3 | 0.009504134 | -0.01790273 |
| logarithm_firstorder_RobustMeanAbsoluteDeviation | -0.00442629 | 0.00401424 | 0.90182704 | 3 | 0.018575761 | -0.02742833 |
| wavelet-HL_gldm_LargeDependenceHighGrayLevelEmphasis | -0.00442629 | 0.00412814 | 0.89779354 | 3 | 0.019228431 | -0.028081 |
| wavelet-LL_glszm_SizeZoneNonUniformityNormalized | -0.00442629 | 0.00265926 | 0.94889902 | 3 | 0.010811575 | -0.01966415 |
| wavelet-LL_glszm_SmallAreaEmphasis | -0.00442629 | 0.00265926 | 0.94889902 | 3 | 0.010811575 | -0.01966415 |
| wavelet-LL_glrlm_HighGrayLevelRunEmphasis | -0.00442629 | 0.004347 | 0.89007802 | 3 | 0.020482519 | -0.02933509 |
| exponential_glrlm_RunEntropy | -0.00442629 | 0.00412814 | 0.89779354 | 3 | 0.019228431 | -0.028081 |
| logarithm_glrlm_RunPercentage | -0.00442629 | 0.00272387 | 0.94677321 | 3 | 0.01118178 | -0.02003435 |
| original_firstorder_Maximum | -0.00453978 | 0.00264469 | 0.95152364 | 3 | 0.010614585 | -0.01969415 |
| wavelet-HL_glszm_SmallAreaLowGrayLevelEmphasis | -0.00453978 | 0.00321812 | 0.93274232 | 3 | 0.01390043 | -0.02297999 |
| original_shape2D_Perimeter | -0.00453978 | 0.00410467 | 0.90225899 | 3 | 0.018980457 | -0.02806002 |
| logarithm_glcm_Idm | -0.00453978 | 0.00580156 | 0.84596071 | 3 | 0.028703781 | -0.03778334 |
| wavelet-LH_gldm_LargeDependenceHighGrayLevelEmphasis | -0.00465327 | 0.00098289 | 0.99272583 | 3 | 0.000978801 | -0.01028535 |
| wavelet-LH_glrlm_LongRunHighGrayLevelEmphasis | -0.00465327 | 0.00098289 | 0.99272583 | 3 | 0.000978801 | -0.01028535 |
| exponential_glrlm_GrayLevelNonUniformity | -0.00465327 | 0.00409053 | 0.9061995 | 3 | 0.018785905 | -0.02809245 |
| squareroot_glcm_Autocorrelation | -0.00465327 | 0.00443065 | 0.89474205 | 3 | 0.020734832 | -0.03004138 |
| wavelet-HL_glszm_GrayLevelVariance | -0.00465327 | 0.00128905 | 0.98768072 | 3 | 0.002733123 | -0.01203967 |
| original_glcm_Autocorrelation | -0.00476677 | 0.00401424 | 0.91200238 | 3 | 0.018235278 | -0.02776882 |
| wavelet-HL_firstorder_Range | -0.00476677 | 0.00442629 | 0.89843105 | 3 | 0.020596337 | -0.03012988 |
| wavelet-HH_glszm_LowGrayLevelZoneEmphasis | -0.00476677 | 0.00156029 | 0.98304589 | 3 | 0.004173874 | -0.01370741 |
| original_shape2D_MajorAxisLength | -0.00476677 | 0.00379146 | 0.91932961 | 3 | 0.016958738 | -0.02649228 |
| exponential_gldm_GrayLevelNonUniformity | -0.00476677 | 0.0032835 | 0.93580096 | 3 | 0.014048072 | -0.02358161 |
| original_glszm_SizeZoneNonUniformityNormalized | -0.00488026 | 0.00208039 | 0.97221395 | 3 | 0.007040593 | -0.01680112 |
| wavelet-HH_glcm_Idmn | -0.00488026 | 0.00572107 | 0.86120428 | 3 | 0.027902092 | -0.03766262 |
| square_glszm_SmallAreaLowGrayLevelEmphasis | -0.00499376 | 0.00137605 | 0.98780608 | 3 | 0.002891148 | -0.01287866 |
| squareroot_firstorder_Kurtosis | -0.00499376 | 0.00285546 | 0.95305485 | 3 | 0.011368366 | -0.02135588 |
| exponential_glrlm_RunVariance | -0.00499376 | 0.00289578 | 0.95190642 | 3 | 0.011599372 | -0.02158689 |
| multiple pulmonary metastases | -0.00510725 | 0.00345553 | 0.93765706 | 3 | 0.014693318 | -0.02490782 |
| wavelet-LL_gldm_HighGrayLevelEmphasis | -0.00510725 | 0.00581818 | 0.86610784 | 3 | 0.028231589 | -0.03844609 |
| original_glrlm_ShortRunHighGrayLevelEmphasis | -0.00510725 | 0.00683516 | 0.83755486 | 3 | 0.034058963 | -0.04427347 |
| wavelet-HL_glszm_SmallAreaEmphasis | -0.00533424 | 0.0043113 | 0.91732052 | 3 | 0.01936997 | -0.03003845 |
| original_shape2D_MinorAxisLength | -0.00544774 | 0.00401424 | 0.92843483 | 3 | 0.017554311 | -0.02844978 |
| wavelet-LL_glcm_JointAverage | -0.00544774 | 0.00578822 | 0.87768356 | 3 | 0.027719403 | -0.03861487 |
| original_glszm_SmallAreaEmphasis | -0.00556123 | 0.00255552 | 0.96813388 | 3 | 0.009082166 | -0.02020463 |
| wavelet-HH_glrlm_LongRunHighGrayLevelEmphasis | -0.00567473 | 0.00019658 | 0.99980012 | 3 | -0.00454831 | -0.00680114 |
| wavelet-LL_glrlm_ShortRunHighGrayLevelEmphasis | -0.00567473 | 0.00579156 | 0.88411568 | 3 | 0.027511536 | -0.03886099 |
| square_glrlm_ShortRunLowGrayLevelEmphasis | -0.00567473 | 0.0043381 | 0.9241555 | 3 | 0.019183089 | -0.03053254 |
| wavelet-HL_glrlm_LongRunHighGrayLevelEmphasis | -0.00578822 | 0.00306435 | 0.95895686 | 3 | 0.011770854 | -0.02334729 |
| exponential_glrlm_RunLengthNonUniformity | -0.00590171 | 0.0045341 | 0.92356289 | 3 | 0.020079191 | -0.03188262 |
| exponential_firstorder_10Percentile | -0.00590171 | 0.00394628 | 0.93885373 | 3 | 0.016710911 | -0.02851434 |
| gradient_glszm_HighGrayLevelZoneEmphasis | -0.0062422 | 0.00019658 | 0.99983479 | 3 | -0.00511578 | -0.00736861 |
| squareroot_ngtdm_Complexity | -0.00635569 | 0.00167957 | 0.98875222 | 3 | 0.003268403 | -0.01597979 |
| original_gldm_SmallDependenceHighGrayLevelEmphasis | -0.00635569 | 0.0058612 | 0.89942979 | 3 | 0.027229616 | -0.039941 |
| wavelet-LL_glcm_Autocorrelation | -0.00646919 | 0.0061287 | 0.89549018 | 3 | 0.02864896 | -0.04158733 |
| wavelet-HH_glszm_SmallAreaEmphasis | -0.00669618 | 0.00865614 | 0.84388451 | 3 | 0.042904474 | -0.05629682 |
| wavelet-HH_glszm_SizeZoneNonUniformityNormalized | -0.00680967 | 0.00736578 | 0.87476584 | 3 | 0.035397089 | -0.04901643 |
| wavelet-LL_firstorder_10Percentile | -0.00680967 | 0.00180167 | 0.98872641 | 3 | 0.003514095 | -0.01713343 |
| wavelet-LH_gldm_HighGrayLevelEmphasis | -0.00703666 | 0.00171373 | 0.9903983 | 3 | 0.0027832 | -0.01685652 |
| wavelet-HH_glcm_Idn | -0.00703666 | 0.00489081 | 0.9348549 | 3 | 0.020988222 | -0.03506154 |
| original_shape2D_Elongation | -0.00715015 | 0.00265926 | 0.97842736 | 3 | 0.008087707 | -0.02238801 |
| square_glszm_SizeZoneNonUniformityNormalized | -0.00737714 | 0.00193607 | 0.98890149 | 3 | 0.00371676 | -0.01847104 |
| wavelet-LL_glszm_SmallAreaHighGrayLevelEmphasis | -0.00749064 | 0.00392665 | 0.95966531 | 3 | 0.015009487 | -0.02999076 |
| wavelet-HL_firstorder_Skewness | -0.00760413 | 0.00153533 | 0.99334104 | 3 | 0.001193452 | -0.01640171 |
| square_ngtdm_Busyness | -0.00771763 | 0.00250974 | 0.98325496 | 3 | 0.006663479 | -0.02209873 |
| gradient_glszm_LowGrayLevelZoneEmphasis | -0.00964703 | 0.00676126 | 0.93396728 | 3 | 0.029095752 | -0.04838982 |
| age | -0.01430031 | 0.00918044 | 0.94285011 | 3 | 0.038304604 | -0.06690522 |

The feature name. ‘importance’: The estimated feature importance score. ‘stddev’: The standard deviation of the feature importance score. If NaN, then not enough num_shuffle_sets were used to calculate a variance. ‘p_value’: P-value for a statistical t-test of the null hypothesis: importance = 0, vs the (one-sided) alternative: importance > 0.

Features with low p-value appear confidently useful to the predictor, while the other features may be useless to the predictor (or even harmful to include in its training data). A p-value of 0.01 indicates that there is a 1% chance that the feature is useless or harmful, and a 99% chance that the feature is useful. A p-value of 0.99 indicates that there is a 99% chance that the feature is useless or harmful, and a 1% chance that the feature is useful.

‘n’: The number of shuffles performed to estimate importance score (corresponds to sample-size used to determine confidence interval for true score). ‘p99_high’: Upper end of 99% confidence interval for true feature importance score. ‘p99_low’: Lower end of 99% confidence interval for true feature importance score.
